# Techniques for harvesting the saphenous vein in coronary artery bypass grafting: a network systematic review and meta-analysis

**DOI:** 10.1136/openhrt-2025-003728

**Published:** 2025-12-18

**Authors:** Chuang Liu, Ming-xuan Zhang, Xin-liang Guan, Song-hao Jia, Wen-jie Tang, Xiao-long Wang, Wen-jian Jiang, Hong-jia Zhang

**Affiliations:** 1 Department of Cardiovascular Surgery, Beijing An Zhen Hospital, Capital Medical University, Beijing, China; 2 Beijing Laboratory of Cardiovascular Precision Medicine, Beijing Municipal Education Commission, Beijing, China; 3 Key Lab of Medical Engineering for Cardiovascular Disease, Ministry of Education, Beijing, China; 4 Department of Epidemiology and Biostatistics, School of Public Health, Peking University Health Science Center, Beijing, China

**Keywords:** Coronary Artery Bypass, Coronary Stenosis, Meta-Analysis

## Abstract

**Objective:**

The optimal technique for harvesting the saphenous vein (SVG) in coronary artery bypass grafting (CABG) remains undetermined. This study aimed to assess the efficacy of open vein harvesting (OVH), endoscopic vein harvesting (EVH) and no-touch vein harvesting (NT) in CABG using a network meta-analysis of randomised controlled trials (RCTs).

**Methods:**

RCTs evaluating the outcomes in patients undergoing CABG with the SVG using OVH, EVH or NT were identified through a systematic search of PubMed, Web of Science and the Cochrane Central Registry up to August 2025. The outcomes analysed included graft failure, graft occlusion, mortality, revascularisation, myocardial infarction (MI) and leg wound infection rates.

**Results:**

Data from 26 RCTs involving 7254 patients meeting the inclusion criteria were analysed. The network meta-analysis indicated that the graft failure rate in the NT group was significantly lower than in the OVH group (relative risk (RR) 0.62; 95% CI 0.40 to 0.96) and the EVH group (RR 0.41; 95% CI 0.21 to 0.80). The graft occlusion rate in the NT group was significantly lower than in the OVH group (RR 0.66, 95% CI 0.52 to 0.84). However, the leg wound infection rate in the NT group was the highest. No significant differences were observed in mortality, revascularisation and MI rates among the three groups.

**Conclusion:**

NT was associated with lower graft failure and graft occlusion rates, yet it had higher leg wound infection rates, while mortality, revascularisation and MI rates remained comparable among the three techniques. These findings require cautious interpretation, and it is important to balance harvest site complications and the desirability of long-term graft patency.

**WHAT IS ALREADY KNOWN ON THIS TOPIC**

- Coronary artery bypass grafting is the most frequently performed surgical procedure for coronary artery disease. compared with open vein harvesting, no-touch vein harvesting (NT) prioritises reducing vein graft damage, while endoscopic vein harvesting (EVH) emphasises leg wound protection. However, the optimal technique for harvesting saphenous vein grafts (SVGs) remains a topic of debate.

**WHAT THIS STUDY ADDS**

- This study provides high-quality clinical evidence for the comparison of three SVGs. The NT group demonstrated the best graft patency, but it also had the highest incidence of leg wound infection. In contrast, EVH was associated with the highest midterm graft failure rate, yet it had the lowest rate of leg wound infection. Moreover, mortality, revasculaation and myocardial infarction rates remained comparable among the three techniques.

**HOW THIS STUDY MIGHT AFFECT RESEARCH, PRACTICE OR POLICY**

- These findings require cautious interpretation. Given that there was no significant difference in clinical outcomes among the three groups, we should cautiously assess the clinical significance of NT in improving graft patency rate. Besides, it is important to balance harvest site complications and the need for long-term graft patency.

## Introduction

 Coronary artery bypass grafting (CABG) is the most frequently performed surgical procedure for coronary artery disease. About one million patients undergo CABG annually worldwide.^1^ Unlike medical therapy and percutaneous coronary intervention, CABG establishes an extra-anatomic bypass conduit, leading to improved survival in selected patients.^2 3^

The saphenous vein graft (SVG) remains the primary conduit in CABG procedures;^1^ however, the optimal technique for harvesting SVGs remains a topic of debate. Conventionally, the saphenous vein is harvested using the open vein harvesting (OVH) technique. However, this method is associated with a higher incidence of leg wound complications^4^ and a relatively high occlusion rate, ranging from 10% to 15% within the first year postsurgery.^5^ To mitigate these limitations, endoscopic vein harvesting (EVH) was introduced, allowing for reduced leg wound complications by minimising incision size.^6^ Despite this advantage, a higher risk of vein graft failure has been reported to be associated with EVH in cohort studies.^7^

No-touch vein harvesting (NT) has emerged as an alternative technique aimed at reducing saphenous vein damage by preserving the adventitia and avoiding manual dissection during harvesting.^8^ This approach has been shown to enhance vein graft patency and lower occlusion rates.^9 10^ However, NT has been associated with a higher incidence of leg wound complications,^11 12^ limiting its widespread adoption.

The long-term effects of different harvesting techniques remain inadequately studied.^13 14^ Previous meta-analyses primarily included observational studies and compared these techniques in pairs rather than collectively.^15^,^19^ To date, only one network meta-analysis has evaluated the three techniques exclusively using randomised controlled trials (RCTs),^20^ but it did not incorporate the latest RCTs, lacked data on comparison of revascularation and failed to distinguish between graft failure and graft occlusion. Therefore, this study aimed to determine the optimal vein harvesting technique by conducting a network meta-analysis of RCTs, which may provide higher-quality evidence on the graft patency and clinical outcomes of OVH, EVH and NT.

## Methods

### Literature search strategy and selection criteria

This study adhered to the Preferred Reporting Items for Systematic Reviews and Meta-Analyses guidelines and Assessing the methodological quality of systematic reviews guidelines for reporting systematic reviews.^21 22^ A comprehensive literature search was conducted by two independent reviewers, who evaluated all relevant studies published in PubMed, Web of Science and the Cochrane Central Registry from 1 January 1970 to 1 August 2025. The focus was on RCTs that compared clinical outcomes in patients undergoing CABG with SVG using OVH, EVH or NT. This review has been registered in the International Prospective Register of Systematic Reviews.

Studies meeting the following inclusion criteria were considered: (1) RCTs; (2) studies reporting baseline and postoperative outcomes in patients undergoing CABG with SVG using OVH, EVH or NT, evaluating at least one of the following outcomes: graft failure, graft occlusion, mortality, revascularisation, myocardial infarction (MI) or leg wound infection. Exclusion criteria included: (1) reviews or meta-analyses; (2) studies lacking outcomes of interest or preoperative and postoperative data on the outcome measures; (3) study protocols, basic science or animal studies; (4) duplicate or same population; (5) not RCTs; (6) unavailable full English texts.

The database search strategy is presented in online supplemental table S1. Titles and abstracts of searched studies were independently screened for inclusion by two reviewers (CL and M-xZ).

### Data extraction and quality assessment

Data extracted included study characteristics (eg, publication year, author, number of patients and follow-up duration), patient demographics (eg, age, sex and comorbidities) and clinical outcomes. Information was obtained from article texts, tables and figures in different groups. Two independent investigators reviewed each selected article, and any discrepancies were resolved through discussion with a third reviewer. Study appraisal and quality scoring were conducted following the Cochrane risk of bias tool (ROB2)^23^ and Review Manager (V.5.4). We used five domains to assess the risks of bias, which included randomisation process, deviations from intended interventions, missing outcome data, measurement of the outcome and selection of the report result. All included studies were classified into a low, high or unclear risk for each of bias.

### Outcomes

The assessed outcomes included graft failure, graft occlusion, mortality, revascularisation, MI and leg wound infection in patients undergoing CABG with SVG using OVH, EVH or NT. Graft failure was defined as stenosis greater than 50% of the graft detected on coronary angiography or CT angiography, while graft occlusion was defined as complete occlusion. Mortality was defined as all-cause mortality after surgery and during follow-up. Leg wound infection was identified in RCTs if patients had such infections or needed extra antibiotics because of leg wounds. Outcomes were analysed across all postoperative follow-up periods.

### Statistical analysis

The statistical analysis followed five key steps^24^: visualising the network relationship, assessing consistency assumptions, illustrating comparative effectiveness using a network forest plot, ranking interventions based on cumulative rankings and evaluating publication bias. A pairwise meta-analysis was then performed to compare OVH and EVH as well as OVH and NT. However, due to the limited availability of studies directly comparing EVH and NT, a meaningful pairwise meta-analysis between these groups was not feasible, as only one study reported this comparison.^25^ A random-effects model was used for pairwise meta-analyses, calculating relative risks (RRs) with 95% CIs for direct treatment comparisons. Heterogeneity was evaluated using *I*^2^ and *τ*^2^ statistics.

A network meta-analysis was conducted using the frequentist approach to compare the efficacy and safety of OVH, EVH and NT in patients undergoing CABG. RRs with 95% CIs based on a random-effects model. To establish a comparative hierarchy of procedural efficacy and safety, ‘rankograms’ with surface under the cumulative ranking curve (SUCRA) probabilities were reported. SUCRA provides an estimation of cumulative ranking probabilities,^26^ where higher SUCRA values indicate superior surgical techniques.^27^ A subgroup analysis was conducted using solely the studies with a minimum follow-up period of 1 year. Additionally, a sensitivity analysis was conducted by excluding the study with the largest sample size. All statistical analyses were performed using Stata MP V.17.0 (Stata).

### Certainty of evidence

Evidence of this network meta-analysis was evaluated following the approach proposed by the Grading of Recommendations Assessment, Development and Evaluations (GRADE) scoring system.^28^ Evidence was graded high, moderate, low or very low based on study design, adjusted for five downgrading factors (risk of bias, imprecision, inconsistency, indirectness, publication bias) and three upgrading factors (large effect, dose-response, residual confounding). Randomised trials start with high evidence quality, subject to upgrading or downgrading based on the above factors.

## Results

### Patient characteristics

**Figure 1:**
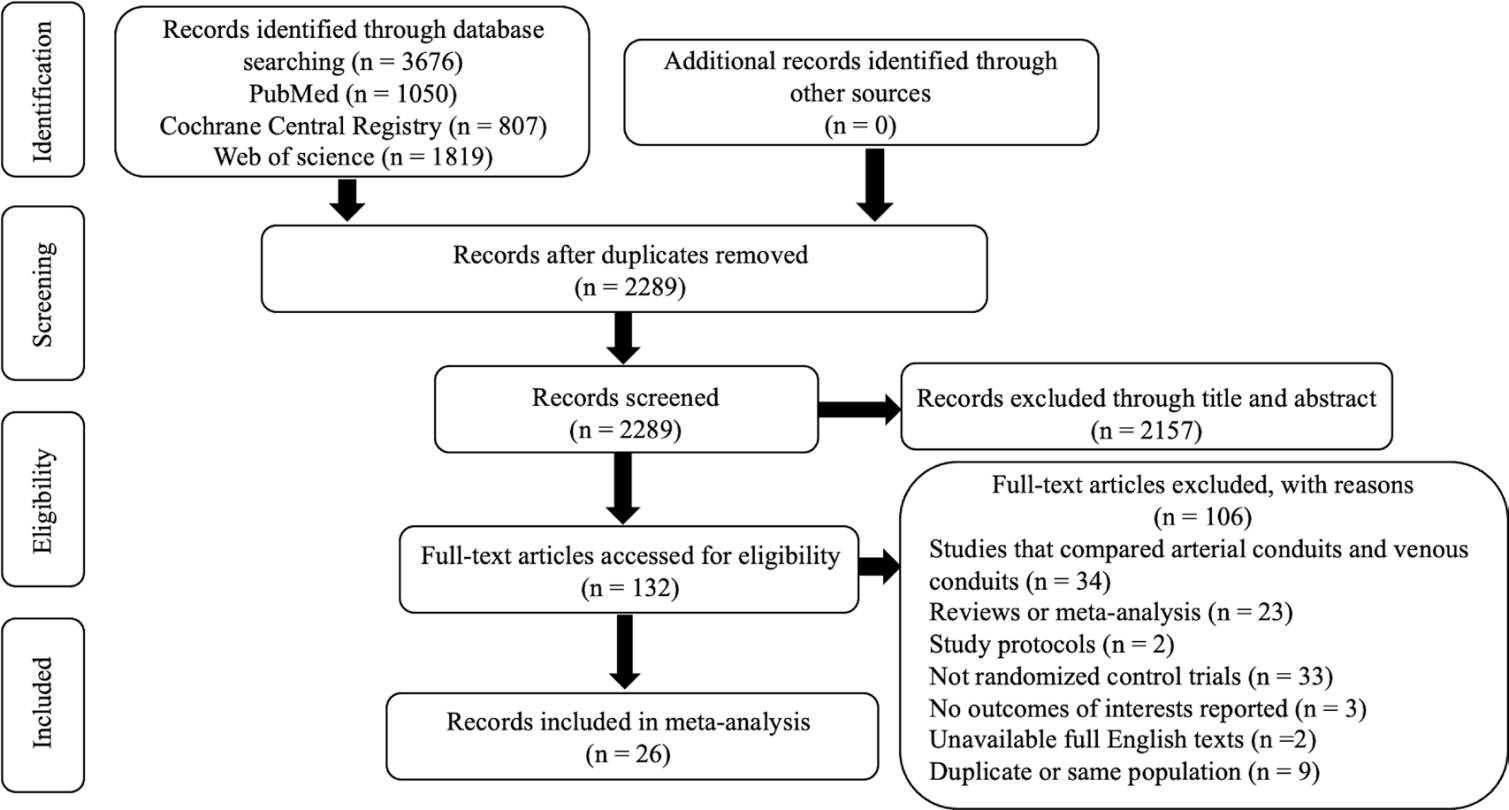
PRISMA flowchart for OVH, EVH and NT. Literature search for network meta-analysis of OVH, EVH and NT. EVH, endoscopic vein harvesting; NT, no-touch vein harvesting; OVH, open vein harvesting; PRISMA, Preferred Reporting Items for Systematic Reviews and Meta-Analysis.

A systematic electronic database search identified 3676 studies (figure 1). Duplicates, irrelevant studies and those lacking accessible data were excluded. Ultimately, 26 studies involving 7254 patients met the inclusion criteria for analysis. Among these patients, 3546 underwent OVH, 1583 received EVH and 2125 underwent NT. Study characteristics and patients’ characteristics were summarised in table 1.

**Table 1:** Summary of studies included

First author	Year	Country	Treatment arms	Study period	No. of patients	Age (year old)	Female (%)	DM (%)	HTN (%)	Previous MI (%)	HLP (%)
Thelin^47^	2025	Sweden	OVH/NT	3.5 years	446/454	67.1/66.9	12.1/11.9	25.8/28.5	NR	NR	NR
Tian^29^	2025	China	OVH/NT	1 year	1318/1337	60.8/60.9	21.8/21.4	35.1/36.2	61.8/64.5	20.5/20.9	69.2/68.0
Zenati^4348^,^51^	2021	USA	OVH/EVH	4.7 years	574/576	66.6/66.2	0.5/0.5	51.8/48.6	89.7/90.6	36.1/38.1	87.5/85.4
Hou^52^	2021	China	OVH/NT	3 months	50/50	59.8/61.0	6/8	40/36	60/58	10/8	22/24
Deb^12^	2019	Canada	OVH/NT	1 year	123/127	64.0/65.6	8.1/16.5	34.1/34.6	83.7/75.6	39.8/39.4	NR
Peterson^53 54^	2017	Norway	OVH/NT	6 months	51/49	65.0/63.4	18/7	4/2	NR	41/29	NR
Andreason^36 55^	2015	Denmark	OVH/EVH	6.3 years	62/64	64.6/64.7	17/11	14/11	NR	NR	77/73
Samano, Souza, Johansson^10 31 56 57^	2015	Sweden	OVH/NT	16 years	52/52	58/58	13/16	25/37	36/55	63/56	63/64
Chernyavskiy^58^	2015	Russia	OVH/EVH	1 week	115/113	60/61	17/18	17/19	95/84	NR	NR
Brat^59^	2012	Czech	OVH/EVH	1 month	50/50	66.2/65.0	10/16	44/40	NR	NR	NR
Krishnamoorthy^60^	2012	UK	OVH/EVH	6 weeks	50/50	68/64	22/10	24/20	NR	NR	NR
Wang^61^	2011	China	OVH/EVH	4 months	20/20	64/59	10/5	25/35	35/40	NR	NR
Au^62^	2008	China	OVH/EVH	3 weeks	60/54	64.5/65.5	18/15	40/46	NR	NR	NR
Yun^37^	2005	USA	OVH/EVH	6 months	100/100	64/64	7/7	35/36	74/76	44/48	NR
Bonde^25 63^	2004	UK	NT/EVH	3 years	52/56	60.5/59.0	23/19	12/14	NR	NR	NR
Perrault^38^	2004	Canada	OVH/EVH	3 months	20/20	64/57	6/11	55/35	75/70	NR	NR
Kiaii^64^	2002	Canada	OVH/EVH	8 weeks	72/72	64.3/62.5	15/21	25/29	60/57	NR	63.9/62.5
Schurr^65^	2002	Switzerland	OVH/EVH	3 months	60/80	64/64	36/36	45/33	NR	NR	NR
Fabricius^66^	2000	Germany	OVH/EVH	In-hospital	30/31	64.2/65.3	23/25	6/5	NR	NR	NR
Cisowski^67^	2000	Poland	OVH/EVH	1 week	15/30	61.4/59.7	27/23	6.6/16.7	NR	NR	NR
Carpino^68^	2000	USA	OVH/EVH	2 weeks	66/66	68/66	47/38	69/63	NR	NR	NR
Puskas^69^	1999	USA	OVH/EVH	1 month	50/47	65.6/65.4	34/28	28/32	NR	NR	NR
Hayward^70^	1999	USA	OVH/EVH	6 weeks	50/50	66/64	26/18	28/32	NR	NR	NR
Folliguet^71^	1998	France	OVH/EVH	In hospital	30/30	68.9/66.8	30/20	10/17	NR	NR	NR
Allen^72^	1998	USA	OVH/EVH	6 weeks	58/51	64.1/64.5	17.2/15.7	25.9/23.5	NR	NR	NR
Morris^73^	1998	USA	OVH/EVH	In-hospital	24/27	63.1/66.1	50/33	21/30	NR	NR	NR

DM, diabetes mellitus; EVH, endoscopic vein harvesting; HLP, hyperlipidaemia; HTN, hypertension; MI, myocardial infarction; NR, not reported; NT, no-touch vein harvesting; OVH, open vein harvesting.

**Figure 2:**
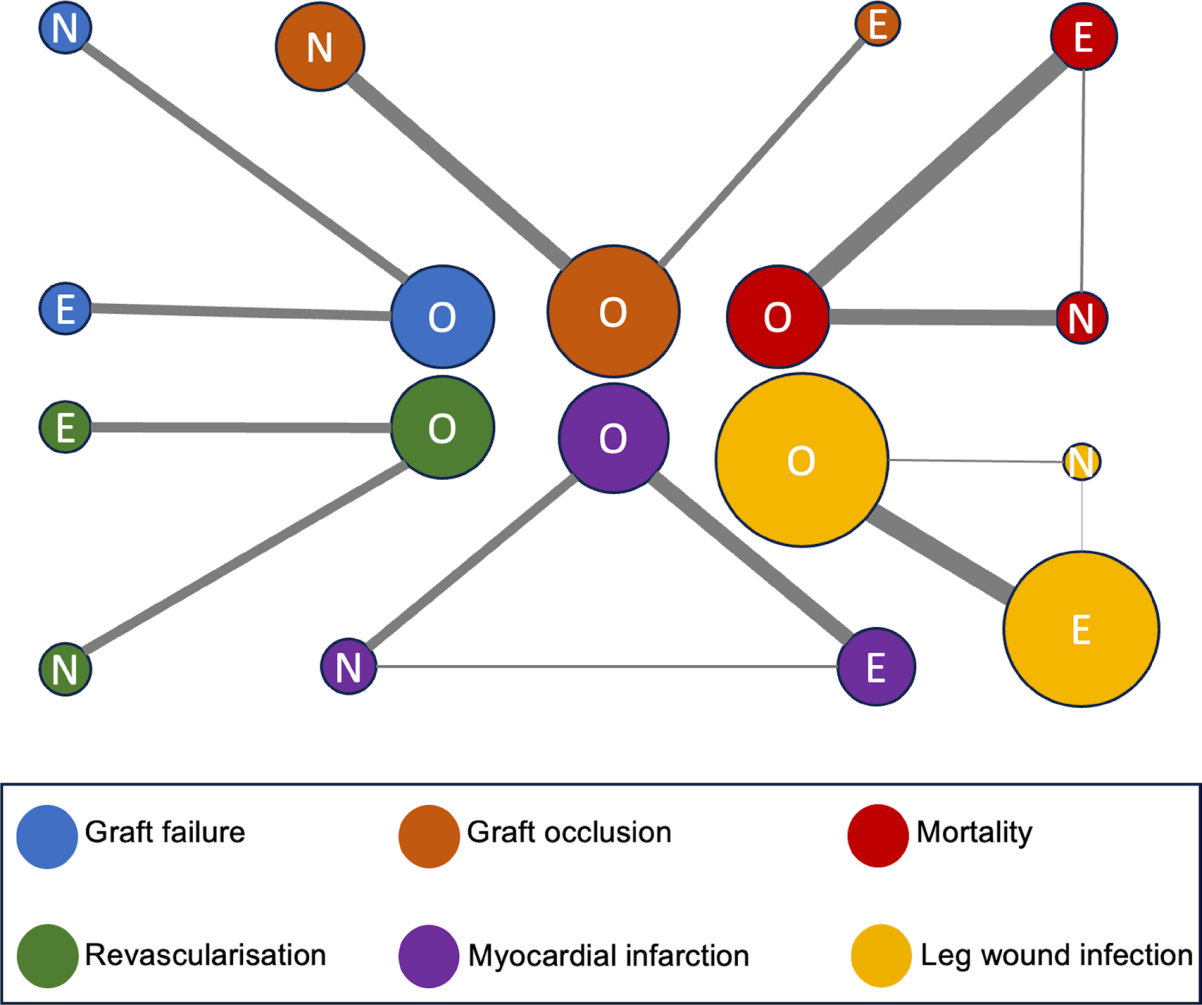
Network diagram for OVH, EVH and NT. Network meta-analysis comparisons for: (blue) graft failure, (orange) graft occlusion, (red) mortality, (green) revascularisation, (purple) myocardial infarction and (yellow) leg wound infection. The node size is proportional to the number of participants engaged, and the thickness of the continuous line connecting nodes is proportional to the number of directly comparing participants between the two treatments. E, endoscopic vein harvesting; N, no-touch vein harvesting; O, open vein harvesting.

The funnel plot did not indicate significant asymmetry, suggesting no publication bias in graft occlusion, mortality, revascularisation, MI and leg wound infection outcomes (online supplemental figures S1-2–S1-6). However, there was publication bias in graft failure (online supplemental figure S1-1). Study quality assessment findings were outlined in online supplemental figure S2. The complete evidence networks for all analysed outcomes are depicted in figure 2 and online supplemental figures S3-1–S3-6.

### Graft failure

**Figure 3:**
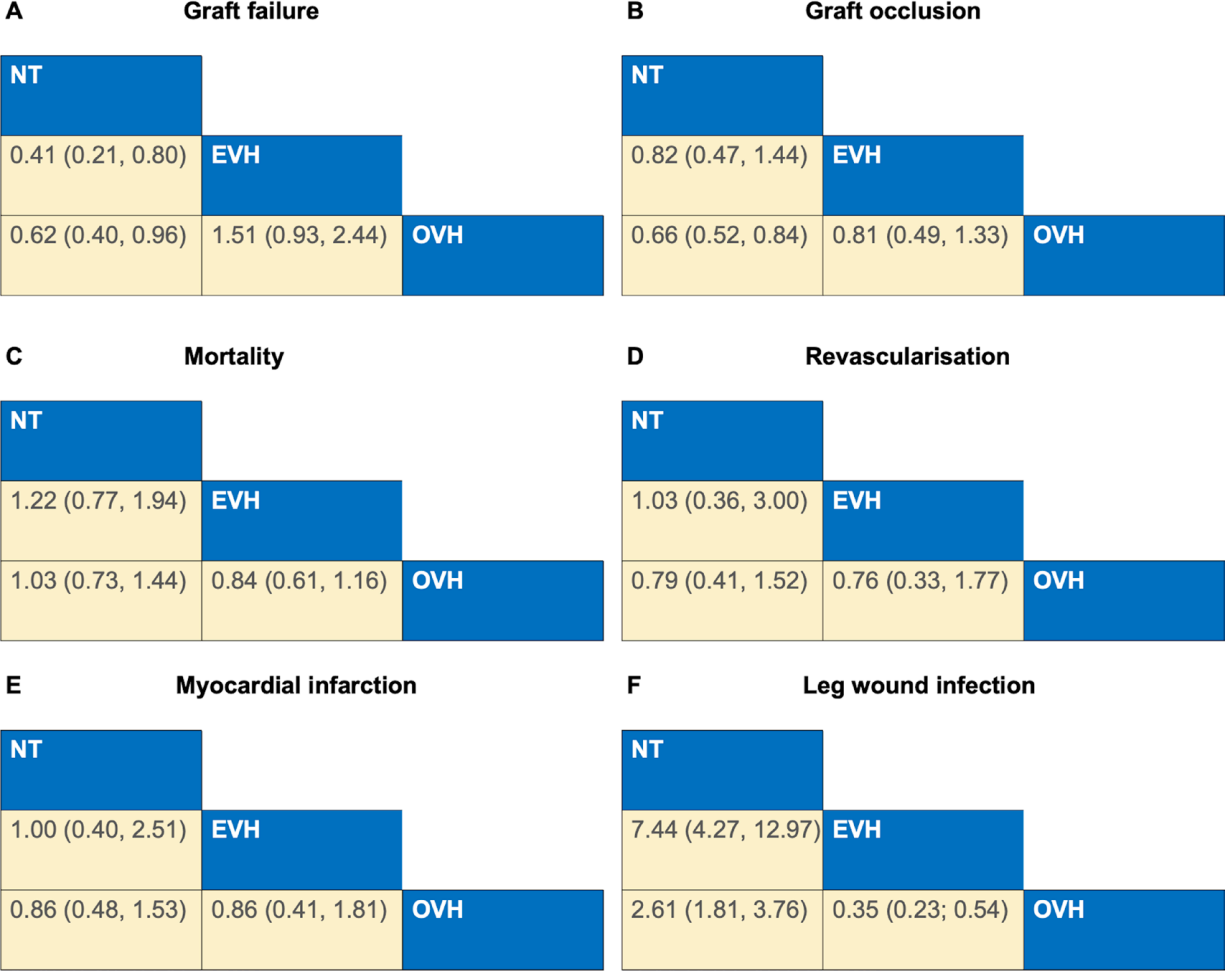
League tables for OVH, EVH and NT. Outcomes shown for (**A**) graft failure, (**B**) graft occlusion, (**C**) mortality, (**D**) revascularisation, (**E**) myocardial infarction and (**F**) leg wound infection following OVH, EVH and NT (RR and 95% CI). RR <1 means the treatment in top left is better. EVH, endoscopic vein harvesting; NT, no-touch vein harvesting; OVH, open vein harvesting; RR, relative risk.

**Figure 4:**
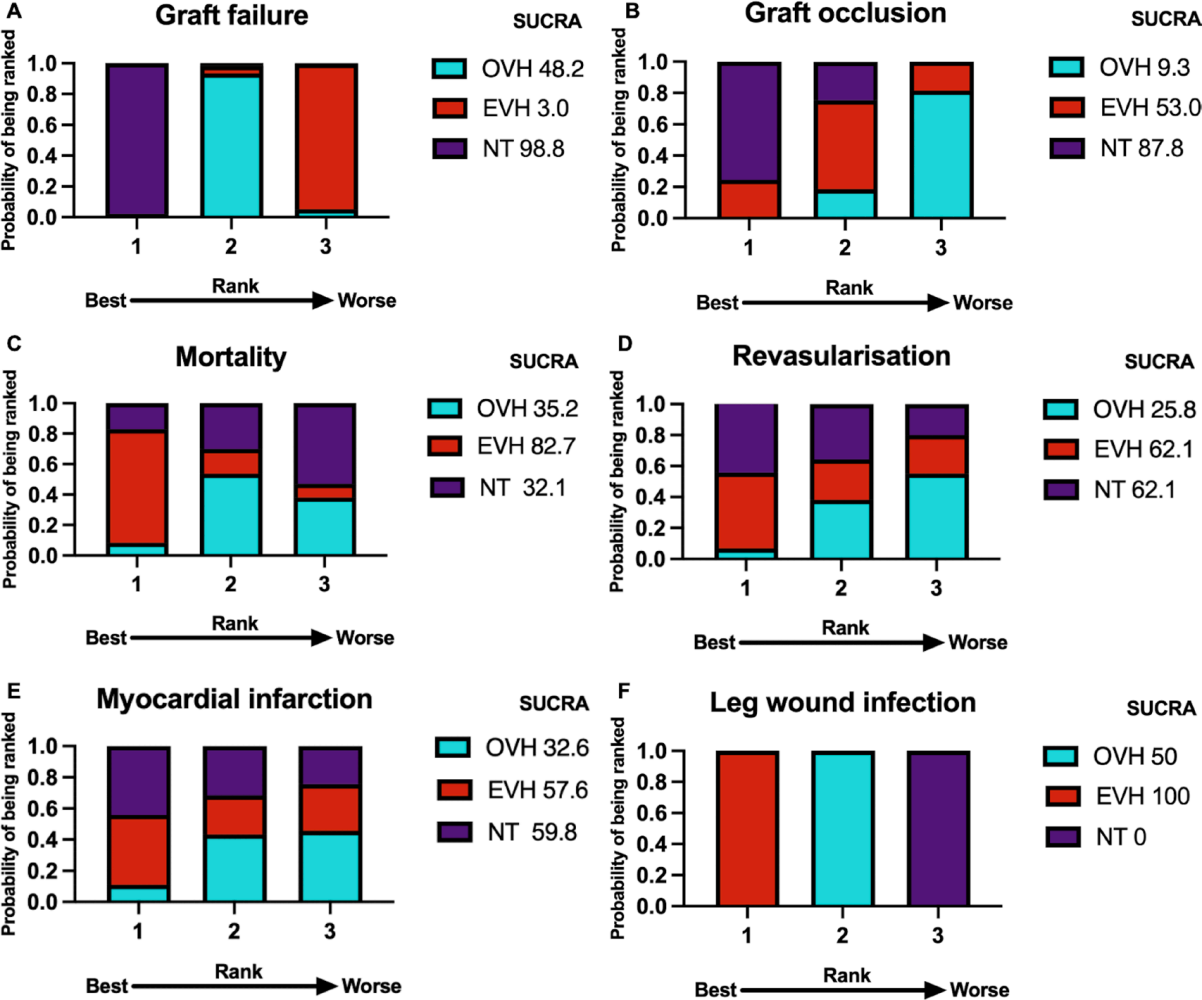
Rankograms for OVH, EVH and NT. Outcomes shown for (**A**) graft failure, (**B**) graft occlusion, (**C**) mortality, (**D**) revascularisation, (**E**) myocardial infarction and (**F**) leg wound infection following coronary artery bypass graft with OVH, EVH and NT. EVH, endoscopic vein harvesting; NT, no-touch vein harvesting; OVH, open vein harvesting; SUCRA, surface under the cumulative ranking.

Network meta-analysis demonstrated that the risk of graft failure in the NT group was significantly lower compared with the EVH group (RR 0.41; 95% CI 0.21 to 0.80) and lower than that in the OVH group (RR 0.62; 95% CI 0.40 to 0.96). However, no significant difference in graft failure rates was observed between the EVH and OVH groups (RR 1.51; 95% CI 0.93 to 2.44). The corresponding league table was presented in figure 3A and the forest plot was shown in online supplemental figure S4-1. Analysis of SUCRA values revealed that NT had the highest probability of achieving the lowest rate of graft failure (98.8%) (figure 4A, online supplemental figure S5-1). The study revealed no publication bias when comparing the OVH group with the NT group. However, publication bias was observed in the comparison between the OVH and EVH groups.

A subsequent pairwise meta-analysis was performed. The risk of graft failure in the OVH group was significantly higher than that in the NT group (RR 1.37; 95% CI 1.16 to 1.62; *I^2^*=7.9%, *P_heterogeneity_*=0.354) (online supplemental figure S6-2). However, no significant difference in graft failure risk was identified between the OVH and EVH groups (RR 0.85; 95% CI 0.58 to 1.25; *I^2^*=60.2%, *P_heterogeneity_*=0.056) (online supplemental figure S6-1), with notable heterogeneity between these groups. These findings suggested that NT was associated with a significantly lower graft failure rate compared with both OVH and EVH.

### Graft occlusion

Network meta-analysis demonstrated that the risk of graft occlusion in the NT group was significantly lower compared with the OVH group (RR 0.66; 95% CI 0.52 to 0.84). However, no significant difference in graft failure rates was observed between the EVH and OVH groups (RR 0.81; 95% CI 0.49 to 1.33) and between the NT and EVH groups (RR 0.82; 95% CI 0.47 to 1.44). The corresponding league table was presented in figure 3B and the forest plot was shown in online supplemental figure S4-2. Analysis of SUCRA values revealed that NT had the highest probability of achieving the lowest rate of graft occlusion (87.8%) (figure 4, online supplemental figure S5-2). The funnel plot did not suggest publication bias among the included studies (online supplemental figure S1-2).

A subsequent pairwise meta-analysis was performed. The risk of graft occlusion in the OVH group was significantly higher than that in the NT group (RR 1.45; 95% CI 1.16 to 1.81; *I^2^*=0%, *P_heterogeneity_*=0.988) (online supplemental figure S6-4). However, no significant difference in graft occlusion risk was identified between the OVH and EVH groups (RR 1.19; 95% CI 0.79 to 1.80; *I^2^*=0%, *P_heterogeneity_*=0.693) (online supplemental figure S6-3). These findings suggested that NT was associated with a significantly lower graft occlusion rate compared with OVH.

### Mortality

Network meta-analysis indicated no statistically significant difference in mortality rates among the three groups. The corresponding league table was presented in figure 3C and the forest plot was shown in online supplemental figure S4-3. Analysis of surface under the SUCRA values demonstrated that patients who underwent EVH had the highest probability of the lowest mortality rate (82.7%) (figure 4C and online supplemental figure S6-3). The funnel plot did not suggest publication bias among the included studies (online supplemental figure S1-3).

Subsequently, a pairwise meta-analysis was performed. No significant difference in mortality risk was observed between the OVH and EVH groups or between the OVH and NT groups (online supplemental figures S6-5, S6-6). These findings indicated no substantial difference in mortality rates across the three harvesting techniques.

### Revascularisation

Network meta-analysis indicated no statistically significant difference in the revascularisation rates among the three groups. The corresponding league table was presented in figure 3D and the forest plot was shown in online supplemental figure S4-4. Analysis of SUCRA values demonstrated that EVH and NT both had the highest probability of achieving the lowest revascularisation rate (62.1% and 62.1%, respectively) (figure 4D, online supplemental figure S5-4). The funnel plot analysis showed no evidence of publication bias in the included studies (online supplemental figure S1-4).

A subsequent pairwise meta-analysis was conducted. No significant difference in revascularisation risk was observed between the OVH and EVH groups, nor between the OVH and NT groups (online supplemental figures S6-7, S6-8). These findings indicated no substantial variation in revascularisation rates across the three harvesting groups.

### Myocardial Infarction

Network meta-analysis indicated no statistically significant difference in MI rates among the three groups. The corresponding league table was shown in figure 3E and the forest plot was shown in online supplemental figure S4-5. Analysis of SUCRA values revealed that patients who underwent NT had the highest probability of achieving the lowest MI rate (59.8%) (figure 4E, online supplemental figure S5-5). The funnel plot did not suggest publication bias in the included studies (online supplemental figure S1-5).

A subsequent pairwise meta-analysis was performed. The risk of MI was not significantly different between the OVH and EVH groups, nor between the OVH and NT groups (online supplemental figures S6-9, S6-10). These findings suggested no significant variation in MI rates across the three harvesting groups.

### Leg wound infection

Network meta-analysis demonstrated that the risk of leg wound infection in the NT group was significantly higher compared with the OVH group (RR 2.61; 95% CI 1.81 to 3.76) and the EVH group (RR 7.44; 95% CI 4.27 to 12.97). Besides, the risk of leg wound infection in the EVH group was significantly lower compared with the OVH group (RR 0.35; 95% CI 0.23 to 0.54). The corresponding league table was shown in figure 3F and the forest plot was shown in online supplemental figure S4-6. Analysis of SUCRA values revealed that patients who underwent EVH had the highest probability of achieving the lowest leg wound infection rate (100%) (figure 4F, online supplemental figure S5-6). The funnel plot did not suggest publication bias in the included studies (online supplemental figure S1-6).

A subsequent pairwise meta-analysis was performed. The risk of leg wound infection in the OVH group was significantly higher than that in the EVH group (RR 2.31; 95% CI 1.59 to 3.37; *I^2^*=0%, *P_heterogeneity_*=0.458) (online supplemental figure S6-11), but it was significantly lower than that in the NT group (RR 0.44, 95% CI 0.32 to 0.61; *I^2^*=0%, *P_heterogeneity_*=0.693) (online supplemental figure S6-12). These results indicate that the leg wound infection rates in different groups were as follows: EVH group <OVH group <NT group.

### Additional analysis

Results from the network and pairwise meta-analysis were consistent, confirming model reliability. A subgroup analysis was conducted using solely the studies with a minimum follow-up period of 1 year to compare the midterm outcomes of graft failure and graft occlusion. Network meta-analysis findings indicated that the risk of midterm graft failure in the NT group was significantly lower than that in the EVH group (RR 0.36; 95% CI 0.26 to 0.50) and that in the OVH group (RR 0.69; 95% CI 0.57 to 0.83), and this risk is significantly higher in the EVH group than in the OVH group (RR 1.91; 95% CI 1.45 to 2.53) (online supplemental figure S7-1). Besides, the pairwise meta-analysis exhibited a similar trend to that of the network meta-analysis (online supplemental figure S7-2, S7-3). There were only two studies which compared the midterm graft occlusion rates between the OVH group and the NT group. Pairwise meta-analysis findings indicated that the risk of midterm graft occlusion was significantly higher in the OVH group than in the NT group (RR 1.46; 95% CI 1.16 to 1.83; *I^2^*=0%, *P_heterogeneity_*=0.811) (online supplemental figure S7-4). Sensitivity analyses, conducted by excluding the study^29^ with the largest sample size, showed that the result that the graft failure and graft occlusion rate of NT were significantly higher than those in OVH was somewhat not robust (online supplemental figure S8).

### Certainty of evidence

According to the GRADE scoring system, the quality of evidence was moderate for graft failure, graft occlusion, mortality, revascularisation, MI and leg wound infection of the three groups. We provided a detailed breakdown of the quality of evidence for each outcome (online supplemental table S2-1–S2-6).

## Discussion

This network meta-analysis, which included 26 RCTs and a total of 7254 patients, demonstrated that NT was associated with a significantly lower graft failure rate compared with OVH and EVH, a significantly lower graft occlusion rate compared with OVH and a significantly higher leg wound infection rate compared with OVH and EVH. Additionally, the subgroup analysis indicated that these findings were also applicable to midterm clinical outcomes. However, mortality, revascularisation and MI rates were comparable among the three groups.

Graft stenosis and even occlusion remain major concerns in CABG, as SVGs exhibit relatively high failure rates. The incidence of graft failure ranges between 6.5% and 25% within the first year postsurgery and increases to about 40% after 10 years,^729^,^31^ significantly impacting patient prognosis. SVGs may occlude either early in the postoperative period or progressively narrow over time.^32^ Early graft failure is primarily attributed to thrombosis, conduit damage and vessel-conduit mismatch, while intimal hyperplasia is the predominant cause within the first year. Beyond this period, atherosclerosis becomes the primary mechanism leading to graft failure.^32^

To better assess long-term outcomes, a subgroup analysis was performed, including only studies with a minimum follow-up of 1 year. The findings confirmed that NT had the highest graft patency rate (figure 4A, B), and among patients with follow-up durations exceeding 1 year, the ranking of free from graft failure also remained NT>OVH>EVH (online supplemental figure S7-1–S7-3). Several studies have suggested that NT achieves a lower occlusion rate, nearly comparable to that of arterial grafts.^33^ Current guidelines also endorse NT in OVH, assigning it a Class IIa recommendation with a level of evidence B.^34^ These findings align with the results of the present study, further supporting NT as an optimal technique for improving long-term SVG patency.

Our study identified EVH as having the highest midterm graft failure rate (online supplemental figure S7-1), and we also found that EVH had higher midterm graft occlusion rate than OVH (online supplemental figure S7-4). The EXCEL (Evaluation of XIENCE Versus CABG for Effectiveness of Left Main Revascularisation) study^35^ and the study by Andreasen *et al*,^36^ both of which conducted 1 year follow-up on graft failure, reported a higher rate of graft failure in the EVH group compared with the OVH group (9.7% vs 5.4%, p=0.054; 42% vs 6%, p=0.001, respectively). However, two other RCTs with follow-up periods shorter than 1 year (3 and 6 months, respectively) did not find a significant difference in graft failure rates between the two groups.^37 38^ Similarly, a cohort study by Lopes *et al* involving 3000 CABG patients found comparable graft failure rates between OVH and EVH within the first year. However, beyond 1–1.5 years, the EVH group exhibited a significantly higher graft failure rate compared with OVH (46.7% vs 38.0%, p<0.001).^7^ This finding provided insight into the differences in RCT results with varying follow-up durations and explained the significant heterogeneity observed in pairwise meta-analyses comparing EVH and OVH in terms of graft failure.

Prior network meta-analyses have presented conflicting findings regarding graft failure rates among OVH, EVH and NT. A study by Yokoyama *et al* reported that NT had the lowest graft failure rate but found no significant difference between OVH and EVH.^39^ Conversely, Vuong *et al* found no significant differences in graft failure rates among the three techniques.^20^ The results of this study align more closely with those of Yokoyama *et al* However, sensitivity analyses revealed that the result that the graft patency rate in the NT group was higher than that in the OVH group lacked robustness (online supplemental figure S8).

In contrast to OVH, where the external tissue of the conduit was removed while the conduit was dilated, NT harvesting preserved vein integrity, maintained the vasa vasorum and nerves within the adventitia, prevented graft distension during harvesting,^32^ and retained perivascular adipose tissue, which may induce vasodilation through nitric oxide production.^40^ The higher graft failure rate observed in the EVH group compared with the OVH group may be attributed to EVH harvesting veins primarily from the thigh rather than the calf, resulting in thicker conduits.^39^ This discrepancy could contribute to conduit-related size mismatch, which impairs runoff and negatively affects graft patency.^32^ Additionally, EVH may cause more substantial conduit damage compared with OVH.^7^ Although histological examinations have indicated that veins harvested using EVH and OVH exhibit similar endothelial integrity,^25 41^ EVH has been reported to negatively impact the endothelium of SVGs by reducing esterase activity and nitric oxide production.^42^

The present study found no significant differences in mortality, revascularisation or MI rates among the three groups during follow-up. The NT group exhibited the lowest rate of graft failure, yet it did not demonstrate better clinical outcomes. The possible reasons are as follows: First, the follow-up duration might not have been long enough. Although some patients experienced graft failure, their clinical symptoms were not pronounced, and they had not yet required revascularisation. Additionally, the clinical progression of symptoms is multifactorial and not solely influenced by graft failure.^19^ This conclusion was consistently reached in studies comparing the NT and OVH groups, but prior studies investigating the impact of EVH on clinical outcomes compared with OVH have yielded inconsistent findings. For instance, a study by Lopes *et al* suggested that EVH was associated with higher rates of mortality, revascularisation and MI compared with OVH (20.2% vs 17.4%, HR 1.22, p=0.04).^7^ Besides, the EXCEL study found no significant difference between EVH and OVH regarding the composite endpoint of death, stroke or MI at 5 years but a higher 5-year revascularisation rate in the EVH group (11.5% vs 6.7%, p=0.047).^35^ Conversely, some RCTs^43^ and multiple large observational studies^44 45^ have suggested that the clinical outcomes of EVH are not inferior to those of OVH.

Our study demonstrated that the ranking of free from leg wound infection was EVH>OVH >NT, which was consistent with previous meta-analysis.^19 20 46^ Current guidelines recommend EVH by experienced operators to reduce leg wound complications (Class IIa, level of evidence A).^34^ However, the high leg wound complications of NT may have potential implications for survival outcomes and limit its widespread adoption. Given that the NT group fails to achieve significantly better clinical outcomes and the increased risk of leg wound infection, we should cautiously assess the clinical significance of NT in improving graft patency rate.

This meta-analysis included only eligible RCTs to minimise potential selection bias, particularly incorporating large-sample RCTs from recent years, which strengthened the reliability of the conclusions. However, several limitations should be acknowledged. First, a pairwise meta-analysis between the EVH and NT groups was not performed, as only one study provided this comparison. Second, annualised rates of mortality, revascularisation and MI were not reported, and follow-up durations varied across different RCTs. However, since follow-up times were comparable within each RCT, significant bias was minimised. Third, as this was a study level meta-analysis, it was infeasible to integrate the mortality, revascularisation and MI into a single composite outcome for analysis. Fourth, this network meta-analysis included studies spanning two decades, meaning the findings may have been influenced by advancements in surgical techniques.

## Conclusion

This network meta-analysis demonstrated that NT was associated with lower graft failure and graft occlusion rates, yet it had higher leg wound infection rates, while follow-up mortality, revascularisation and MI rates remained comparable among the three techniques. These findings require cautious interpretation, and it is important to balance harvest site complications and the desirability of long-term graft patency.

## Supplementary material

10.1136/openhrt-2025-003728 online supplemental file 1

## Data Availability

Data are available in a public, open access repository.

## References

[1] Taggart DP (2018). How I deploy arterial grafts. Ann Cardiothorac Surg.

[2] Lawton JS, Tamis-Holland JE, Bangalore S (2022). 2021 ACC/AHA/SCAI Guideline for Coronary Artery Revascularization: Executive Summary: A Report of the American College of Cardiology/American Heart Association Joint Committee on Clinical Practice Guidelines. Circulation.

[3] Ruel M, Chikwe J (2024). Coronary Artery Bypass Grafting: Past and Future. Circulation.

[4] Kopjar T, Dashwood MR (2016). Endoscopic Versus “No-Touch” Saphenous Vein Harvesting for Coronary Artery Bypass Grafting: A Trade-Off Between Wound Healing and Graft Patency. Angiol Open Access.

[5] Deb S, Cohen EA, Singh SK (2012). Radial artery and saphenous vein patency more than 5 years after coronary artery bypass surgery: results from RAPS (Radial Artery Patency Study). J Am Coll Cardiol.

[6] Kopjar T, Dashwood MR (2022). Towards Endoscopic No-Touch Saphenous Vein Graft Harvesting in Coronary Bypass Surgery. Braz J Cardiovasc Surg.

[7] Lopes RD, Hafley GE, Allen KB (2009). Endoscopic versus open vein-graft harvesting in coronary-artery bypass surgery. N Engl J Med.

[8] Dashwood MR, Tsui JC (2013). “No-touch” saphenous vein harvesting improves graft performance in patients undergoing coronary artery bypass surgery: a journey from bedside to bench. Vascul Pharmacol.

[9] Souza D (1996). A new no-touch preparation technique. Technical notes. *Scand J Thorac Cardiovasc Surg*.

[10] Souza DSR, Dashwood MR, Tsui JCS (2002). Improved patency in vein grafts harvested with surrounding tissue: results of a randomized study using three harvesting techniques. Ann Thorac Surg.

[11] Peng Z, Zhao R, Liu Z (2022). The No-Touch Saphenous Vein Harvesting Improves Graft Patency After Off-Pump Coronary Artery Bypass Surgery: A Propensity-Matched Analysis. Braz J Cardiovasc Surg.

[12] Deb S, Singh SK, de Souza D (2019). SUPERIOR SVG: no touch saphenous harvesting to improve patency following coronary bypass grafting (a multi-Centre randomized control trial, NCT01047449). J Cardiothorac Surg.

[13] Deng MX, Lee GS, Vervoort D (2024). No-touch saphenous vein: current understanding of the conduit ‘less handled’. Curr Opin Cardiol.

[14] Tennyson C, Young CP, Scarci M (2010). Is it safe to perform endoscopic vein harvest? Interact Cardiovasc Thorac Surg.

[15] Soetisna TW, Thamrin AMH, Ilham MBS (2025). No-touch technique for saphenous vein graft harvesting in coronary artery bypass surgery safely improves graft patency: a meta-analysis of randomized controlled trials. *Indian J Thorac Cardiovasc Surg*.

[16] Kodia K, Patel S, Weber MP (2018). Graft patency after open versus endoscopic saphenous vein harvest in coronary artery bypass grafting surgery: a systematic review and meta-analysis. Ann Cardiothorac Surg.

[17] Sastry P, Rivinius R, Harvey R (2013). The influence of endoscopic vein harvesting on outcomes after coronary bypass grafting: a meta-analysis of 267,525 patients. Eur J Cardiothorac Surg.

[18] Deppe A-C, Liakopoulos OJ, Choi Y-H (2013). Endoscopic vein harvesting for coronary artery bypass grafting: a systematic review with meta-analysis of 27,789 patients. J Surg Res.

[19] Ali MA, Alam U, Khattak F (2025). Comparative efficacy and safety of no-touch versus conventional vein harvesting techniques in coronary artery bypass grafting: a systematic review and meta-analysis. Open Heart.

[20] Vuong NL, Elfaituri MK, Eldoadoa M (2022). Saphenous vein harvesting techniques for coronary artery bypass grafting: a systematic review and meta-analysis. Coron Artery Dis.

[21] Page MJ, McKenzie JE, Bossuyt PM (2021). The PRISMA 2020 statement: An updated guideline for reporting systematic reviews. Int J Surg.

[22] Shea BJ, Reeves BC, Wells G (2017). AMSTAR 2: a critical appraisal tool for systematic reviews that include randomised or non-randomised studies of healthcare interventions, or both. BMJ.

[23] Sterne JAC, Savović J, Page MJ (2019). RoB 2: a revised tool for assessing risk of bias in randomised trials. BMJ.

[24] Shim S, Yoon B-H, Shin I-S (2017). Network meta-analysis: application and practice using Stata. Epidemiol Health.

[25] Bonde P, Graham ANJ, MacGowan SW (2004). Endoscopic vein harvest: advantages and limitations. Ann Thorac Surg.

[26] Mbuagbaw L, Rochwerg B, Jaeschke R (2017). Approaches to interpreting and choosing the best treatments in network meta-analyses. Syst Rev.

[27] Zhao DF, Edelman JJ, Seco M (2017). Coronary Artery Bypass Grafting With and Without Manipulation of the Ascending Aorta: A Network Meta-Analysis. J Am Coll Cardiol.

[28] Balshem H, Helfand M, Schünemann HJ (2011). GRADE guidelines: 3. Rating the quality of evidence. J Clin Epidemiol.

[29] Tian M, Wang X, Sun H (2021). No-Touch Versus Conventional Vein Harvesting Techniques at 12 Months After Coronary Artery Bypass Grafting Surgery: Multicenter Randomized, Controlled Trial. Circulation.

[30] Goldman S, Zadina K, Moritz T (2004). Long-term patency of saphenous vein and left internal mammary artery grafts after coronary artery bypass surgery: results from a Department of Veterans Affairs Cooperative Study. J Am Coll Cardiol.

[31] Samano N, Geijer H, Liden M (2015). The no-touch saphenous vein for coronary artery bypass grafting maintains a patency, after 16 years, comparable to the left internal thoracic artery: A randomized trial. J Thorac Cardiovasc Surg.

[32] Xenogiannis I, Zenati M, Bhatt DL (2021). Saphenous Vein Graft Failure: From Pathophysiology to Prevention and Treatment Strategies. Circulation.

[33] Dreifaldt M, Mannion JD, Geijer H (2021). The no-touch saphenous vein is an excellent alternative conduit to the radial artery 8 years after coronary artery bypass grafting: A randomized trial. J Thorac Cardiovasc Surg.

[34] Neumann FJ, Sousa-Uva M, Ahlsson A (2018). ESC/EACTS Guidelines on myocardial revascularization. Eur Heart J.

[35] Jarrett CM, Pelletier M, Abu-Omar Y (2023). Endoscopic vs Open Vein Harvest in Drug-Eluting Stents or Bypass Surgery for Left Main Disease Trial. Ann Thorac Surg.

[36] Andreasen JJ, Vadmann H, Oddershede L (2015). Decreased patency rates following endoscopic vein harvest in coronary artery bypass surgery. Scand Cardiovasc J.

[37] Yun KL, Wu Y, Aharonian V (2005). Randomized trial of endoscopic versus open vein harvest for coronary artery bypass grafting: six-month patency rates. J Thorac Cardiovasc Surg.

[38] Perrault LP, Jeanmart H, Bilodeau L (2004). Early quantitative coronary angiography of saphenous vein grafts for coronary artery bypass grafting harvested by means of open versus endoscopic saphenectomy: a prospective randomized trial. J Thorac Cardiovasc Surg.

[39] Yokoyama Y, Shimamura J, Takagi H (2021). Harvesting techniques of the saphenous vein graft for coronary artery bypass: Insights from a network meta‐analysis. J Card Surg.

[40] Saito T, Kurazumi H, Suzuki R (2022). Perivascular Adipose Tissue Is a Major Source of Nitric Oxide in Saphenous Vein Grafts Harvested via the No-Touch Technique. J Am Heart Assoc.

[41] Fabricius AM, Diegeler A, Gerber W (2000). Functional and morphologic assessment of saphenous veins harvested with minimally invasive techniques using a modified laryngoscope. Heart Surg Forum.

[42] Rousou LJ, Taylor KB, Lu X-G (2009). Saphenous vein conduits harvested by endoscopic technique exhibit structural and functional damage. Ann Thorac Surg.

[43] Zenati MA, Bhatt DL, Stock EM (2021). Intermediate-Term Outcomes of Endoscopic or Open Vein Harvesting for Coronary Artery Bypass Grafting: The REGROUP Randomized Clinical Trial. JAMA Netw Open.

[44] Dacey LJ, Braxton JH, Kramer RS (2011). Long-term outcomes of endoscopic vein harvesting after coronary artery bypass grafting. Circulation.

[45] Williams JB, Peterson ED, Brennan JM (2012). Association between endoscopic vs open vein-graft harvesting and mortality, wound complications, and cardiovascular events in patients undergoing CABG surgery. JAMA.

[46] Cheng D, Allen K, Cohn W (2005). Endoscopic vascular harvest in coronary artery bypass grafting surgery: a meta-analysis of randomized trials and controlled trials. Innovations (Phila).

[47] Thelin S, Modrau IS, Duvernoy O (2025). No-touch vein grafts in coronary artery bypass surgery: a registry-based randomized clinical trial. Eur Heart J.

[48] Zenati MA, Bhatt DL, Stock E (2020). Long-term Outcomes After Endoscopic or Open Vein Harvest for Coronary Artery Bypass: REGROUP Trial. Circulation.

[49] Zenati MA, Bhatt DL, Bakaeen FG (2019). Randomized Trial of Endoscopic or Open Vein-Graft Harvesting for Coronary-Artery Bypass. N Engl J Med.

[50] Zenati MA, Bhatt DL, Bakaeen FG (2018). Endoscopic vein harvest for coronary bypass surgery in a randomized multicenter trial with long-term follow-up. Circulation.

[51] Zenati MA, Shroyer AL, Collins JF (2011). Impact of endoscopic versus open saphenous vein harvest technique on late coronary artery bypass grafting patient outcomes in the ROOBY (Randomized On/Off Bypass) Trial. *J Thorac Cardiovasc Surg*.

[52] Hou X, Zhang K, Liu T (2021). The expansion of no-touch harvesting sequential vein graft after off-pump coronary artery bypass grafting. J Card Surg.

[53] Pettersen Ø, Haram PM, Winnerkvist A (2017). Pedicled Vein Grafts in Coronary Surgery: Perioperative Data From a Randomized Trial. Ann Thorac Surg.

[54] Pettersen Ø, Wiseth R, Hegbom K (2016). Pedicled Vein Grafts in Coronary Surgery Exhibit Reduced Intimal Hyperplasia at 6 Months. J Am Coll Cardiol.

[55] Andreasen JJ, Nekrasas V, Dethlefsen C (2008). Endoscopic vs open saphenous vein harvest for coronary artery bypass grafting: a prospective randomized trial. Eur J Cardiothorac Surg.

[56] Souza DSR, Johansson B, Bojö L (2006). Harvesting the saphenous vein with surrounding tissue for CABG provides long-term graft patency comparable to the left internal thoracic artery: results of a randomized longitudinal trial. J Thorac Cardiovasc Surg.

[57] Johansson BL, Souza DS, Bodin L (2009). No touch vein harvesting technique for CABG improves the long-term clinical outcome. Scand Cardiovasc J.

[58] Chernyavskiy A, Volkov A, Lavrenyuk O (2015). Comparative results of endoscopic and open methods of vein harvesting for coronary artery bypass grafting: a prospective randomized parallel-group trial. J Cardiothorac Surg.

[59] Brat R, Horacek J, Sieja J (2013). Endoscopic vs open saphenous vein harvest for coronary artery bypass grafting: a leg-related morbidity and histological comparison. Biomed Pap Med Fac Univ Palacky Olomouc Czech Repub.

[60] Krishnamoorthy B, Critchley WR, Glover AT (2012). A randomized study comparing three groups of vein harvesting methods for coronary artery bypass grafting: endoscopic harvest versus standard bridging and open techniques. Interact Cardiovasc Thorac Surg.

[61] Wang H, Wu H, Jiang H (2011). Initial Experience with Endoscopic Saphenous Vein Harvesting for Coronary Artery Bypass Graft ing in Chinese Patients. Heart Surg Forum.

[62] Au WKT, Chiu SW, Sun MP (2008). Improved leg wound healing with endoscopic saphenous vein harvest in coronary artery bypass graft surgery: a prospective randomized study in Asian population. J Card Surg.

[63] Bonde P, Graham A, MacGowan S (2002). Endoscopic vein harvest: early results of a prospective trial with open vein harvest. Heart Surg Forum.

[64] Kiaii B, Moon BC, Massel D (2002). A prospective randomized trial of endoscopic versus conventional harvesting of the saphenous vein in coronary artery bypass surgery. J Thorac Cardiovasc Surg.

[65] Schurr UP, Lachat ML, Reuthebuch O (2002). Endoscopic saphenous vein harvesting for CABG -- a randomized, prospective trial. Thorac Cardiovasc Surg.

[66] Fabricius AM, Diegeler A, Doll N (2000). Minimally invasive saphenous vein harvesting techniques: morphology and postoperative outcome. Ann Thorac Surg.

[67] Cisowski M, Wites M, Gerber W (2000). Minimally invasive saphenous vein harvesting for coronary artery bypass grafting--comparison of three less invasive methods. Med Sci Monit.

[68] Carpino PA, Khabbaz KR, Bojar RM (2000). Clinical benefits of endoscopic vein harvesting in patients with risk factors for saphenectomy wound infections undergoing coronary artery bypass grafting. J Thorac Cardiovasc Surg.

[69] Puskas JD, Wright CE, Miller PK (1999). A randomized trial of endoscopic versus open saphenous vein harvest in coronary bypass surgery. Ann Thorac Surg.

[70] Hayward TZ, Hey LA, Newman LL (1999). Endoscopic versus open saphenous vein harvest: the effect on postoperative outcomes. Ann Thorac Surg.

[71] Folliguet TA, Le Bret E, Moneta A (1998). Endoscopic saphenous vein harvesting versus “open” technique. A prospective study. Eur J Cardiothorac Surg.

[72] Allen KB, Griffith GL, Heimansohn DA (1998). Endoscopic versus traditional saphenous vein harvesting: a prospective, randomized trial. Ann Thorac Surg.

[73] Morris RJ, Butler MT, Samuels LE (1998). Minimally invasive saphenous vein harvesting. Ann Thorac Surg.

