# Supplementary material for: Techniques for harvesting the saphenous vein in coronary artery bypass grafting: a network systematic review and meta-analysis
Source: Open Heart. 2025 Dec 18;12(2):e003728. doi: 10.1136/openhrt-2025-003728 (PMC12716500; doi:10.1136/openhrt-2025-003728)
Supplement: online supplemental file 1 [file openhrt-12-2-s001.docx]

**Techniques for Harvesting the Saphenous Vein in Coronary Artery Bypass Grafting: A Network Systematic Review and Meta-Analysis**

**Supplemental data**

**Table S1. Search strategy for databases**

| **Search keywords** | **Search database** |
| --- | --- |
| (saphenous vein) AND (harvest OR graft ) AND (bypass OR CABG) AND (randomized OR randomly OR randomization OR (clinical trial)) | PubMed, Cochrane Central Registry, Web of science |

**References:**

[1] Allen K B, Griffith G L, Heimansohn D A, et al. Endoscopic versus traditional saphenous vein harvesting: a prospective, randomized trial[J]. Ann Thorac Surg, 1998, 66(1): 26-31; discussion 31-2.

[2] Andreasen J J, Vadmann H, Oddershede L, et al. Decreased patency rates following endoscopic vein harvest in coronary artery bypass surgery[J]. Scand Cardiovasc J, 2015, 49(5): 286-92.

[3] Au W K, Chiu S W, Sun M P, et al. Improved leg wound healing with endoscopic saphenous vein harvest in coronary artery bypass graft surgery: a prospective randomized study in Asian population[J]. J Card Surg, 2008, 23(6): 633-7.

[4] Brat R, Horacek J, Sieja J. Endoscopic vs open saphenous vein harvest for coronary artery bypass grafting: a leg-related morbidity and histological comparison[J]. Biomed Pap Med Fac Univ Palacky Olomouc Czech Repub, 2013, 157(1): 70-4.

[5] Bonde P, Graham A N, Macgowan S W. Endoscopic vein harvest: advantages and limitations[J]. Ann Thorac Surg, 2004, 77(6): 2076-82.

[6] Carpino P A, Khabbaz K R, Bojar R M, et al. Clinical benefits of endoscopic vein harvesting in patients with risk factors for saphenectomy wound infections undergoing coronary artery bypass grafting[J]. J Thorac Cardiovasc Surg, 2000, 119(1): 69-75.

[7] Chernyavskiy A, Volkov A, Lavrenyuk O, et al. Comparative results of endoscopic and open methods of vein harvesting for coronary artery bypass grafting: a prospective randomized parallel-group trial[J]. J Cardiothorac Surg, 2015, 10: 163.

[8] Cisowski M, Wites M, Gerber W, et al. Minimally invasive saphenous vein harvesting for coronary artery bypass grafting--comparison of three less invasive methods[J]. Med Sci Monit, 2000, 6(4): 735-9.

[9] Deb S, Singh S K, De Souza D, et al. SUPERIOR SVG: no touch saphenous harvesting to improve patency following coronary bypass grafting (a multi-Centre randomized control trial, NCT01047449)[J]. J Cardiothorac Surg, 2019, 14(1): 85.

[10] Fabricius A M, Diegeler A, Doll N, et al. Minimally invasive saphenous vein harvesting techniques: morphology and postoperative outcome[J]. Ann Thorac Surg, 2000, 70(2): 473-8.

[11] Folliguet T A, Le Bret E, Moneta A, et al. Endoscopic saphenous vein harvesting versus 'open' technique. A prospective study[J]. Eur J Cardiothorac Surg, 1998, 13(6): 662-6.

[12] Hayward T Z, 3rd, Hey L A, Newman L L, et al. Endoscopic versus open saphenous vein harvest: the effect on postoperative outcomes[J]. Ann Thorac Surg, 1999, 68(6): 2107-10; discussion 2110-1.

[13] Hou X, Zhang K, Liu T, et al. The expansion of no-touch harvesting sequential vein graft after off-pump coronary artery bypass grafting[J]. J Card Surg, 2021, 36(7): 2381-2388.

[14] Kiaii B, Moon B C, Massel D, et al. A prospective randomized trial of endoscopic versus open harvesting of the saphenous vein in coronary artery bypass surgery[J]. J Thorac Cardiovasc Surg, 2002, 123(2): 204-12.

[15] Krishnamoorthy B, Critchley W R, Glover A T, et al. A randomized study comparing three groups of vein harvesting methods for coronary artery bypass grafting: endoscopic harvest versus standard bridging and open techniques[J]. Interact Cardiovasc Thorac Surg, 2012, 15(2): 224-8.

[16] Morris R J, Butler M T, Samuels L E. Minimally invasive saphenous vein harvesting[J]. Ann Thorac Surg, 1998, 66(3): 1026-8.

[17] Perrault L P, Jeanmart H, Bilodeau L, et al. Early quantitative coronary angiography of saphenous vein grafts for coronary artery bypass grafting harvested by means of open versus endoscopic saphenectomy: a prospective randomized trial[J]. J Thorac Cardiovasc Surg, 2004, 127(5): 1402-7.

[18] Pettersen Ø, Haram P M, Winnerkvist A, et al. Pedicled Vein Grafts in Coronary Surgery: Perioperative Data From a Randomized Trial[J]. Ann Thorac Surg, 2017, 104(4): 1313-1317.

[19] Puskas J D, Wright C E, Miller P K, et al. A randomized trial of endoscopic versus open saphenous vein harvest in coronary bypass surgery[J]. Ann Thorac Surg, 1999, 68(4): 1509-12.

[20] Samano N, Geijer H, Liden M, et al. The no-touch saphenous vein for coronary artery bypass grafting maintains a patency, after 16 years, comparable to the left internal thoracic artery: A randomized trial[J]. J Thorac Cardiovasc Surg, 2015, 150(4): 880-8.

[21] Schurr U P, Lachat M L, Reuthebuch O, et al. Endoscopic saphenous vein harvesting for CABG -- a randomized, prospective trial[J]. Thorac Cardiovasc Surg, 2002, 50(3): 160-3.

[22] Thelin S, Modrau I S, Duvernoy O, et al. No-touch vein grafts in coronary artery bypass surgery: a registry-based randomized clinical trial[J]. Eur Heart J, 2025, 46(18): 1720-1729.

[23] Tian M, Wang X, Sun H, et al. No-Touch Versus open Vein Harvesting Techniques at 12 Months After Coronary Artery Bypass Grafting Surgery Multicenter Randomized, Controlled Trial[J]. CIRCULATION, 2021, 144(14): 1120-1129.

[24] Wang H, Wu H, Jiang H, et al. Initial experience with endoscopic saphenous vein harvesting for coronary artery bypass grafting in Chinese patients[J]. Heart Surg Forum, 2011, 14(5): E291-6.

[25] Yun K L, Wu Y, Aharonian V, et al. Randomized trial of endoscopic versus open vein harvest for coronary artery bypass grafting: six-month patency rates[J]. J Thorac Cardiovasc Surg, 2005, 129(3): 496-503.

[26] Zenati M A, Bhatt D L, Stock E M, et al. Intermediate-Term Outcomes of Endoscopic or Open Vein Harvesting for Coronary Artery Bypass Grafting: The REGROUP Randomized Clinical Trial[J]. JAMA Netw Open, 2021, 4(3): e211439.

**Table S2-1. GRADE profile for graft failure network meta-analysis.**

| **Certainty assessment** | | | | | | | **№ of patients** | | **Effect** | | **Certainty** | **Importance** |
| --- | --- | --- | --- | --- | --- | --- | --- | --- | --- | --- | --- | --- |
| **№ of studies** | **Study design** | **Risk of bias** | **Inconsistency** | **Indirectness** | **Imprecision** | **Other considerations** | **Intervention** | **Control** | **Relative**  **(95% CI)** | **Absolute**  **(95% CI)** |  |  |
| **OVH vs. EVH** | | | | | | | | | | | | |
| 4 | randomised trials | not serious | not serious | not serious | not serious | publication bias strongly suspected | 213/778  (27.4%) | 209/664 (31.5%) | **RR 0.85**  (0.58 to 1.25) | **47 fewer per 1,000**  (from 132 fewer to 79 more) | ⨁⨁◯◯  Low | NOT IMPORTANT |
| **OVH vs. NT** | | | | | | | | | | | | |
| 4 | randomised trials | not serious | not serious | not serious | not serious | none | 322/1664  (19.4%) | 238/1671  (14.2%) | **RR 1.37**  (1.16 to 1.62) | **53 more per 1,000**  (from 23 more to 88 more) | ⨁⨁⨁⨁  High | IMPORTANT |

**CI:** confidence interval; **OR:** odds ratio

**Table S2-2. GRADE profile for graft occlusion network meta-analysis.**

| **Certainty assessment** | | | | | | | **№ of patients** | | **Effect** | | **Certainty** | **Importance** |
| --- | --- | --- | --- | --- | --- | --- | --- | --- | --- | --- | --- | --- |
| **№ of studies** | **Study design** | **Risk of bias** | **Inconsistency** | **Indirectness** | **Imprecision** | **Other considerations** | **Intervention** | **Control** | **Relative**  **(95% CI)** | **Absolute**  **(95% CI)** |  |  |
| **OVH vs. EVH** | | | | | | | | | | | | |
| 2 | randomised trials | not serious | not serious | not serious | not  serious | none | 40/193(20.7%) | 35/202(17.3%) | **RR 1.19**  (0.79 to 1.80) | **33 more per 1,000**  (from 36 fewer to 139 more) | ⨁⨁⨁◯  Moderate | IMPORTANT |
| **OVH vs. NT** | | | | | | | | | | | | |
| 4 | randomised trials | not serious | not serious | not serious | not  serious | none | 174/1363  (12.8%) | 120/1365  (8.8%) | **RR 1.45**  (1.16 to 1.81) | **40 more per 1,000**  (from 14 more to 71 more) | ⨁⨁⨁⨁  High | IMPORTANT |

**CI:** confidence interval; **OR:** odds ratio

**Table S2-3. GRADE profile for mortality network meta-analysis.**

| **Certainty assessment** | | | | | | | **№ of patients** | | **Effect** | | **Certainty** | **Importance** |
| --- | --- | --- | --- | --- | --- | --- | --- | --- | --- | --- | --- | --- |
| **№ of studies** | **Study design** | **Risk of bias** | **Inconsistency** | **Indirectness** | **Imprecision** | **Other considerations** | **Intervention** | **Control** | **Relative**  **(95% CI)** | **Absolute**  **(95% CI)** |  |  |
| **OVH vs. EVH** | | | | | | | | | | | | |
| 8 | randomised trials | not serious | not serious | not serious | not  serious | none | 91/946(9.6%) | 79/937(8.4%) | **RR 1.16**  (0.88 to 1.55) | **13 more per 1,000**  (from 10 fewer to 46 more) | ⨁⨁⨁◯  Moderate | IMPORTANT |
| **OVH vs. NT** | | | | | | | | | | | | |
| 6 | randomised trials | not serious | not serious | not serious | not  serious | none | 72/2035  (3.5%) | 74/2057  (3.6%) | **RR 0.98**  (0.72 to 1.34) | **1 fewer per 1,000**  (from 10 fewer to 12 more) | ⨁⨁⨁◯  Moderate | IMPORTANT |

**CI:** confidence interval; **OR:** odds ratio

**Table S2-4. GRADE profile for revascularization network meta-analysis.**

| **Certainty assessment** | | | | | | | **№ of patients** | | **Effect** | | **Certainty** | **Importance** |
| --- | --- | --- | --- | --- | --- | --- | --- | --- | --- | --- | --- | --- |
| **№ of studies** | **Study design** | **Risk of bias** | **Inconsistency** | **Indirectness** | **Imprecision** | **Other considerations** | **Intervention** | **Control** | **Relative**  **(95% CI)** | **Absolute**  **(95% CI)** |  |  |
| **OVH vs. EVH** | | | | | | | | | | | | |
| 4 | randomised trials | not serious | not serious | not serious | serious | none | 57/809(7.0%) | 45/702(6.4%) | **RR 1.26**  (0.87 to 1.83) | **17 more per 1,000**  (from 8 fewer to 53 more) | ⨁⨁⨁◯  Moderate | IMPORTANT |
| **OVH vs. NT** | | | | | | | | | | | | |
| 4 | randomised trials | not serious | not serious | serious | serious | none | 67/1933  (3.5%) | 60/1955  (3.1%) | **RR 1.30**  (0.63 to 2.70) | **9 more per 1,000**  (from 11 fewer to 52 more) | ⨁⨁◯◯  low | NOT  IMPORTANT |

**CI:** confidence interval; **OR:** odds ratio

**Table S2-5. GRADE profile for myocardial infarction network meta-analysis.**

| **Certainty assessment** | | | | | | | **№ of patients** | | **Effect** | | **Certainty** | **Importance** |
| --- | --- | --- | --- | --- | --- | --- | --- | --- | --- | --- | --- | --- |
| **№ of studies** | **Study design** | **Risk of bias** | **Inconsistency** | **Indirectness** | **Imprecision** | **Other considerations** | **Intervention** | **Control** | **Relative**  **(95% CI)** | **Absolute**  **(95% CI)** |  |  |
| **OVH vs. EVH** | | | | | | | | | | | | |
| 6 | randomised trials | not serious | not serious | not serious | serious | none | 46/806(5.7%) | 40/803  (5.0%) | **RR 1.12**  (0.74 to 1.70) | **6 more per 1,000**  (from 13 fewer to 35 more) | ⨁⨁⨁◯  Moderate | IMPORTANT |
| **OVH vs. NT** | | | | | | | | | | | | |
| 4 | randomised trials | not serious | not serious | not  serious | serious | none | 46/1911  (2.4%) | 55/1955 (2.8%) | **RR 0.85**  (0.58 to 1.25) | **4 fewer per 1,000**  (from 12 fewer to 7 more) | ⨁⨁⨁◯  Moderate | IMPORTANT |

**CI:** confidence interval; **OR:** odds ratio

**Table S2-6. GRADE profile for leg wound infection network meta-analysis.**

| **Certainty assessment** | | | | | | | **№ of patients** | | **Effect** | | **Certainty** | **Importance** |
| --- | --- | --- | --- | --- | --- | --- | --- | --- | --- | --- | --- | --- |
| **№ of studies** | **Study design** | **Risk of bias** | **Inconsistency** | **Indirectness** | **Imprecision** | **Other considerations** | **Intervention** | **Control** | **Relative**  **(95% CI)** | **Absolute**  **(95% CI)** |  |  |
| **OVH vs. EVH** | | | | | | | | | | | | |
| 16 | randomised trials | not serious | not serious | not serious | not  serious | none | 95/813(11.7%) | 34/837(4.1%) | **RR 2.31**  (1.59 to 3.37) | **53 more per 1,000**  (from 24 more to 96 more) | ⨁⨁⨁⨁  High | IMPORTANT |
| **OVH vs. NT** | | | | | | | | | | | | |
| 3 | randomised trials | not serious | not serious | not serious | not  serious | none | 46/595  (7.7%) | 106/602  (17.6%) | **OR 0.44**  (0.32 to 0.61) | **99 fewer per 1,000**  (from 120 fewer to 69 fewer) | ⨁⨁⨁⨁  High | IMPORTANT |

**CI:** confidence interval; **OR:** odds ratio

**Supplemental Figures**


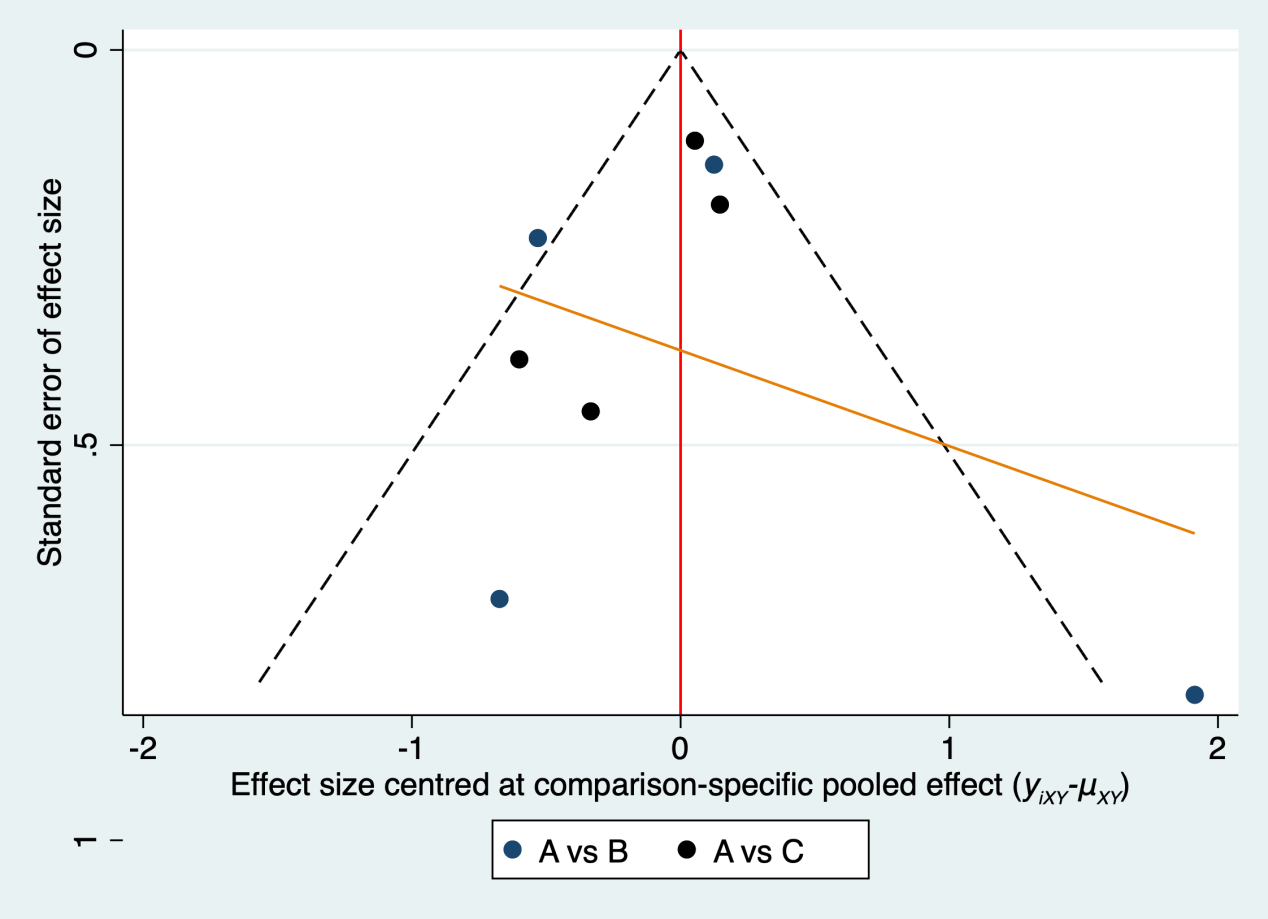


Figure S1-1. Comparison-adjusted funnel plot for graft failure.(A, OVH; B, EVH; C, NT)


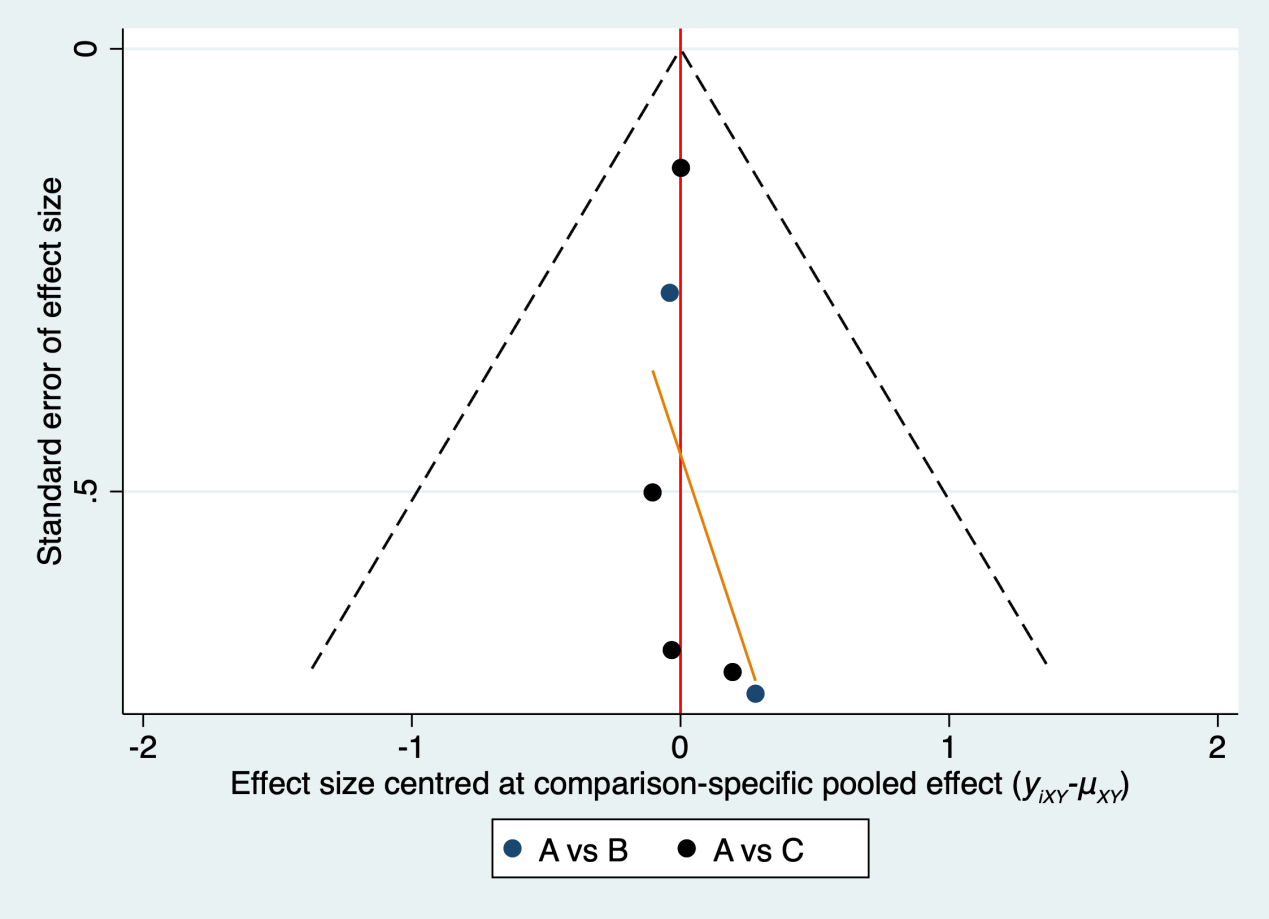


Figure S1-2. Comparison-adjusted funnel plot for graft occlusion.(A, OVH; B, EVH; C, NT)


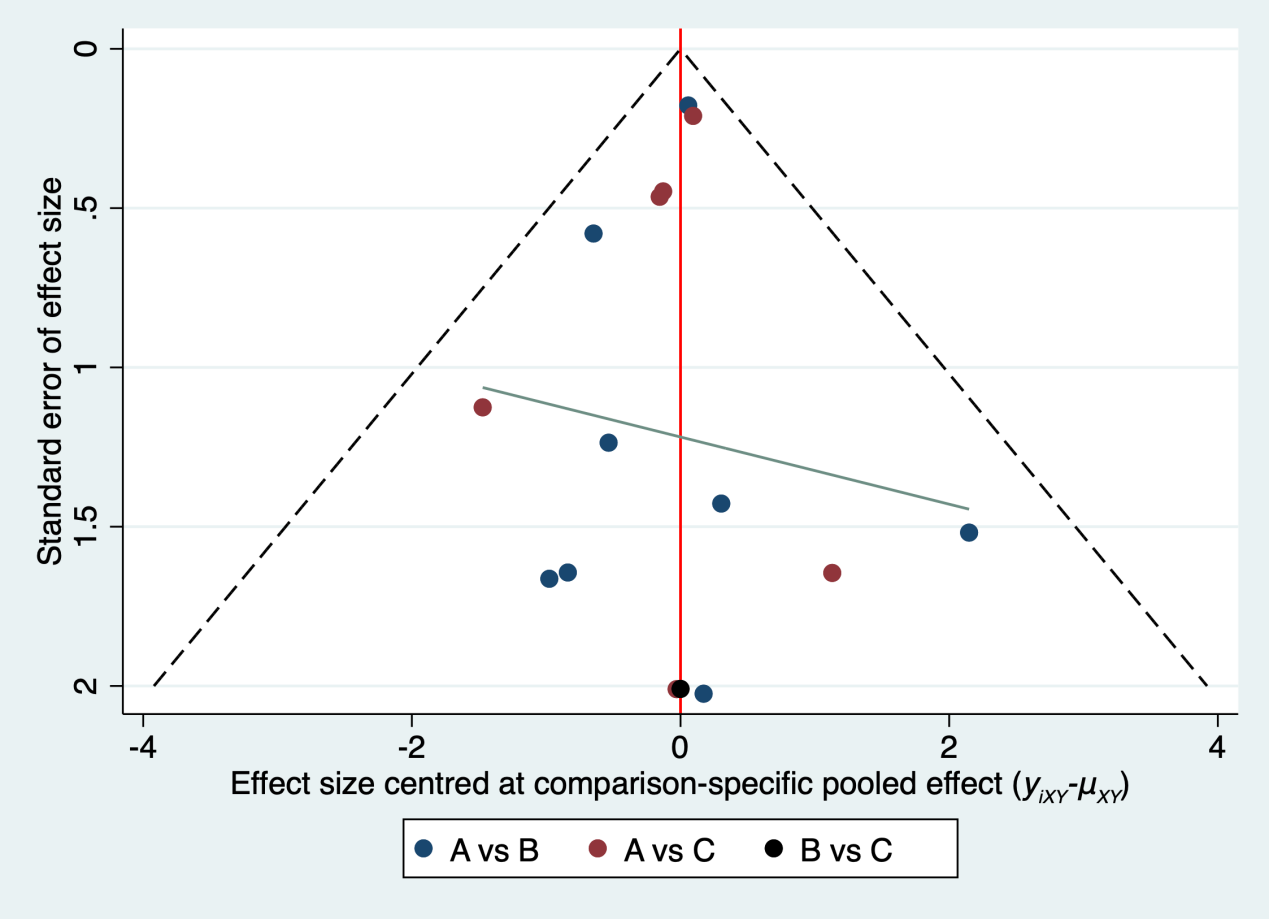


Figure S1-3. Comparison-adjusted funnel plot for mortality. (A, OVH; B, EVH; C, NT)


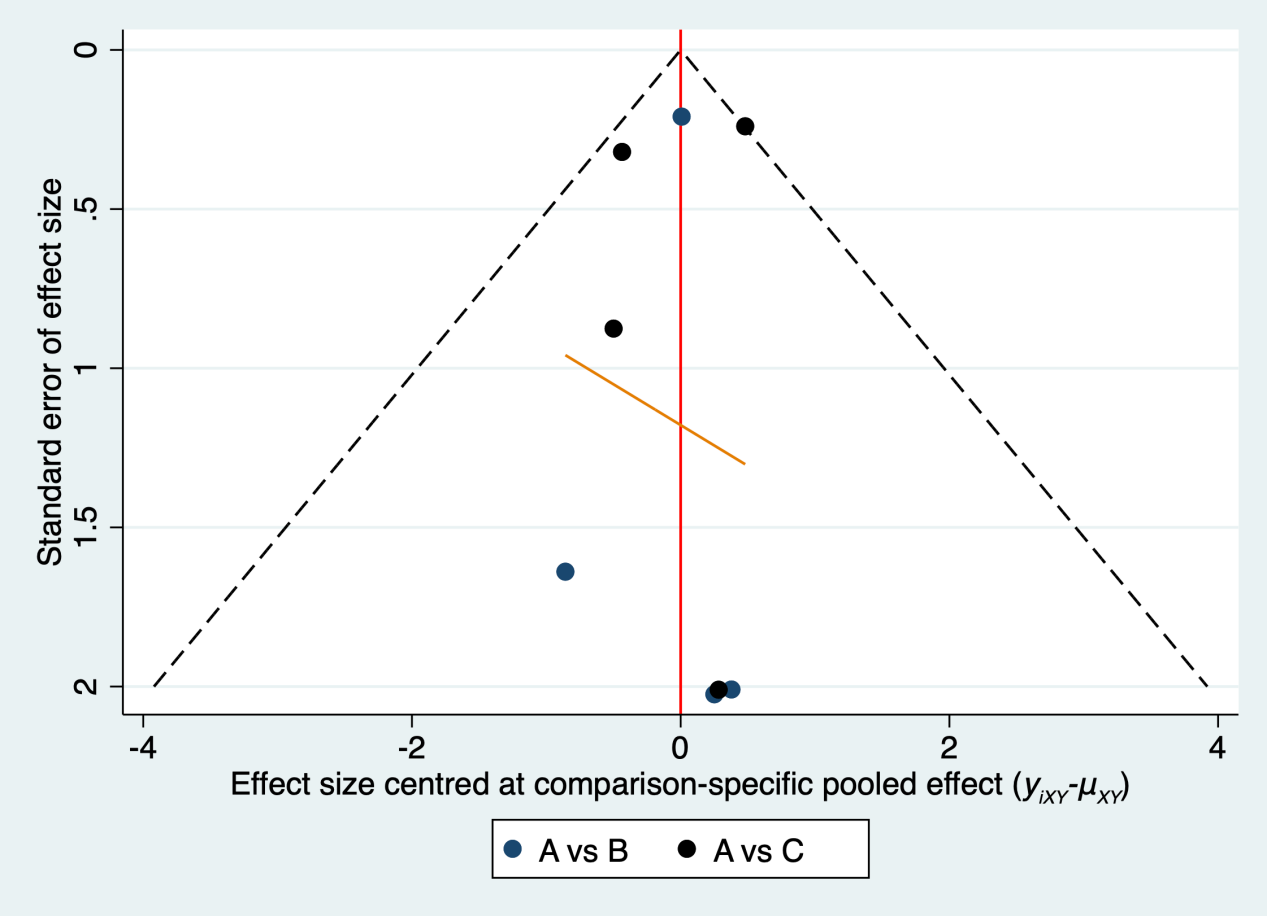


Figure S1-4. Comparison-adjusted funnel plot for revascularization.(A, OVH; B, EVH; C, NT)


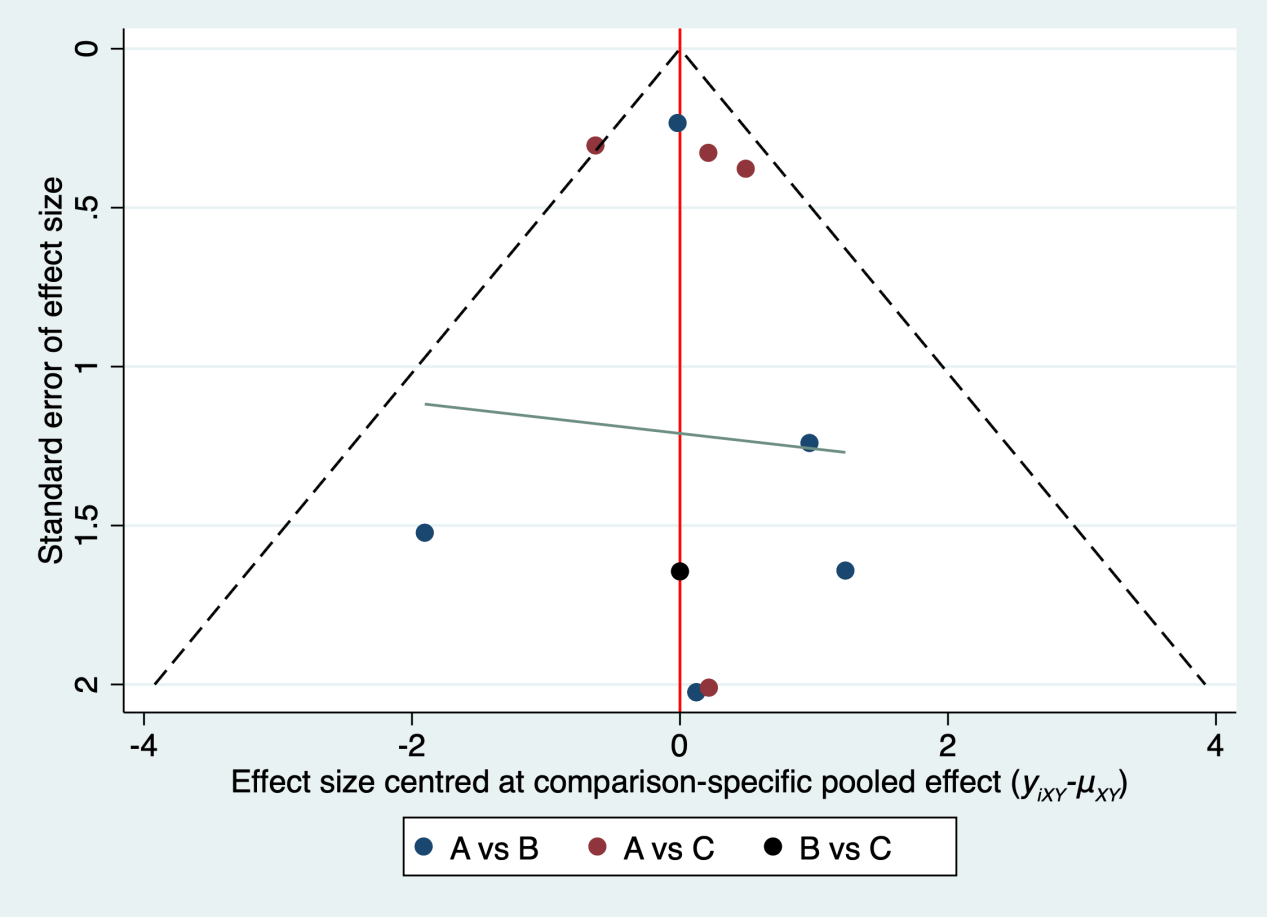


Figure S1-5. Comparison-adjusted funnel plot for myocardial infarction.(A, OVH; B, EVH; C, NT)


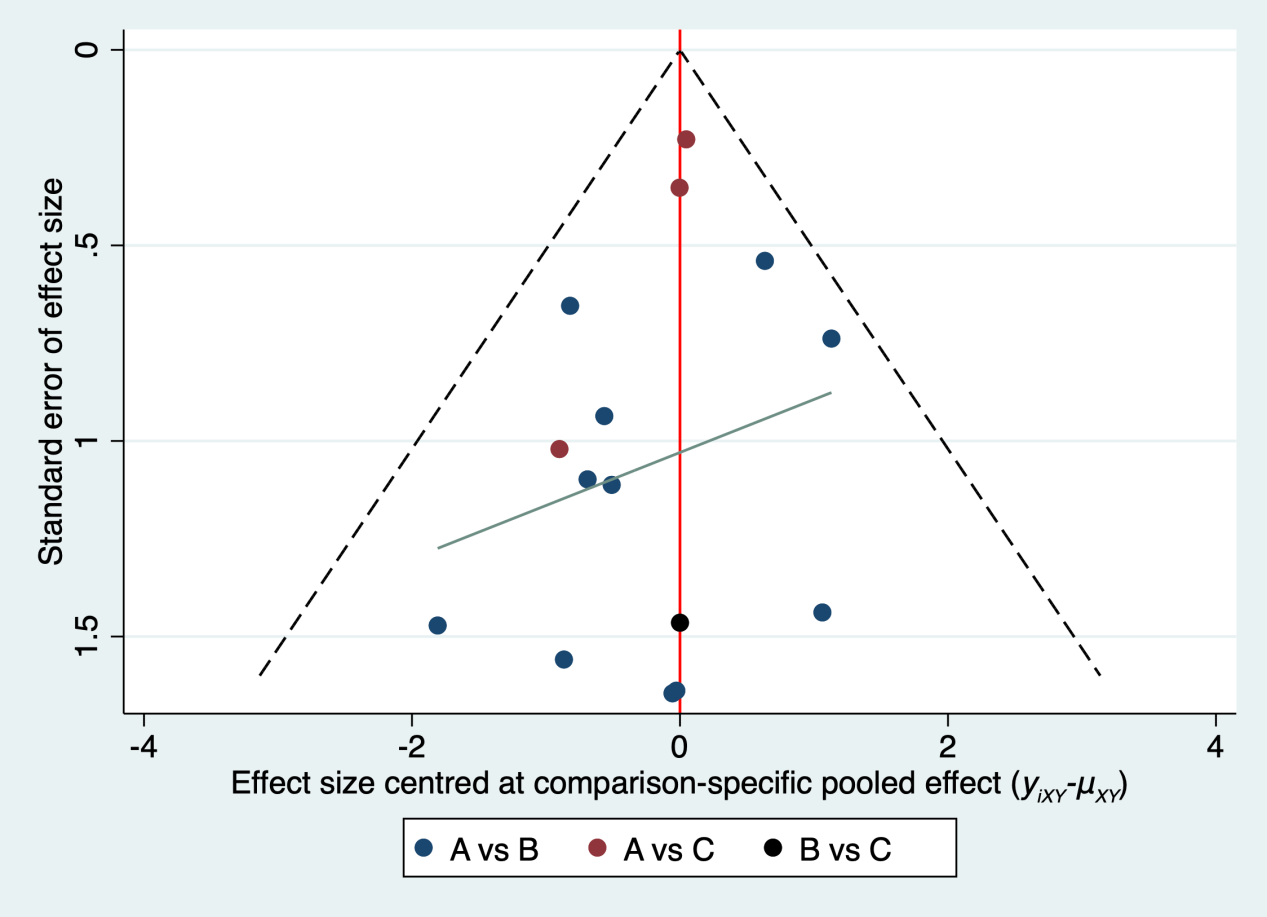


Figure S1-6. Comparison-adjusted funnel plot for leg wound infection.(A, OVH; B, EVH; C, NT)


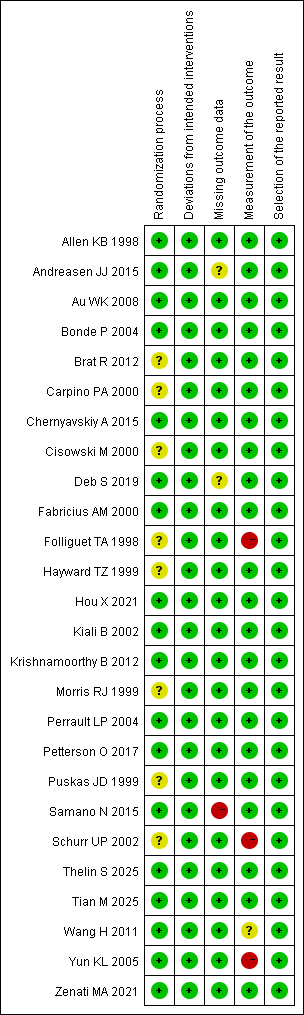


Figure S2-1. Risk of bias graph: the distribution of risk of bias judgments (Yes, Green; No, Red; Unclear, Yellow) for each study.


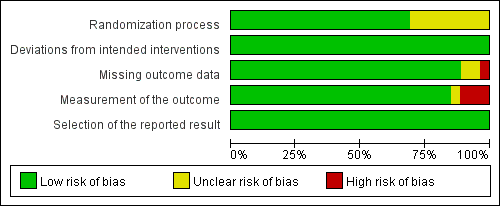


Figure S2-2. Risk of bias summary: a summary table of review authors’ risk of bias judgments for each study.


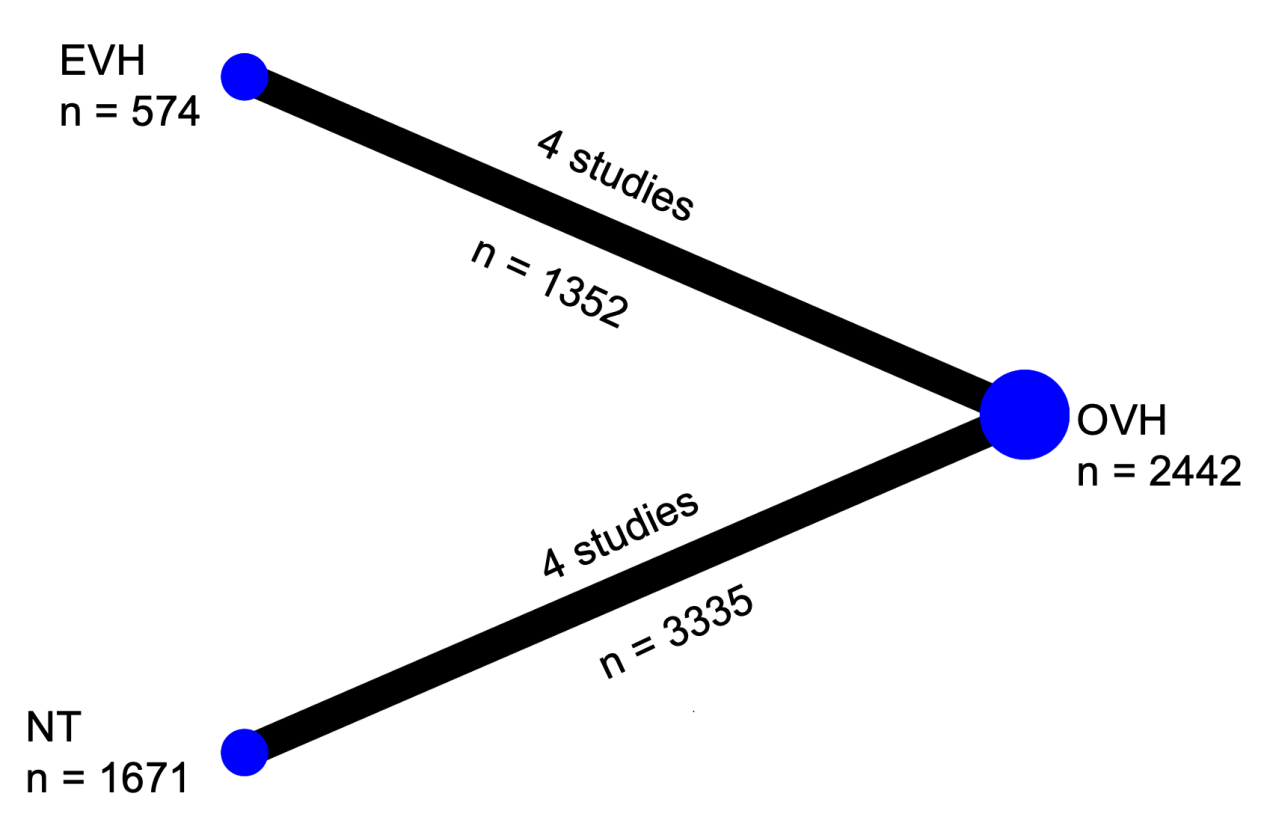


Figure S3-1. Network diagram of graft failure.


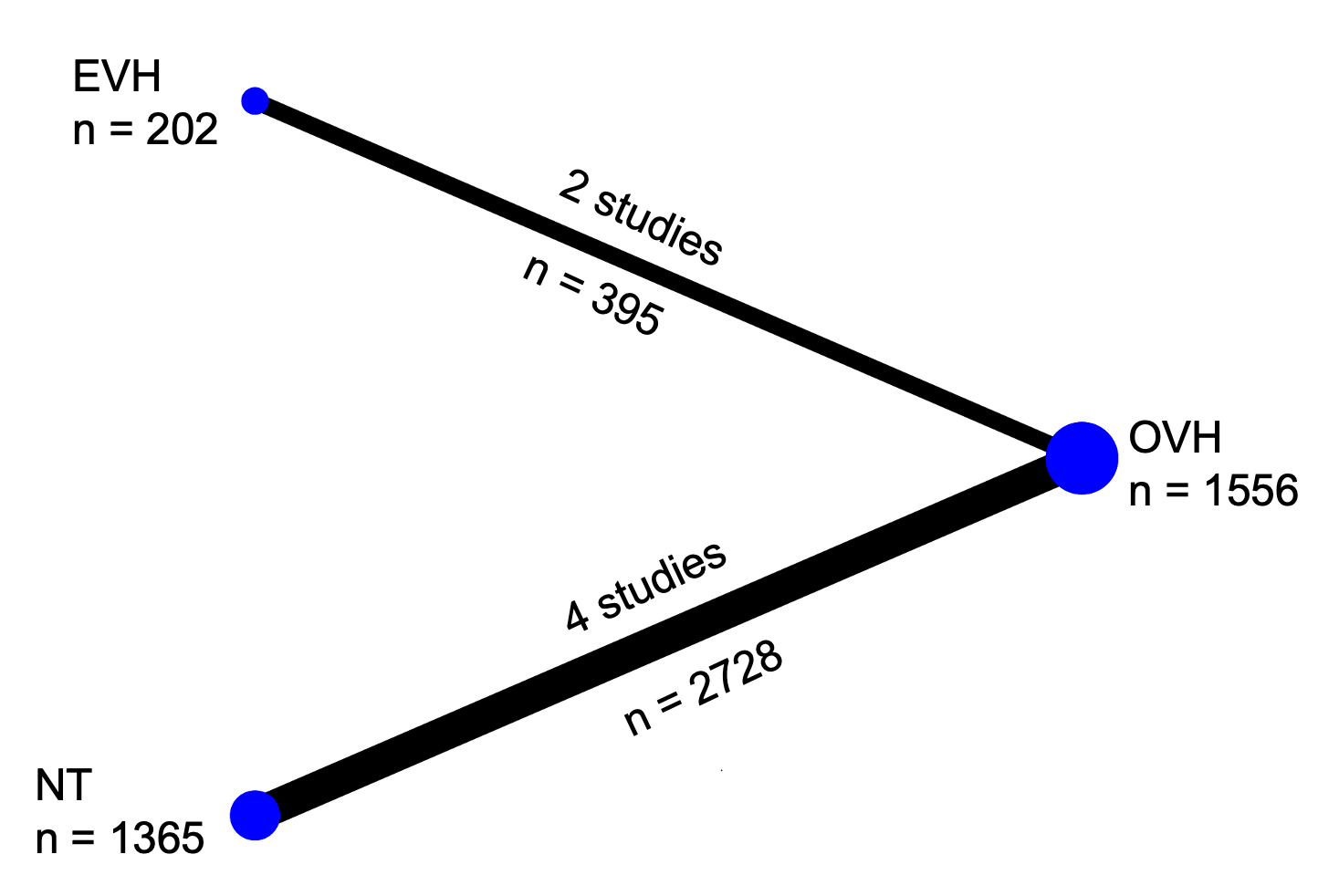


Figure S3-2. Network diagram of graft occlusion.


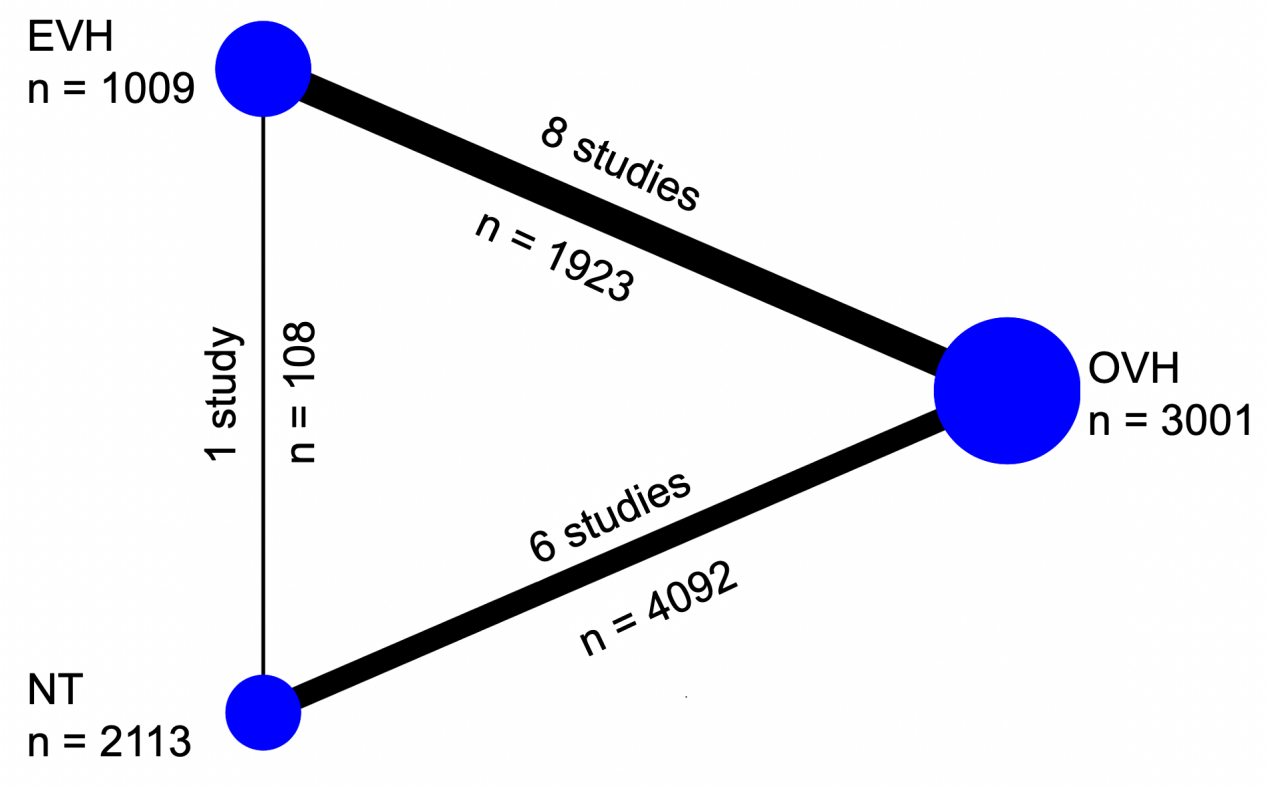


Figure S3-3. Network diagram of mortality.


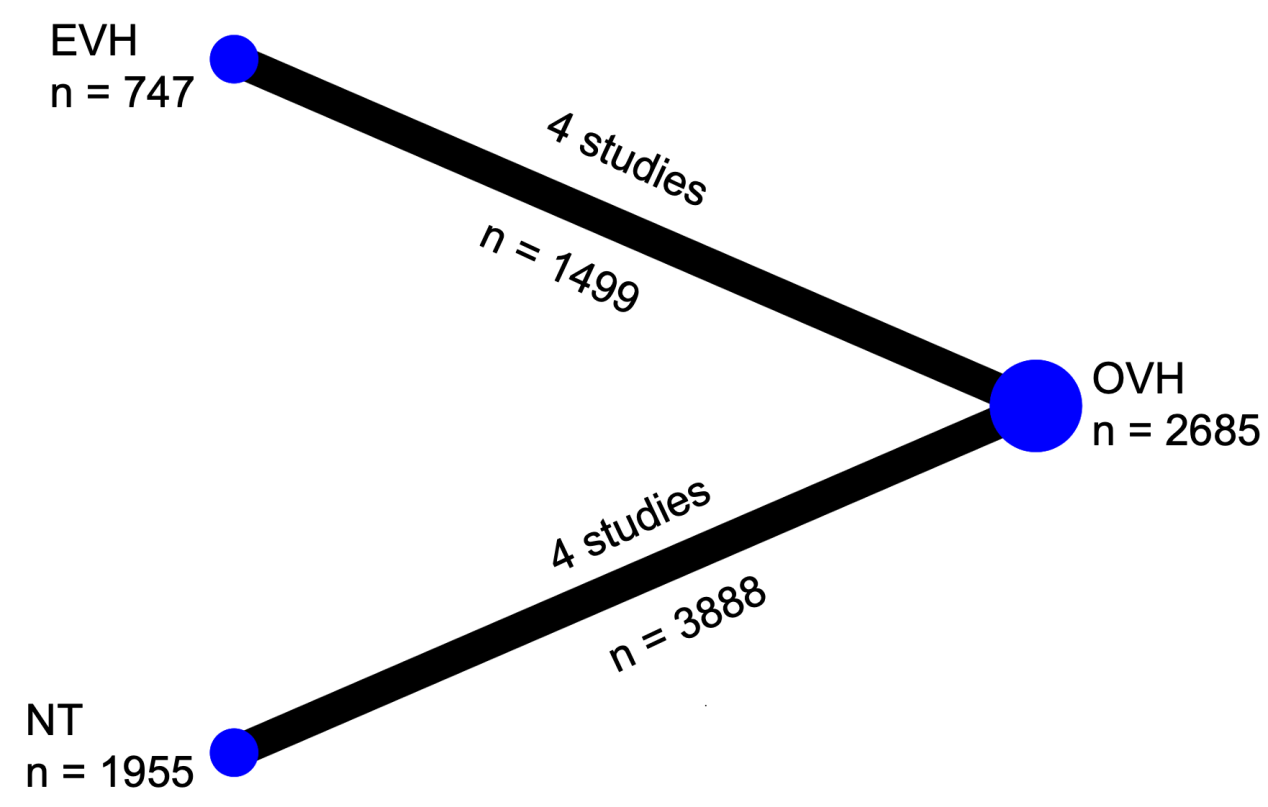


Figure S3-4. Network diagram of revascularization.


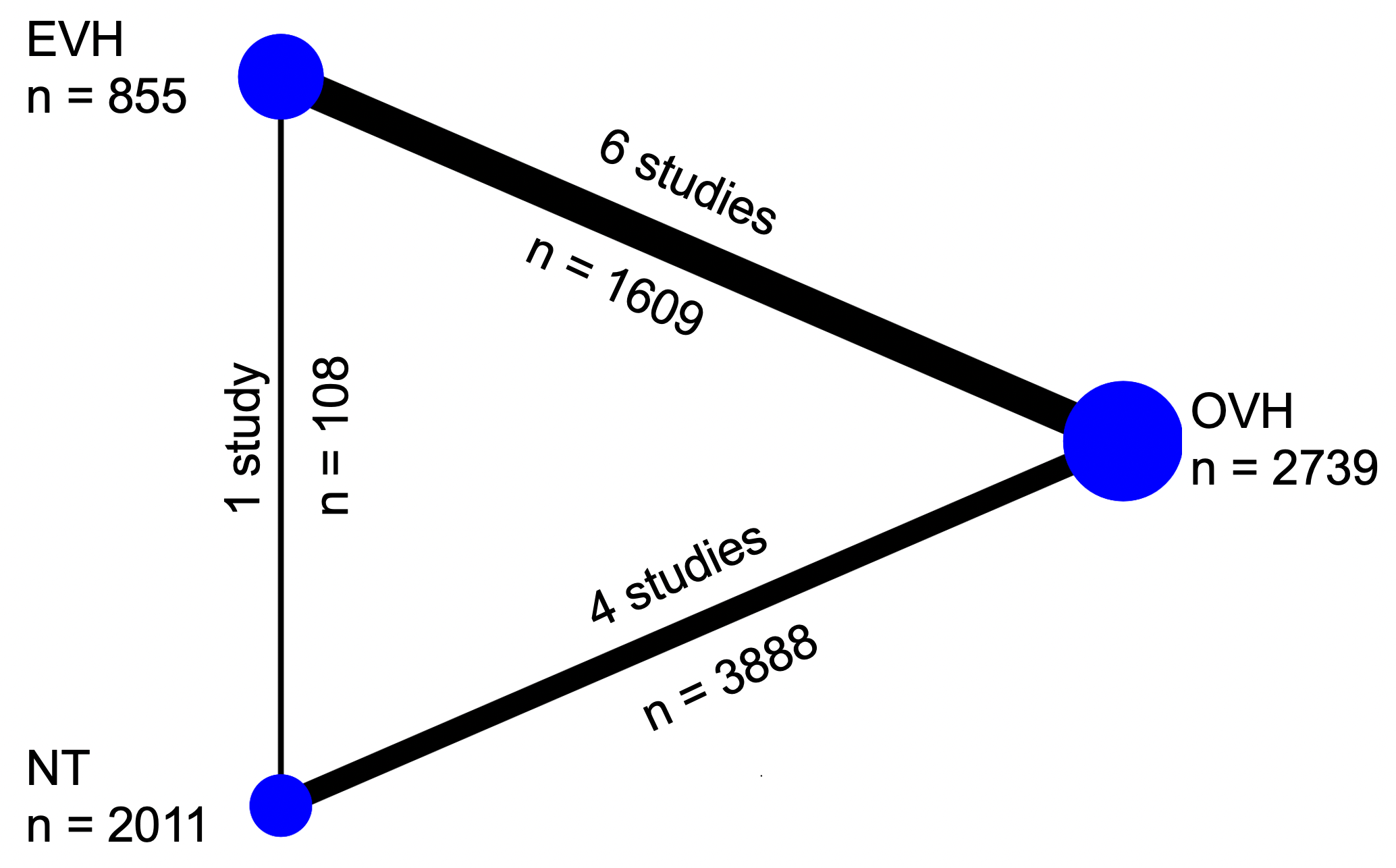


Figure S3-5. Network diagram of myocardial infarction.


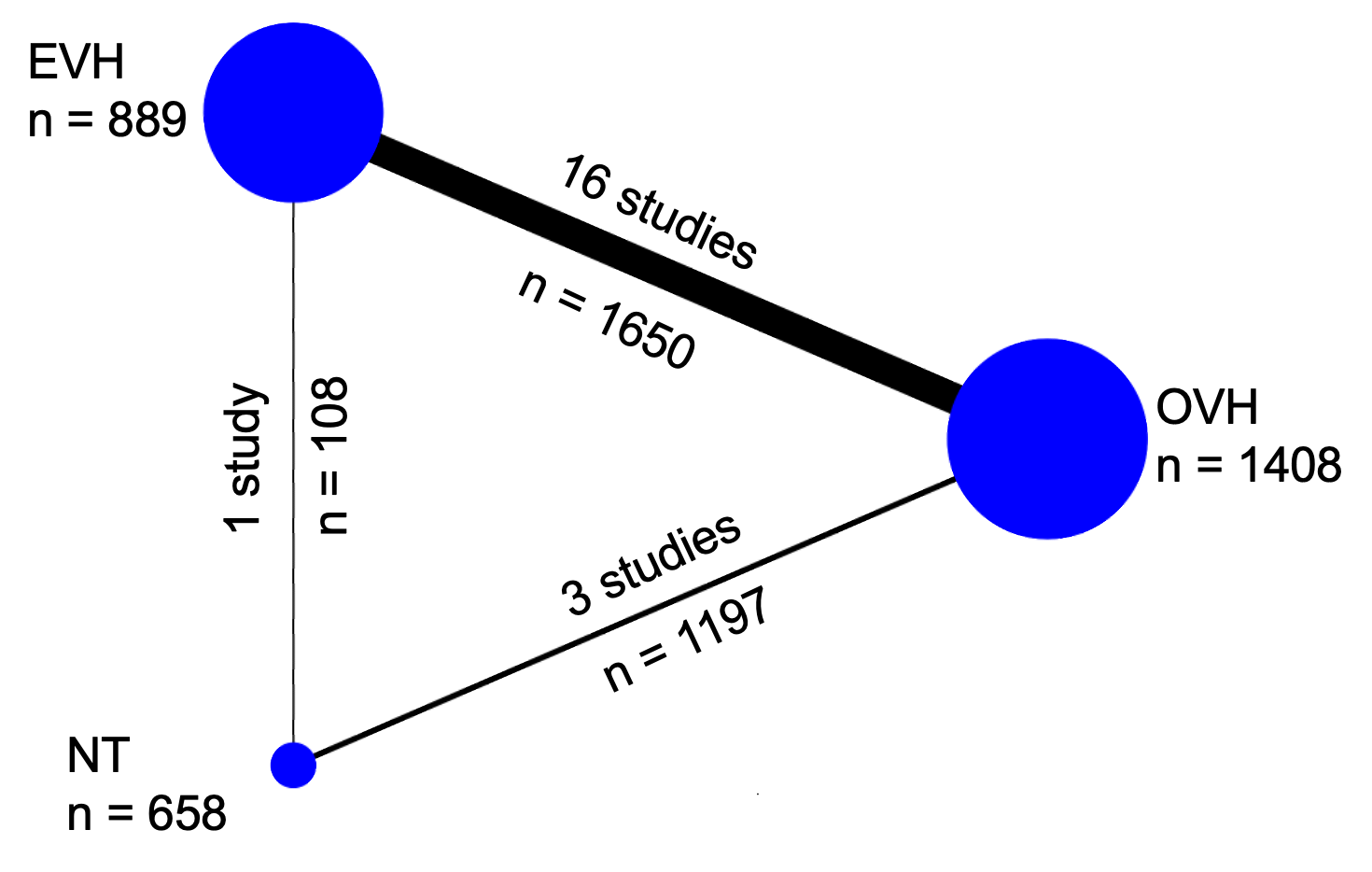


Figure S3-6. Network diagram of leg wound infection.


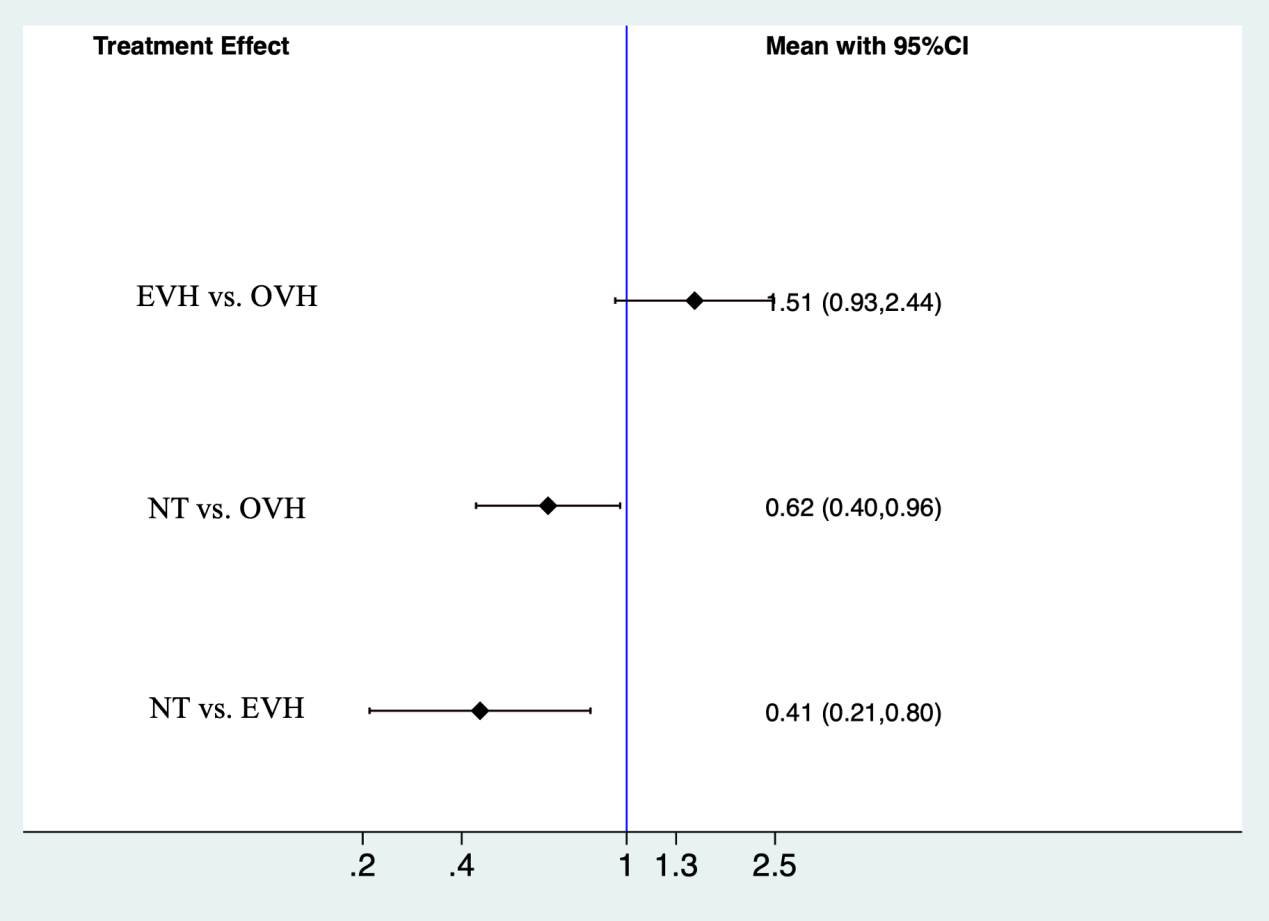


Figure S4-1. Network meta-analysis of graft failure.


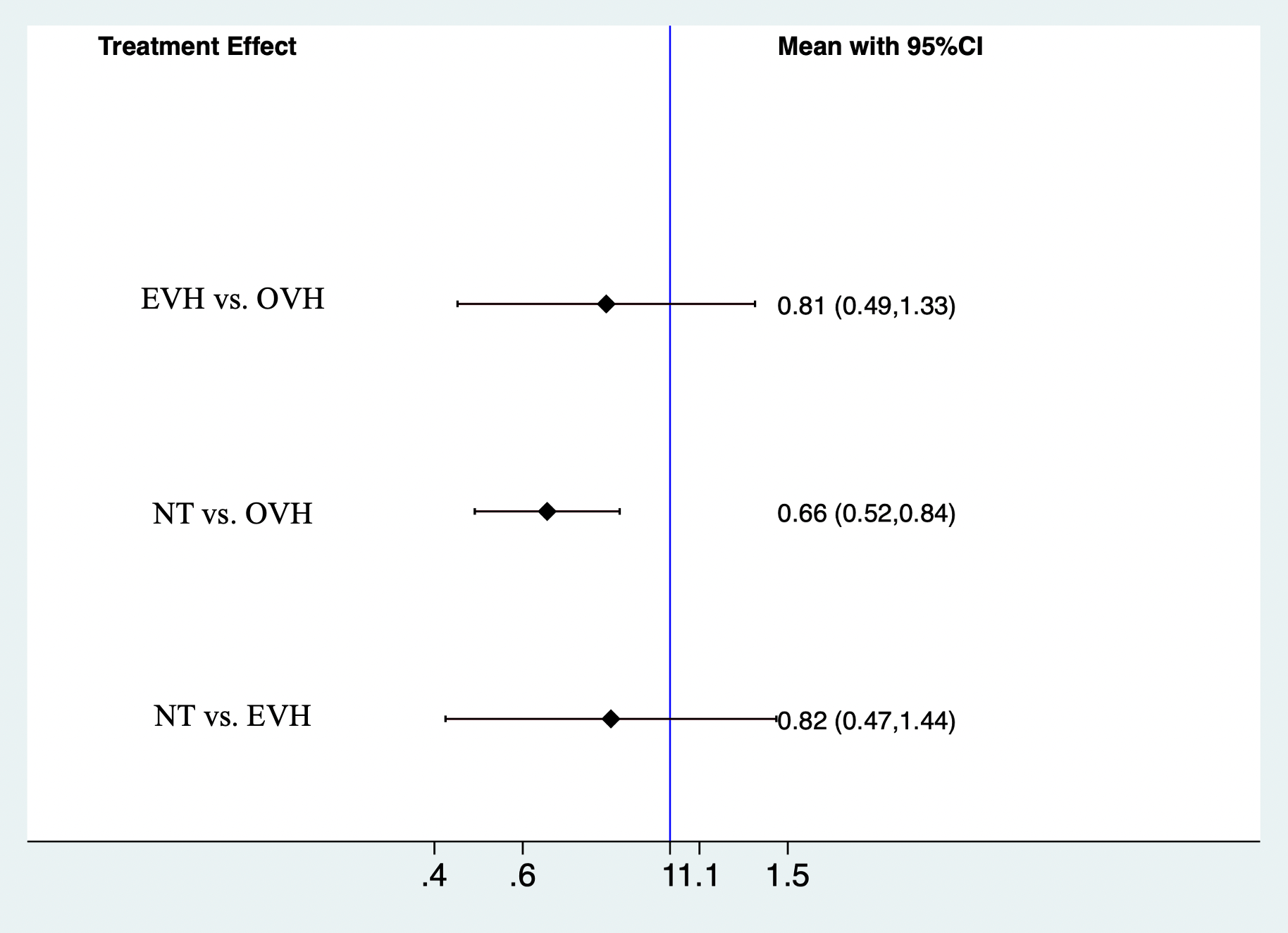


Figure S4-2. Network meta-analysis of graft occulsion.


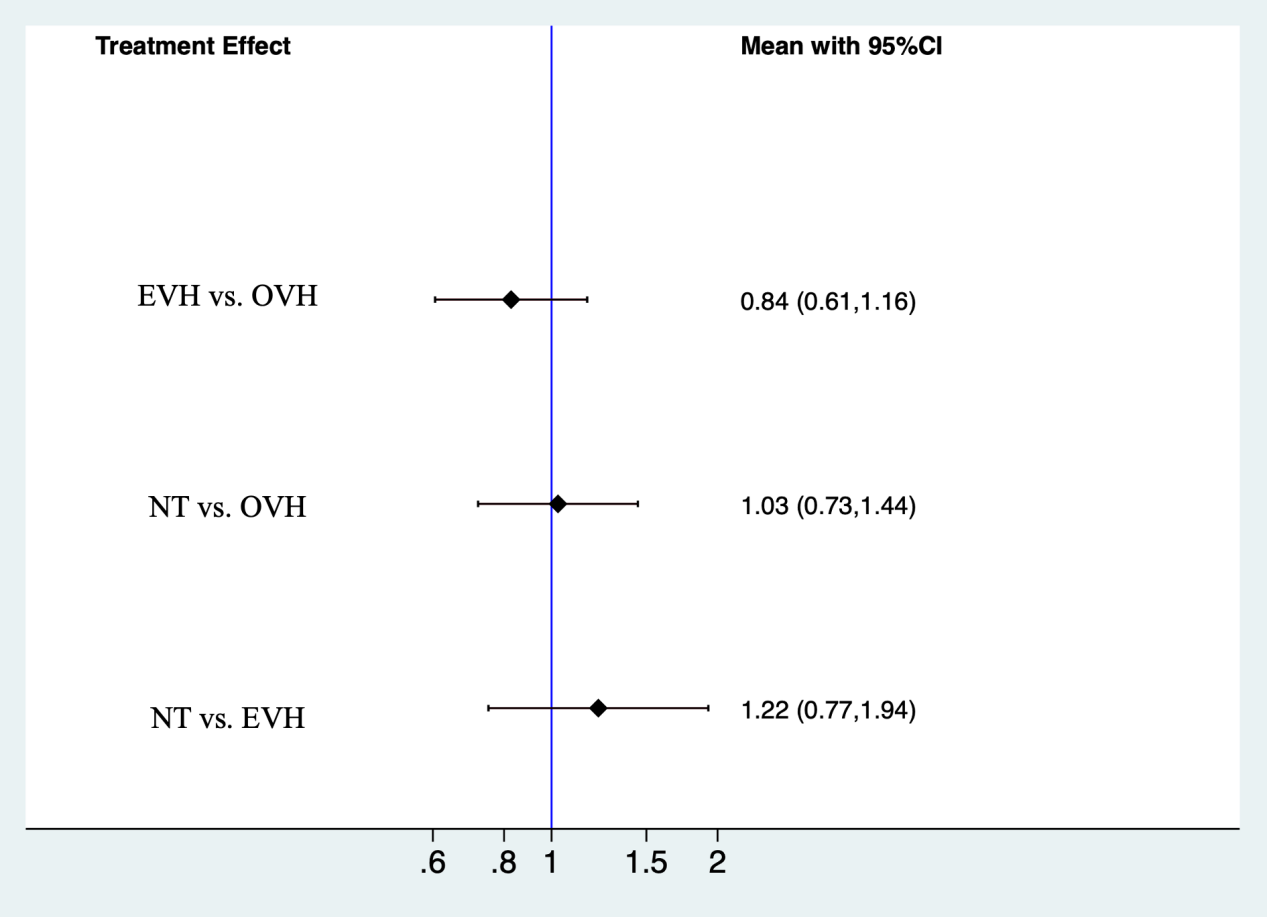


Figure S4-3. Network meta-analysis of mortality.


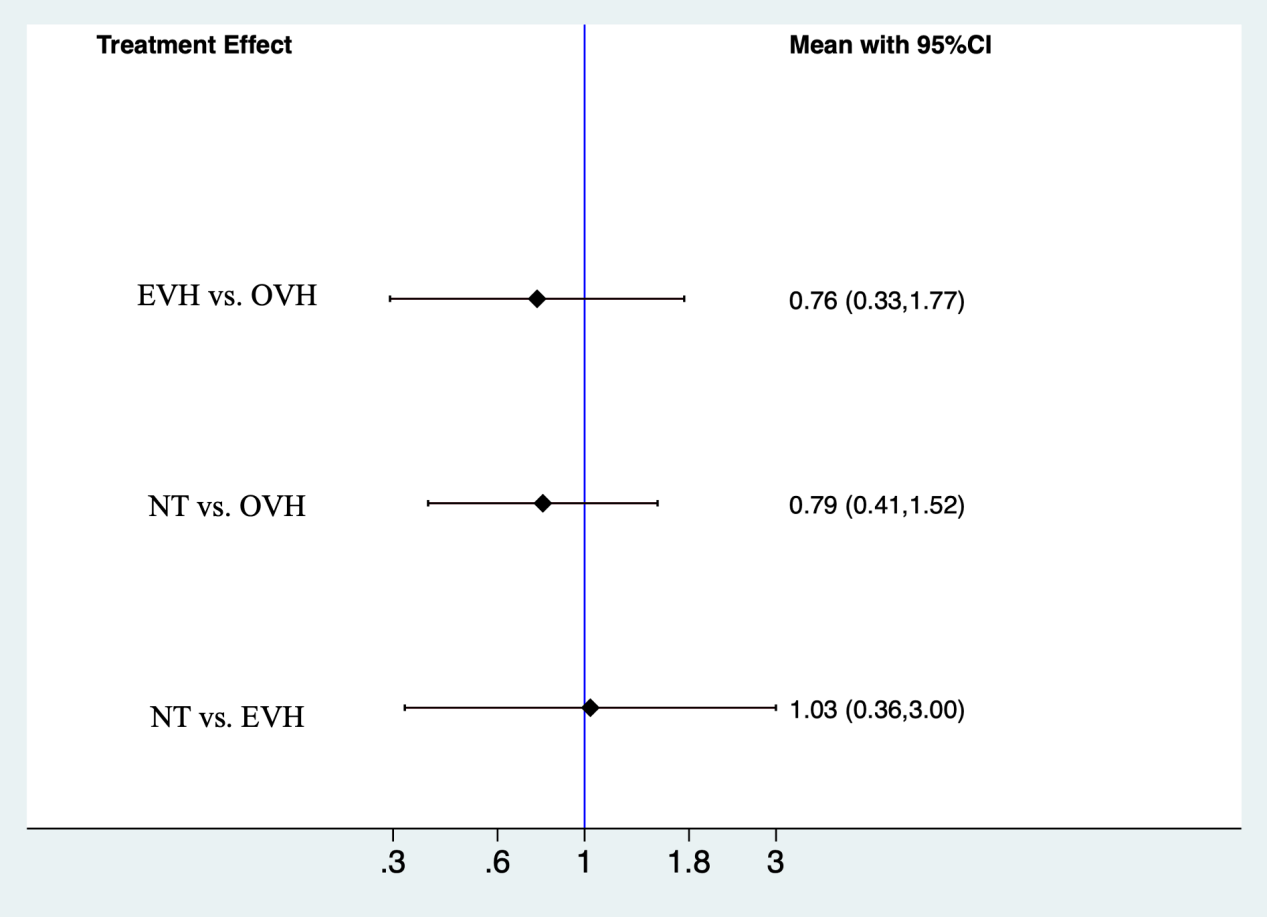


Figure S4-4. Network meta-analysis of revascularization.


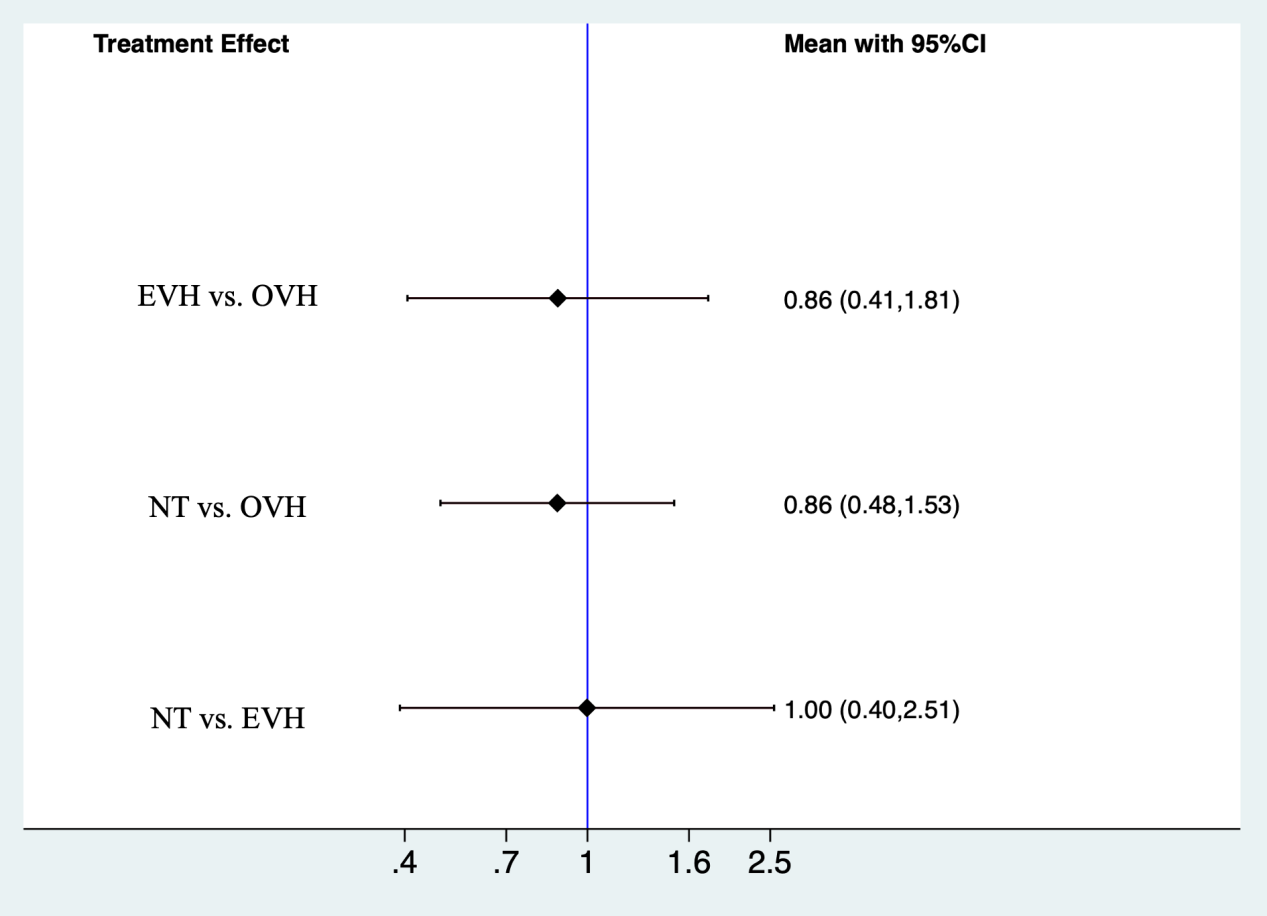


Figure S4-5. Network meta-analysis of myocardial infarction.


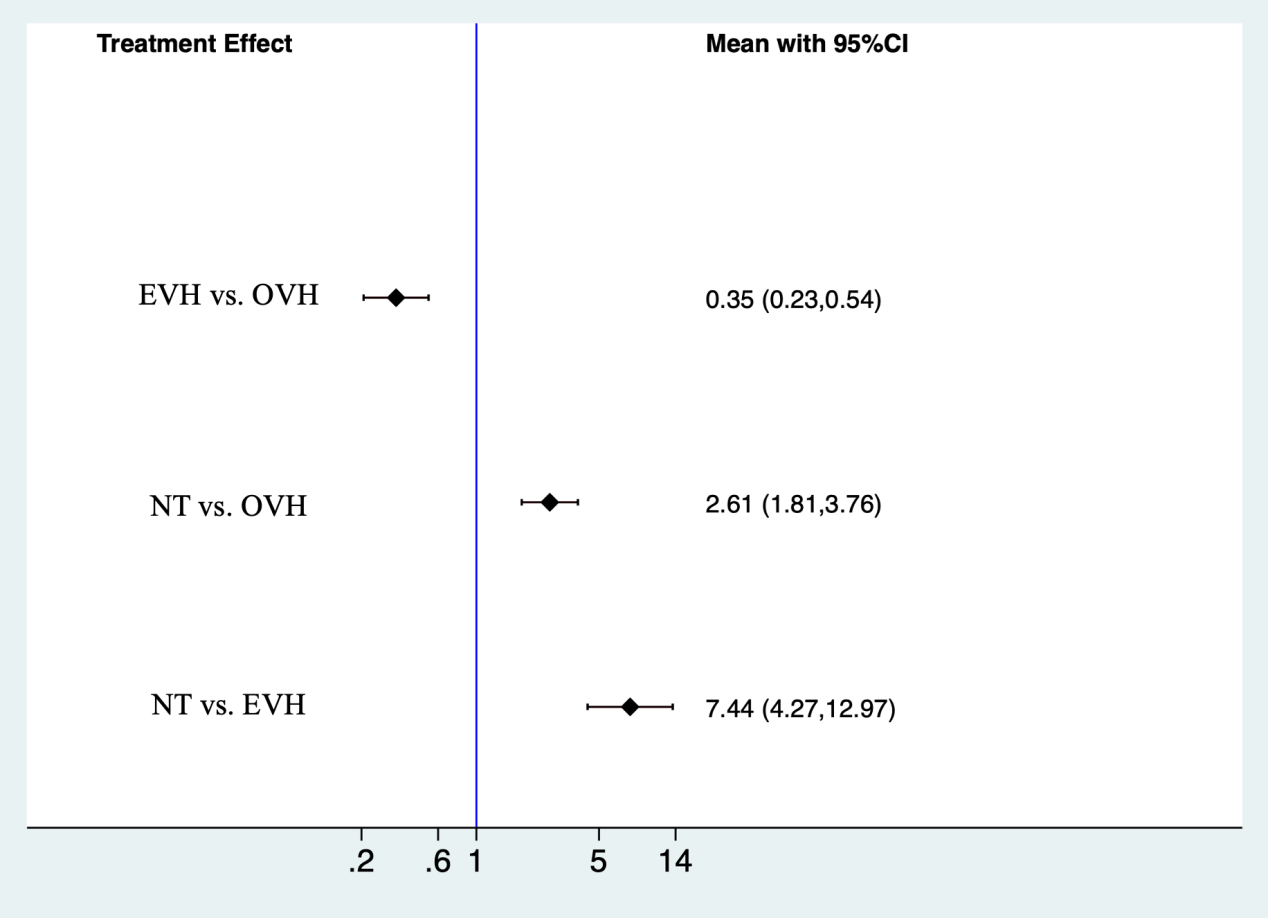


Figure S4-6. Network meta-analysis of leg wound infection.


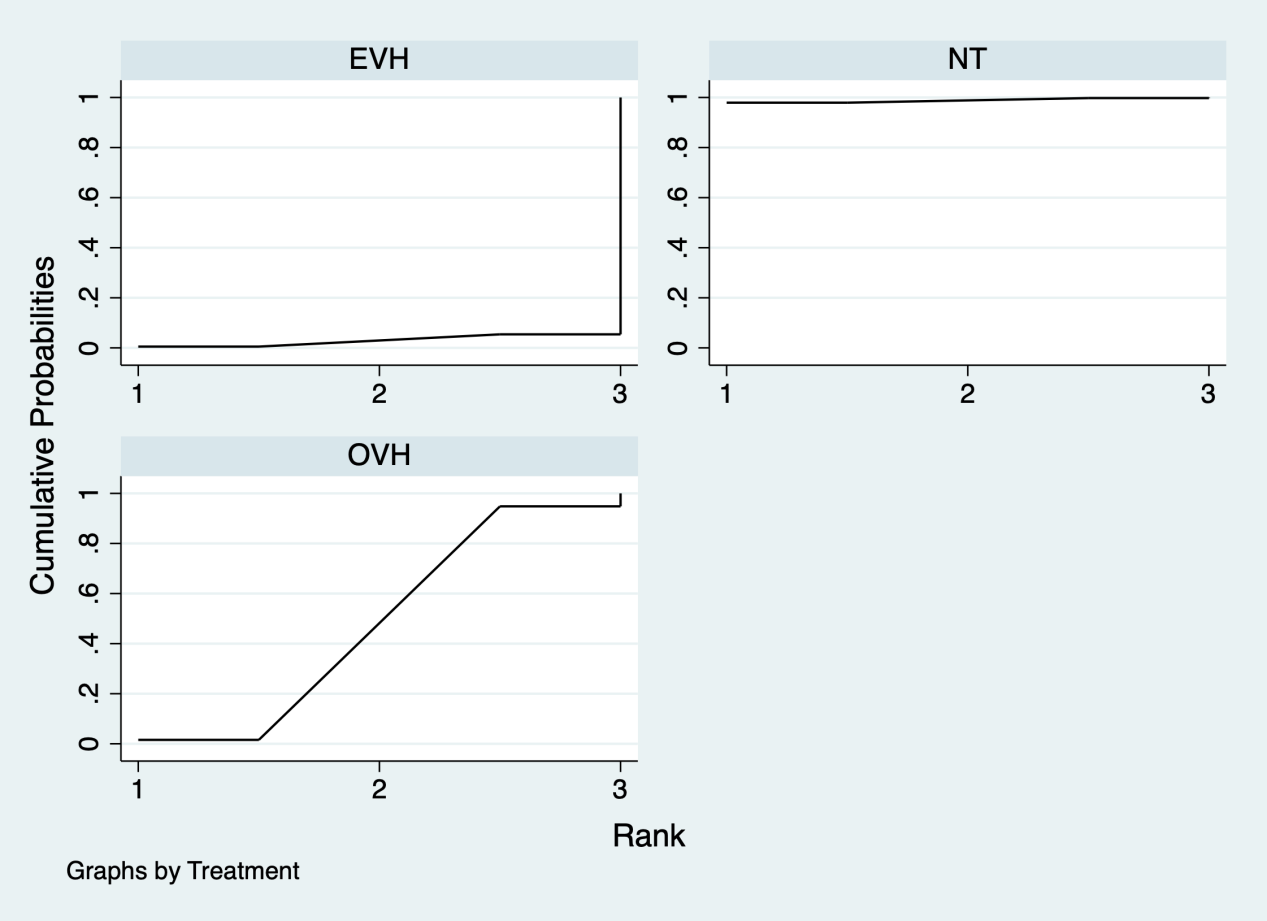


Figure S5-1.Plots of cumulative ranking probability on graft failure. (SUCRA)


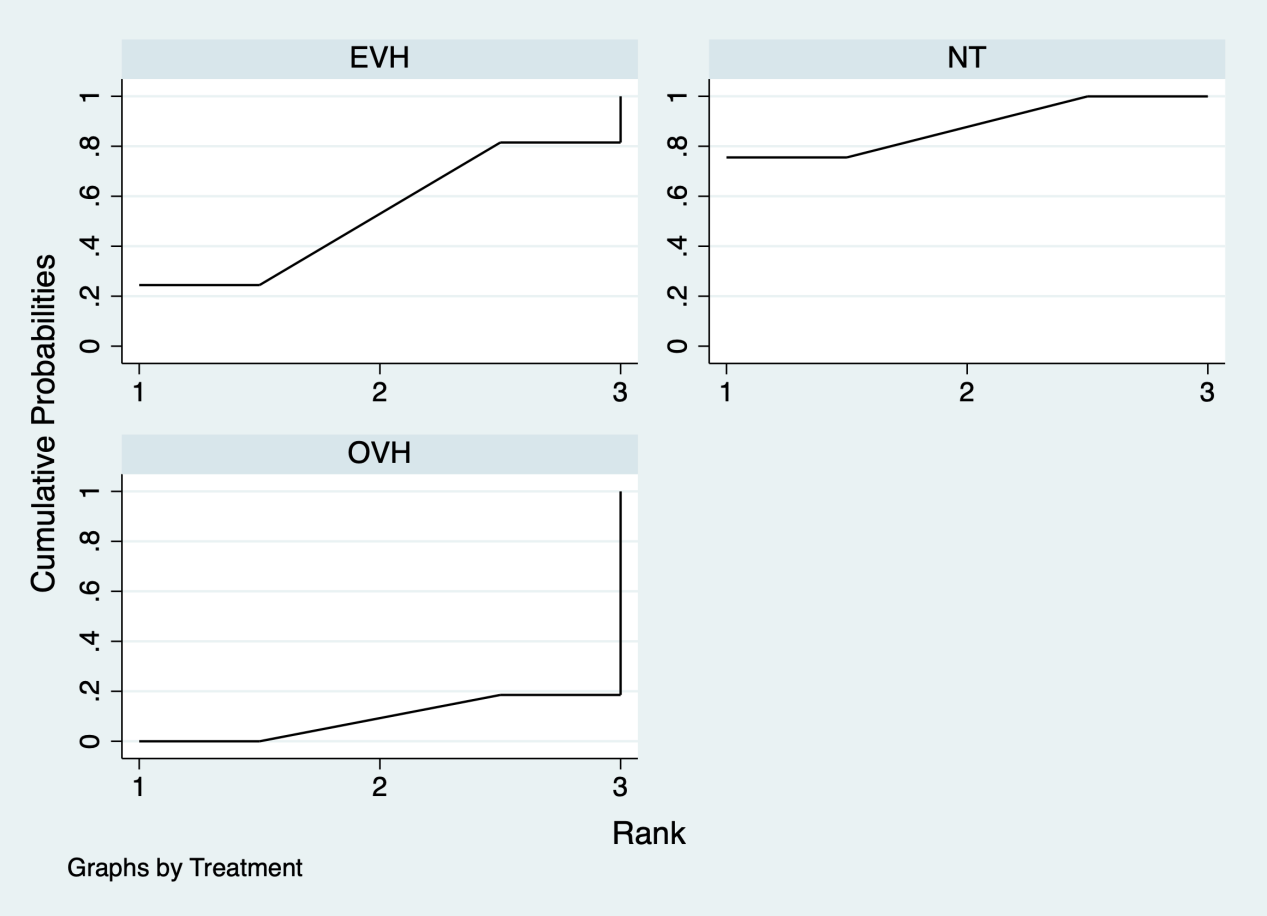


Figure S5-2.Plots of cumulative ranking probability on graft occlusion. (SUCRA)


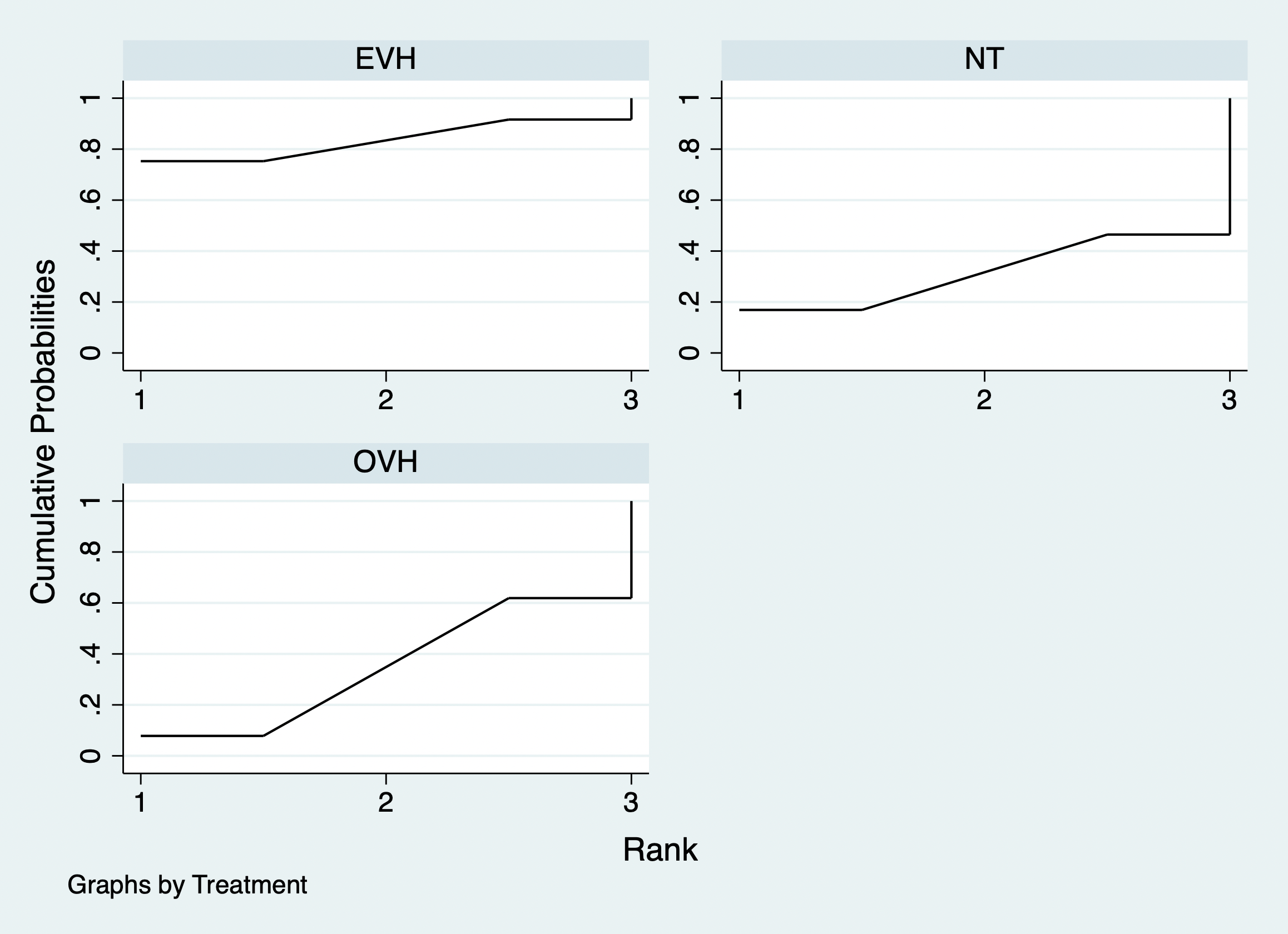


Figure S5-3.Plots of cumulative ranking probability on mortality. (SUCRA)


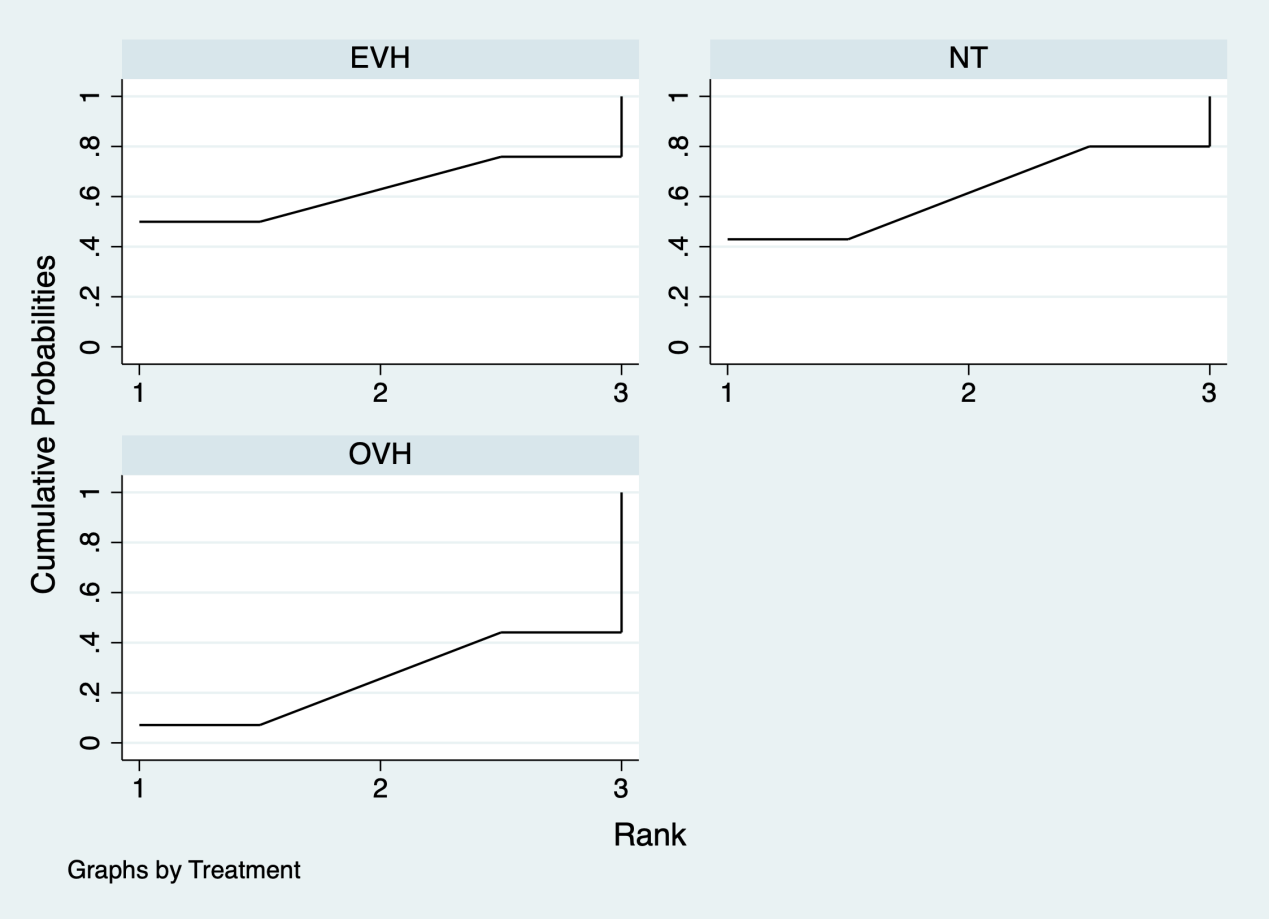


Figure S5-4.Plots of cumulative ranking probability on revascularization. (SUCRA)


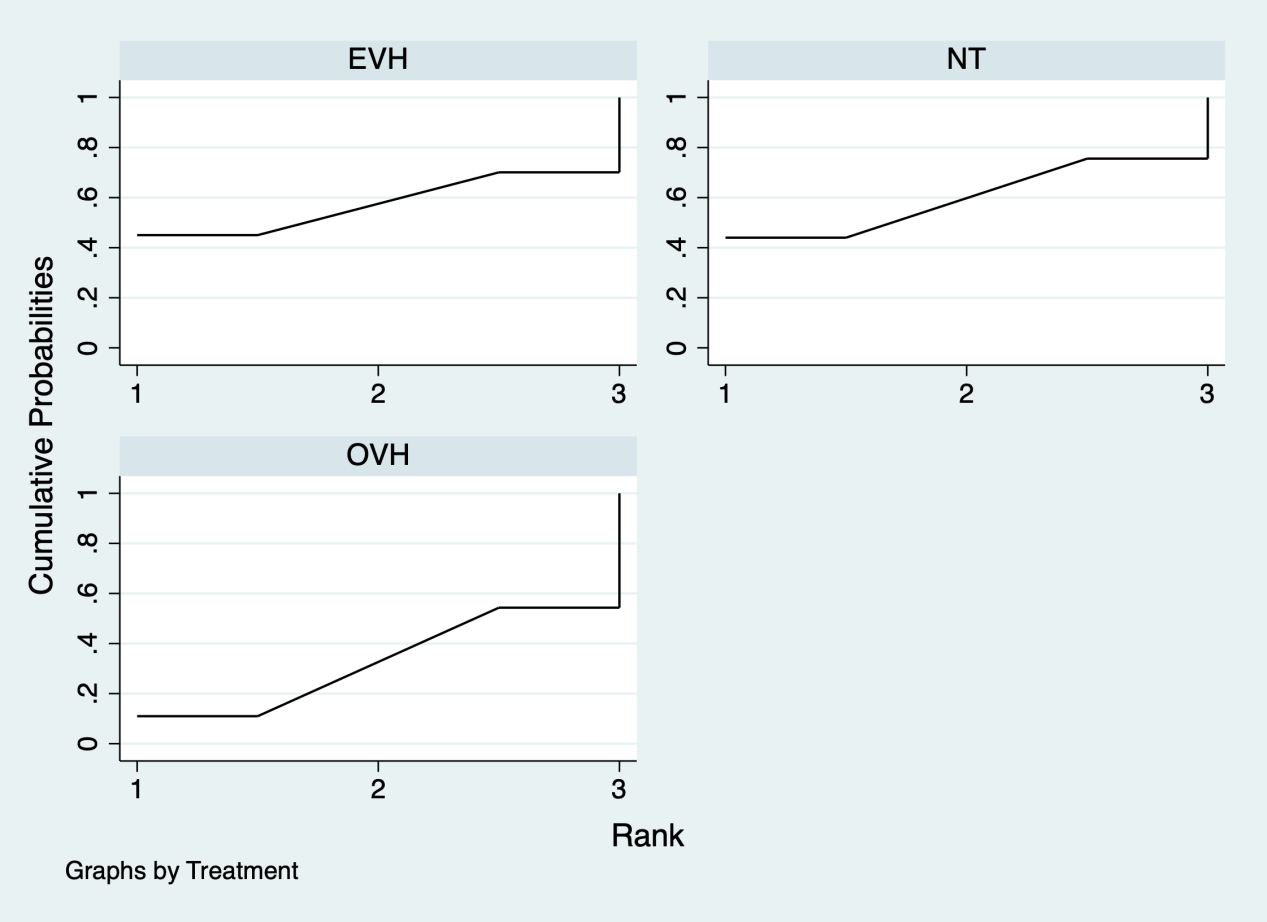


Figure S5-5.Plots of cumulative ranking probability on myocardiac infarction. (SUCRA)


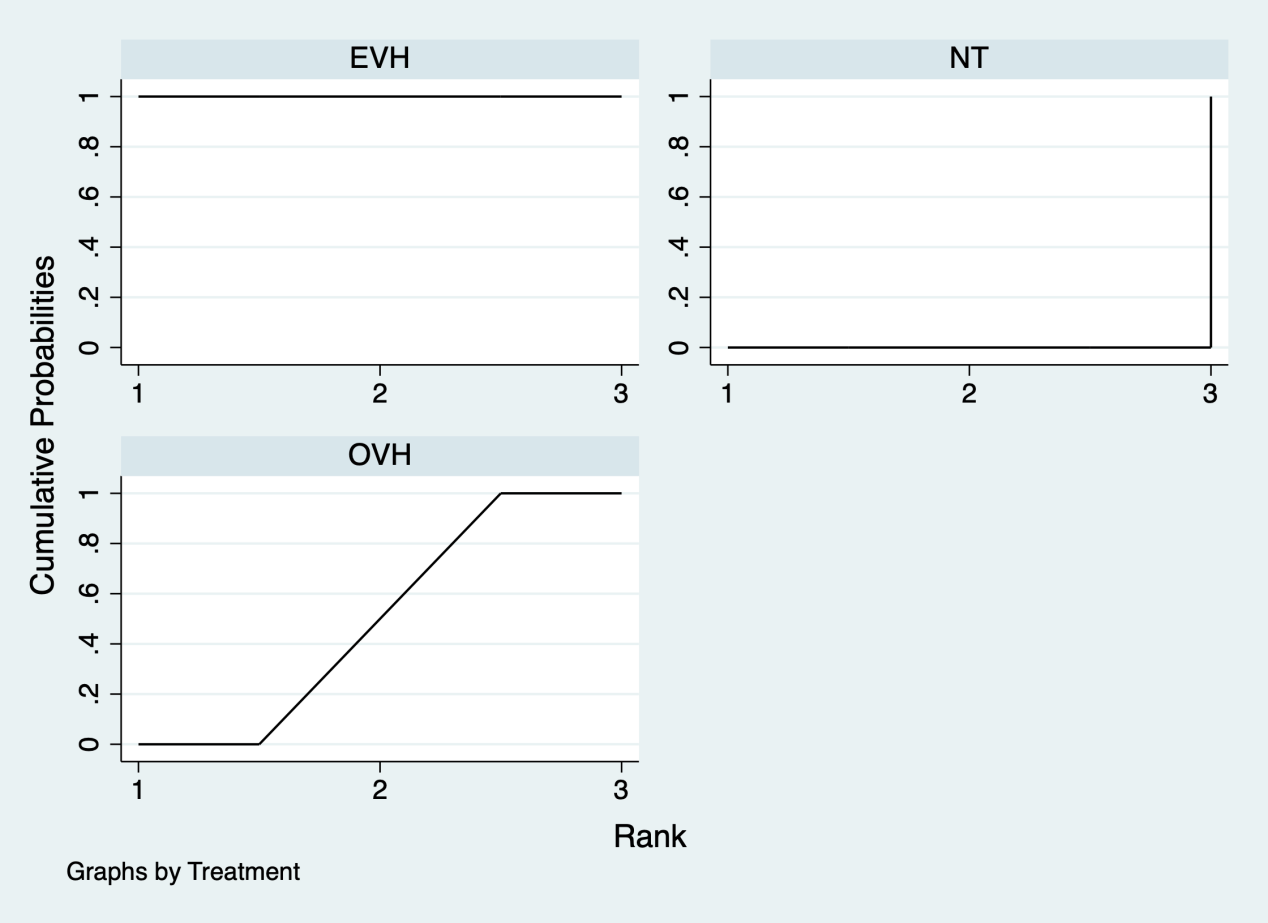


Figure S5-6.Plots of cumulative ranking probability on leg wound infection. (SUCRA)


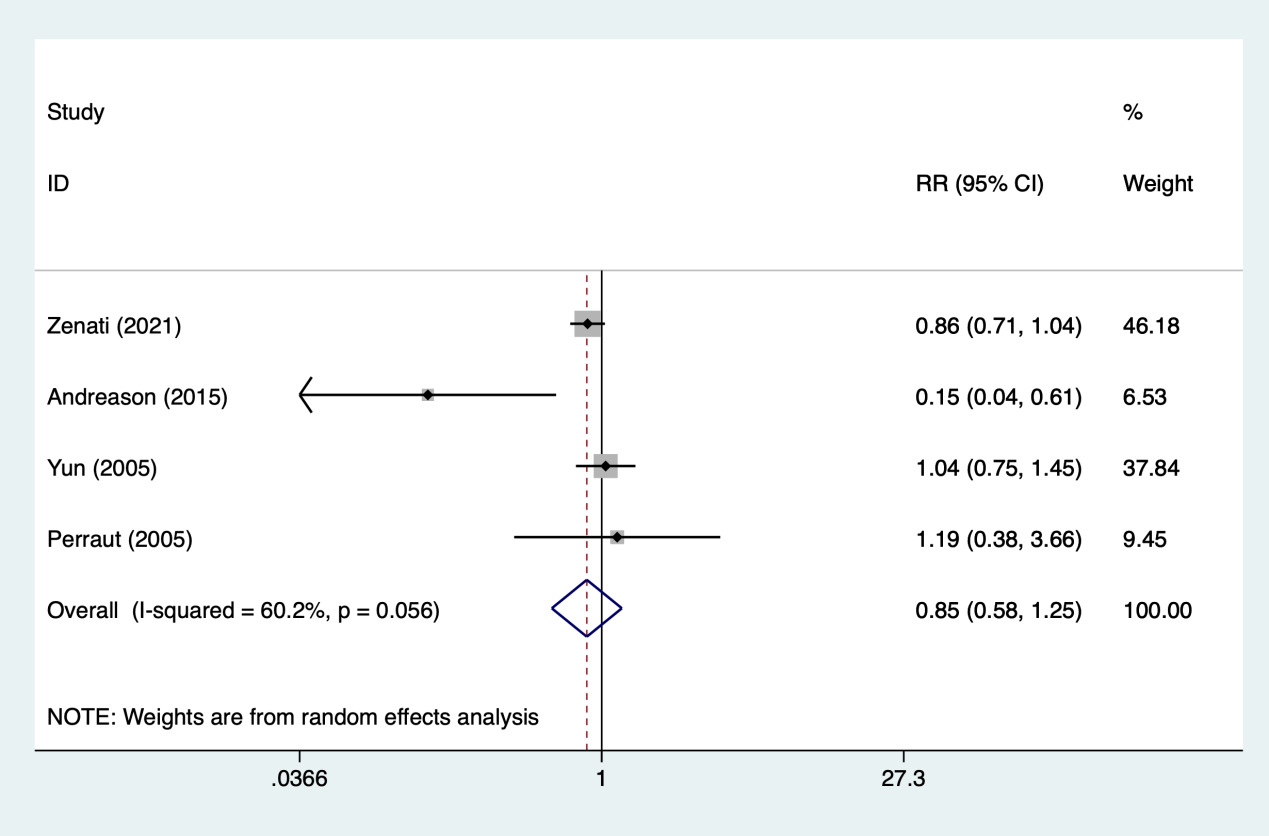


Figure S6-1.Graft failure among OVH vs EVH.


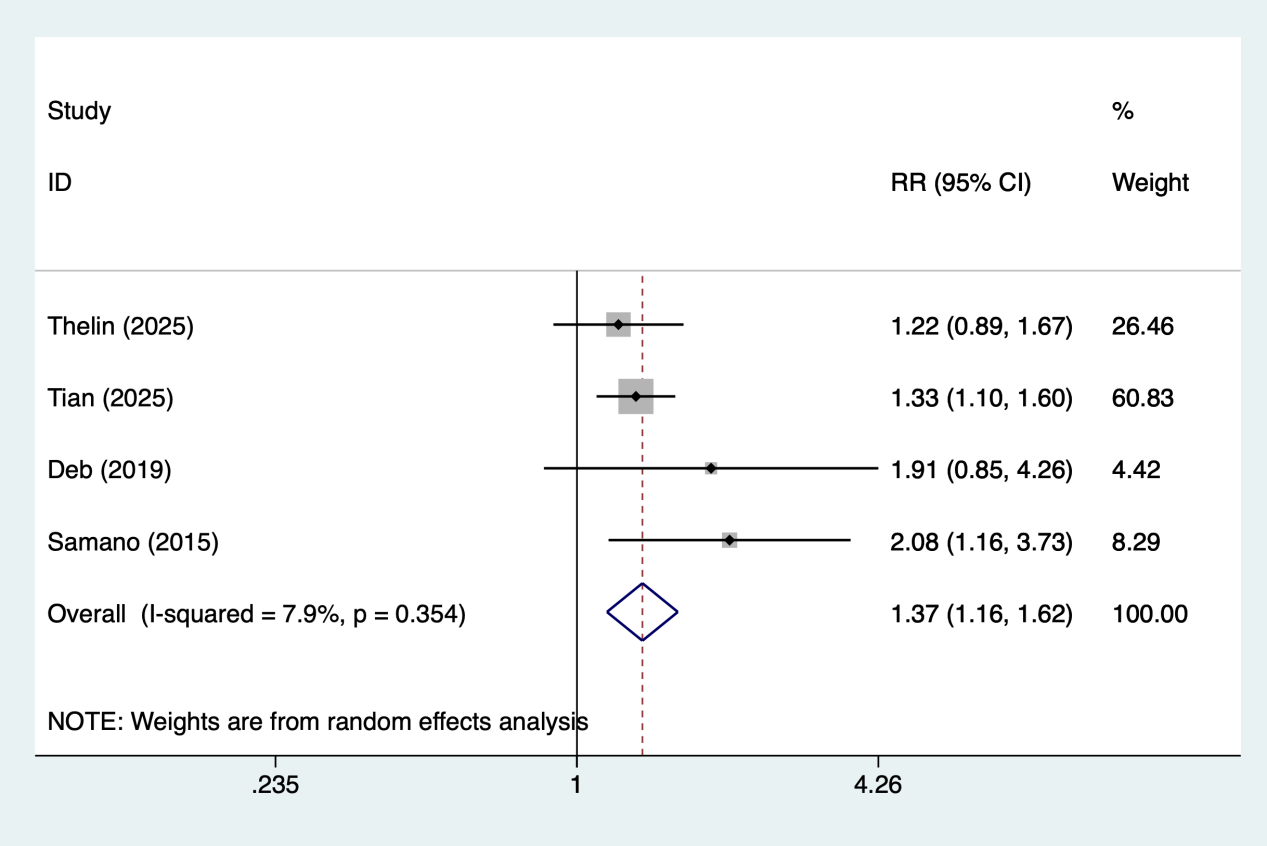


Figure S6-2.Graft failure among OVH vs NT.


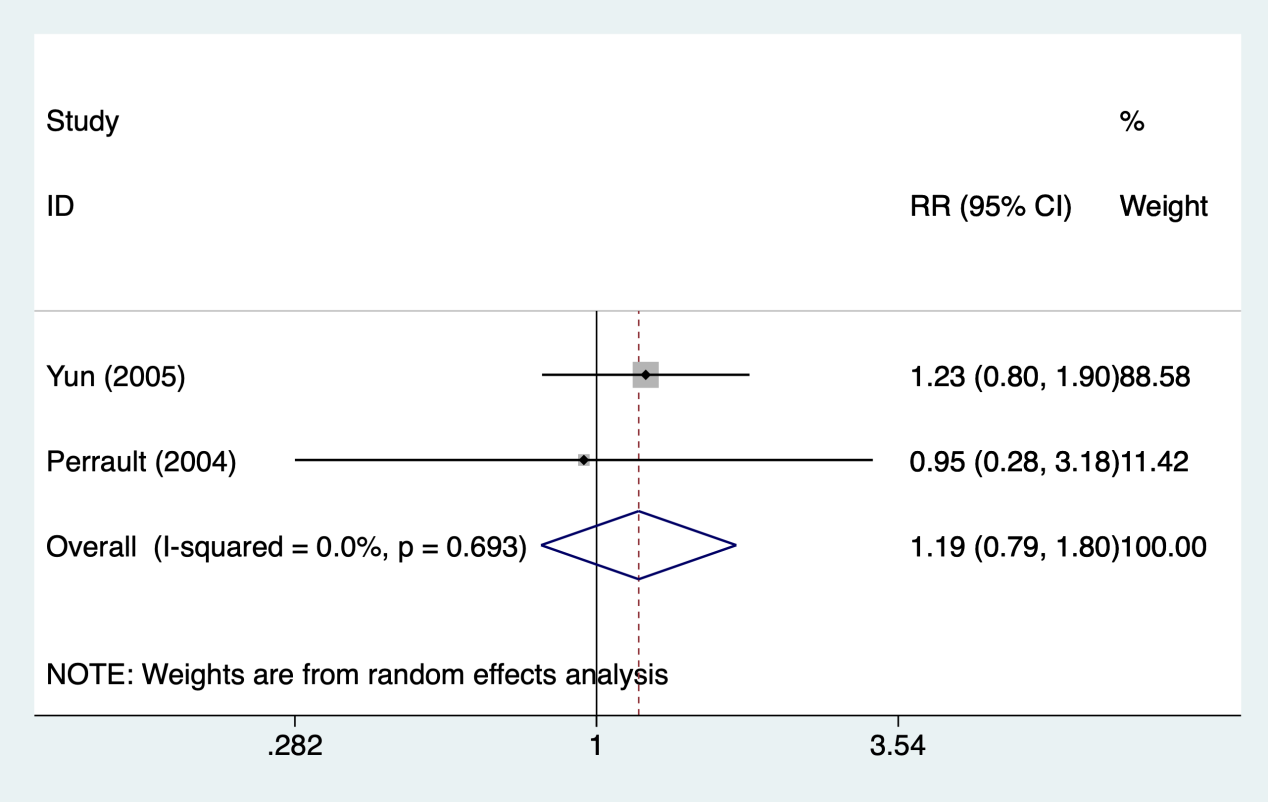


Figure S6-3.Graft occlusion among OVH vs EVH.


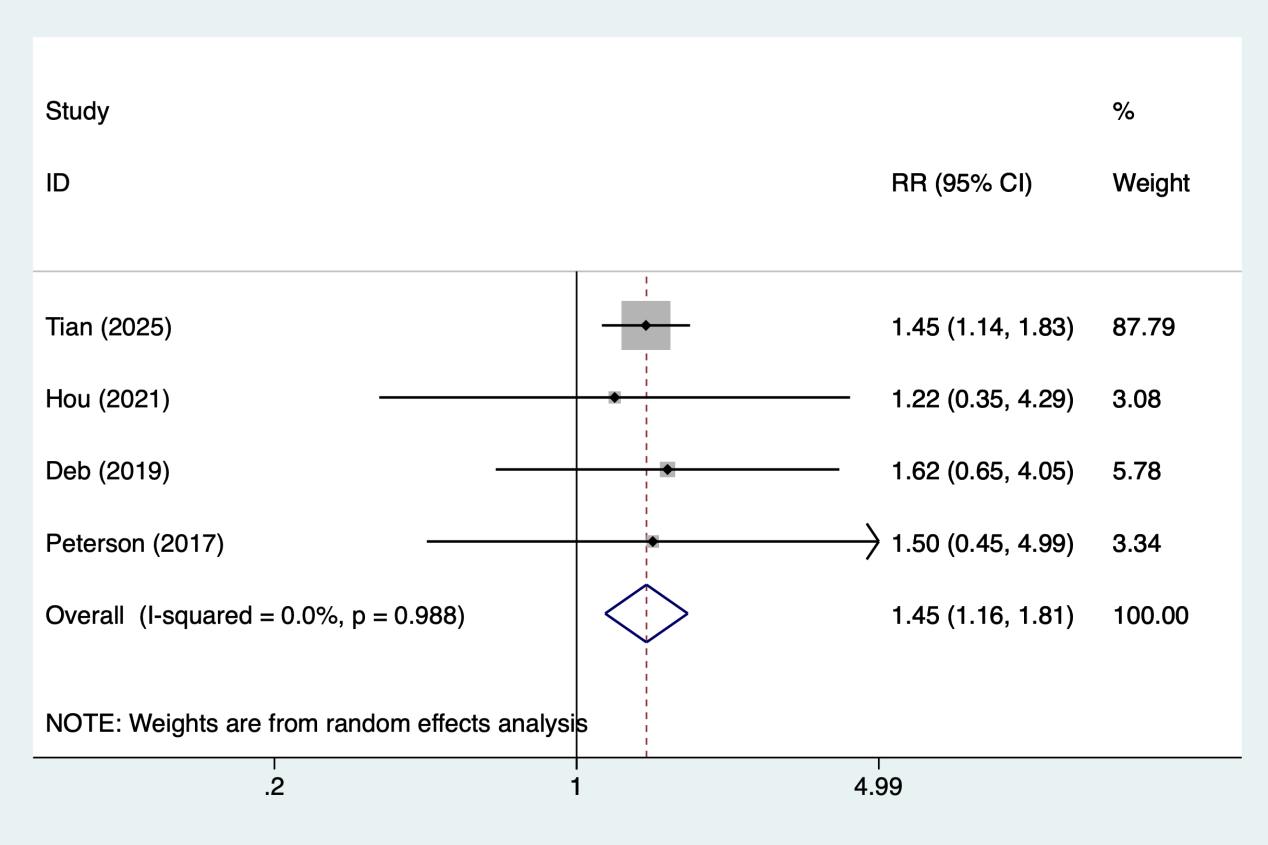


Figure S6-4.Graft occlusion among OVH vs NT.


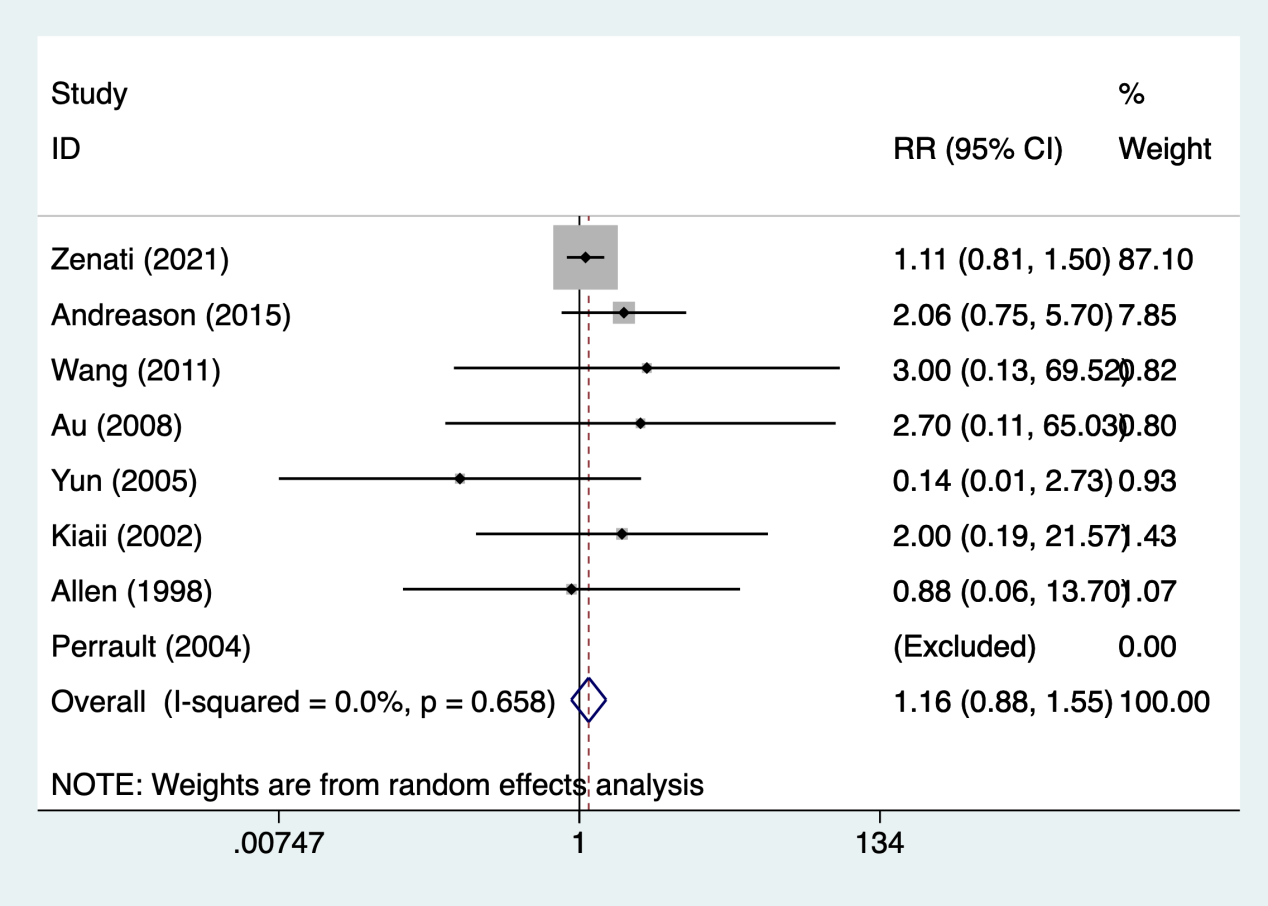


Figure S6-5.Mortality among OVH vs EVH.


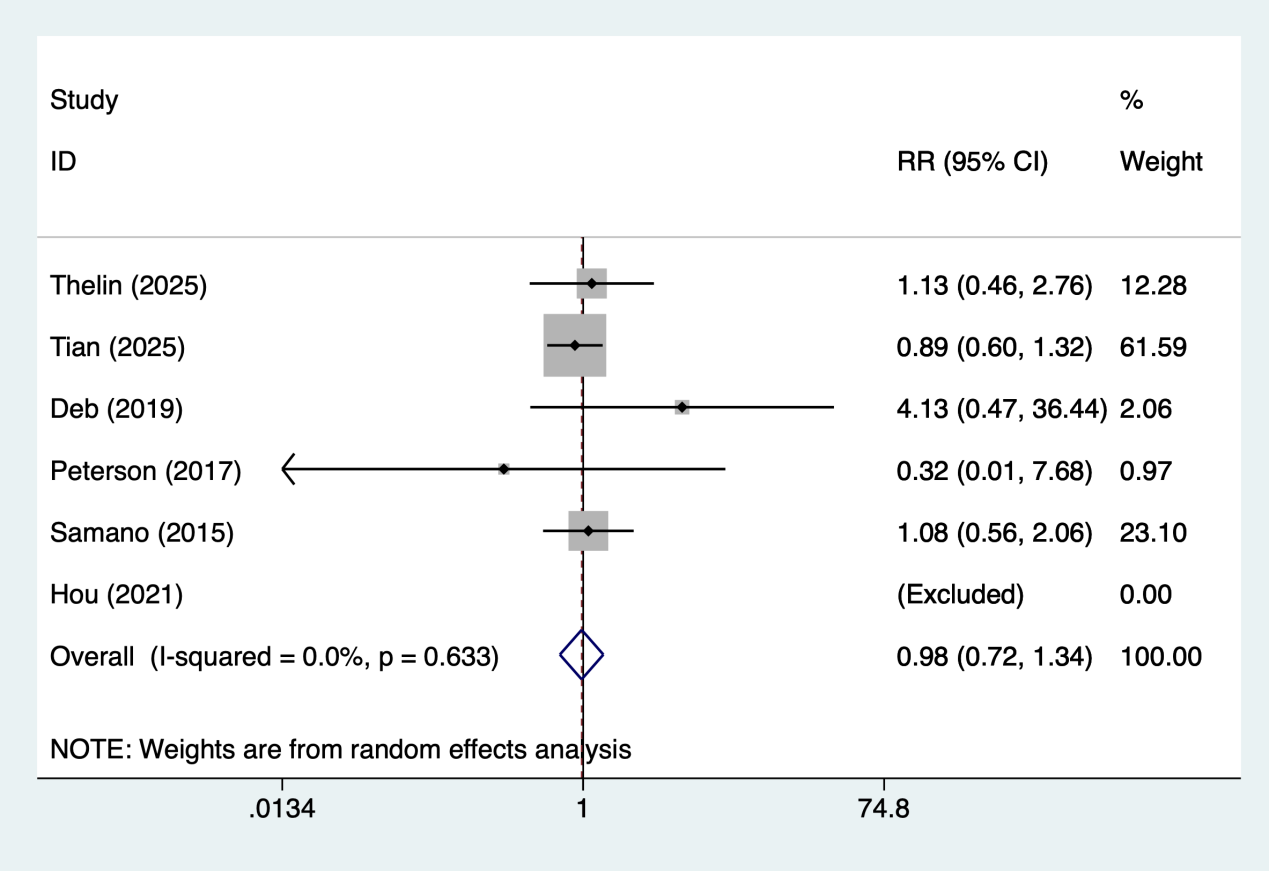


Figure S6-6.Mortality among OVH vs NT.


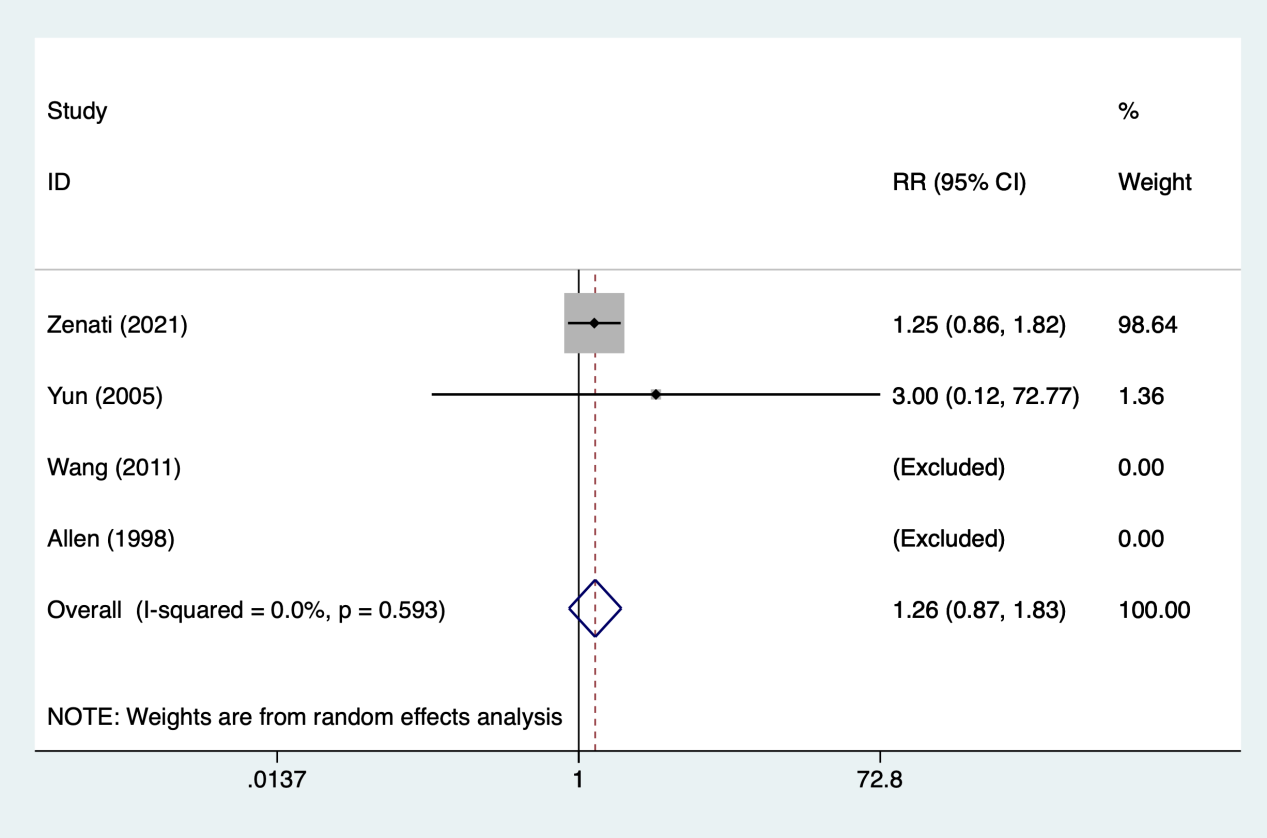


Figure S6-7.Revascularization among OVH vs EVH.


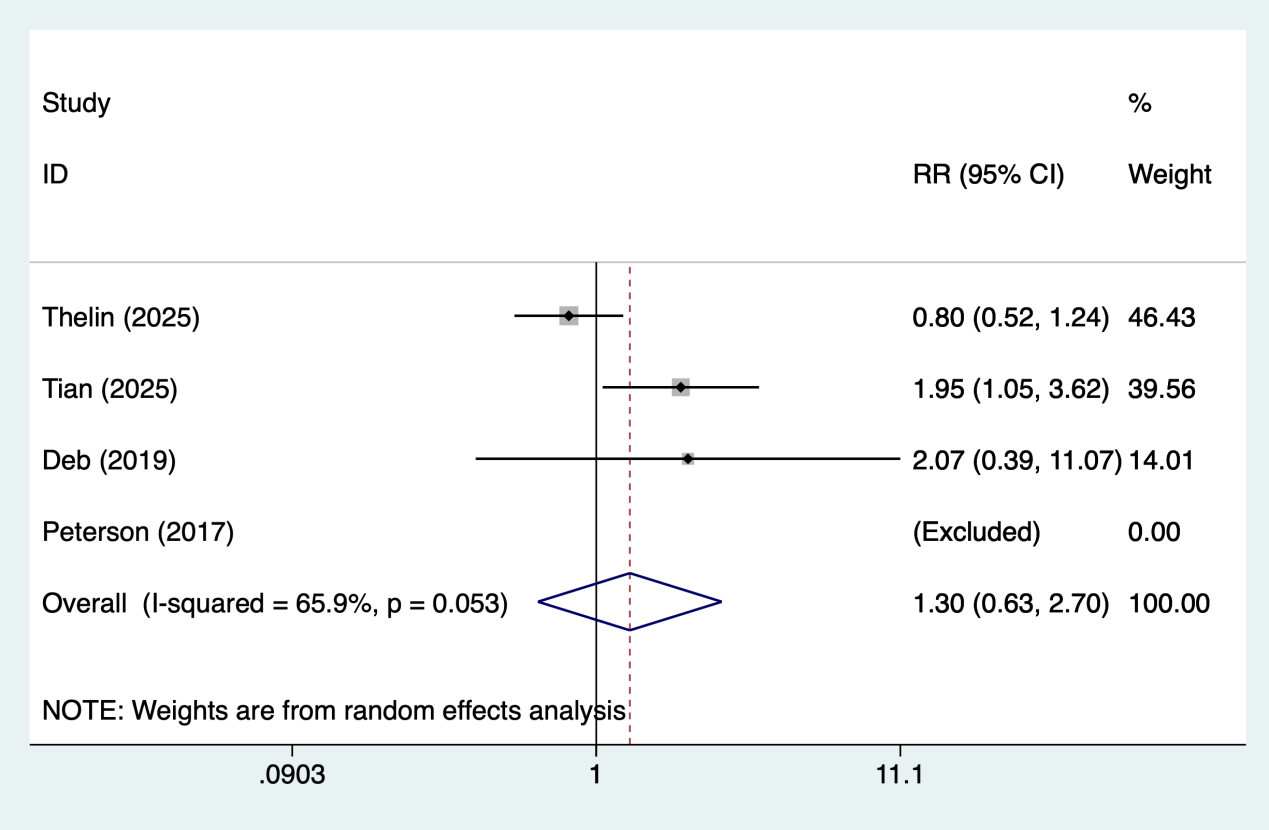


Figure S6-8.Revascularization among OVH vs NT.


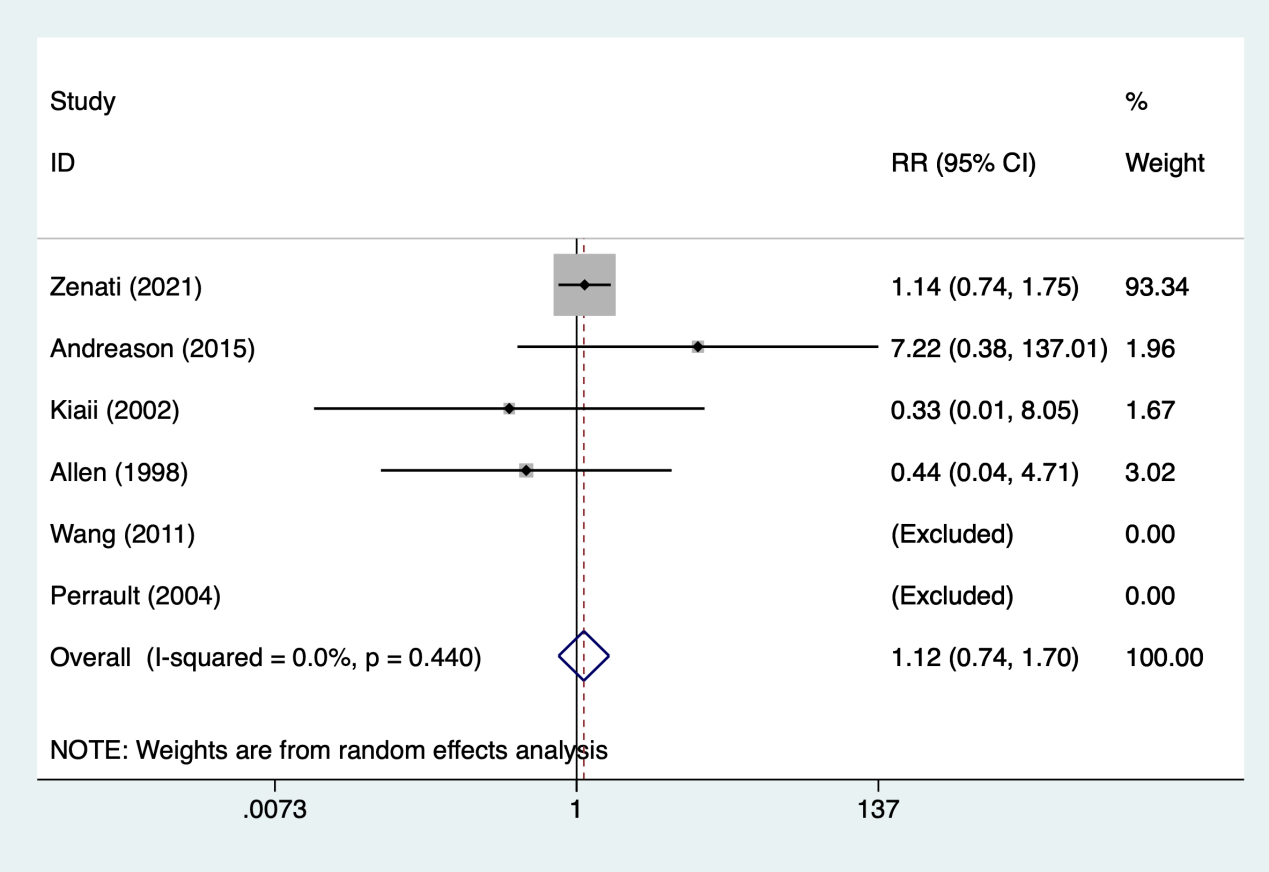


Figure S6-9.Myocardial infarction among OVH vs EVH.


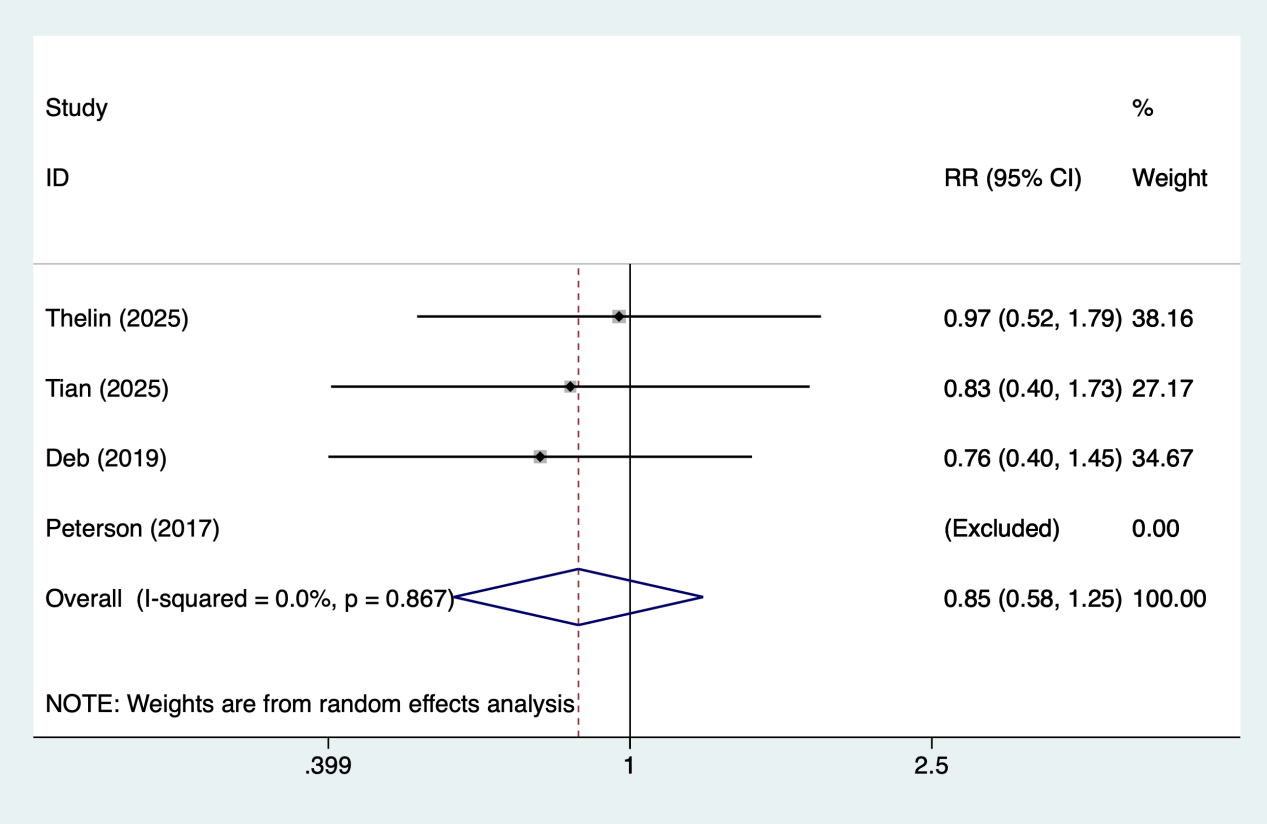


Figure S6-10.Myocardial infarction among OVH vs NT.


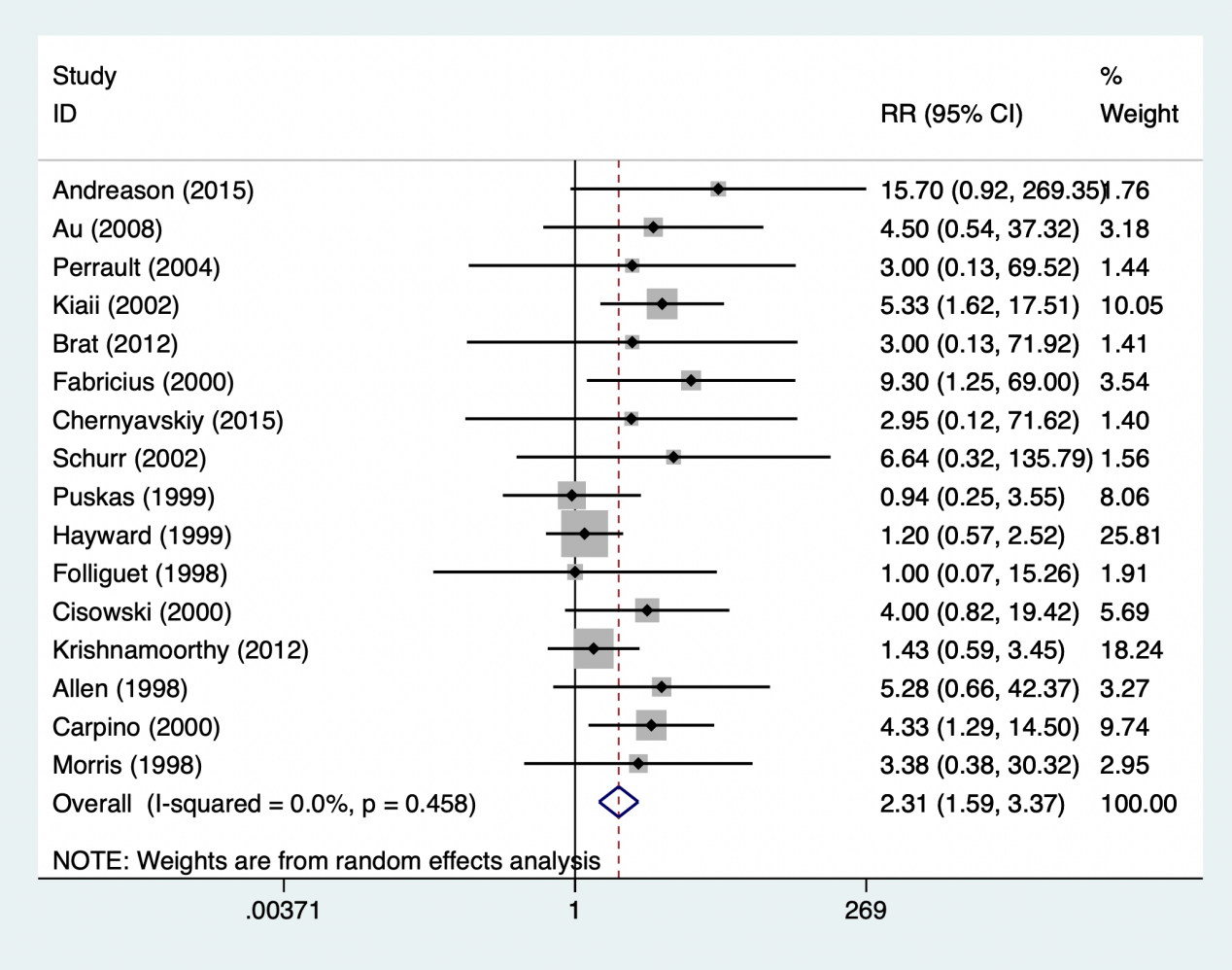


Figure S6-11.Leg wound infection among OVH vs EVH.


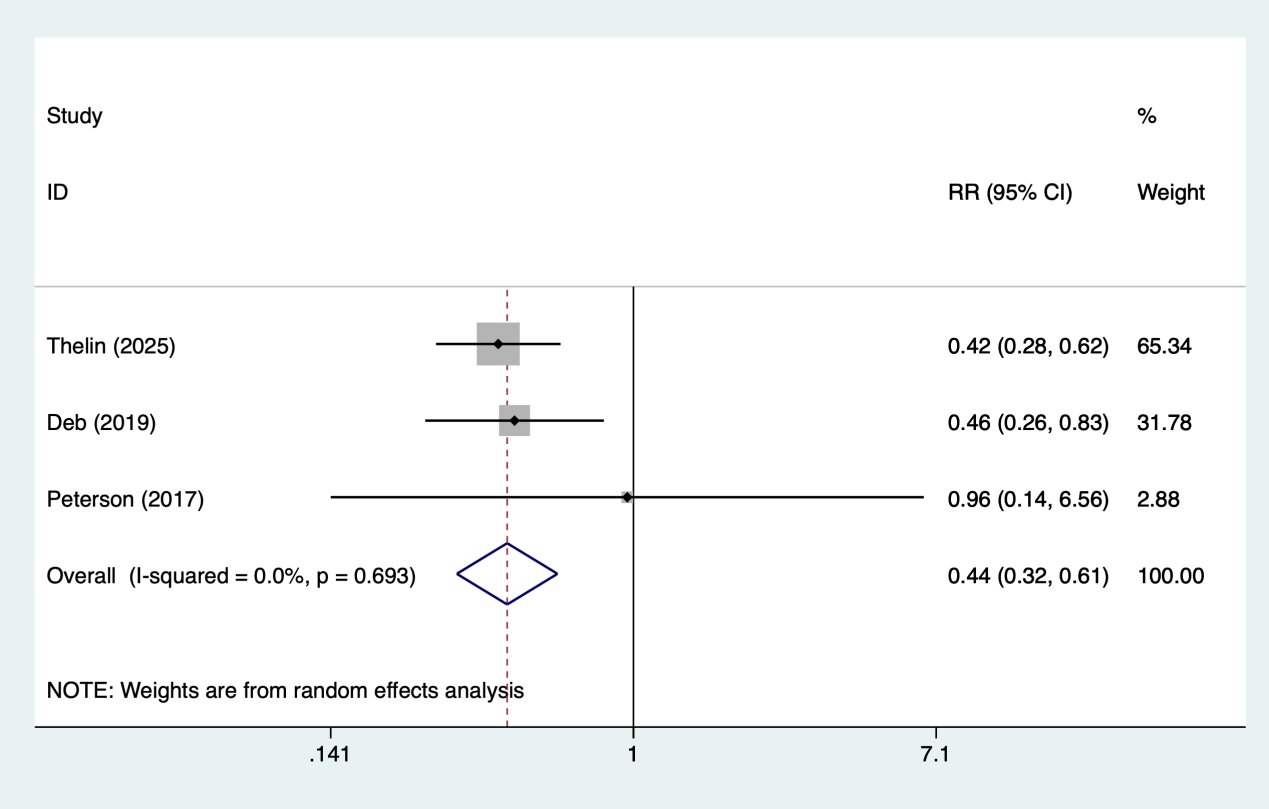


Figure S6-12.Leg wound infection among OVH vs NT.


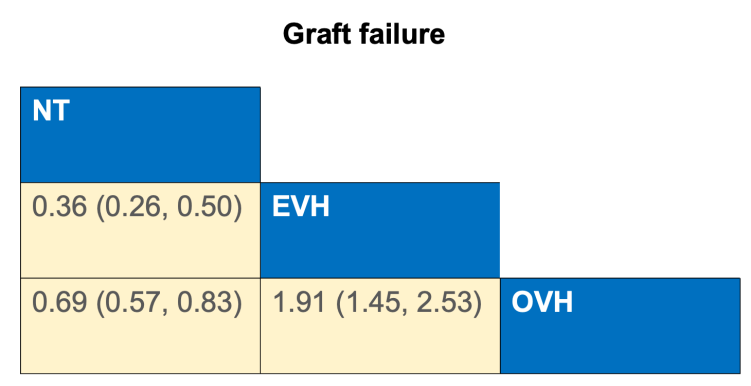


Figure S7-1. League tables for OVH, EVH, and NT (subgroup analysis). A subgroup analysis was conducted using solely the studies that had a follow-up period of at least 1 year. Outcomes shown for graft failure following OVH, EVH, and NT in subgroup analysis (RR and 95% CI). RR <1 means the treatment in top left is better. OVH = open vein harvesting; EVH = endoscopic vein harvesting; NT = no-touch vein harvesting.


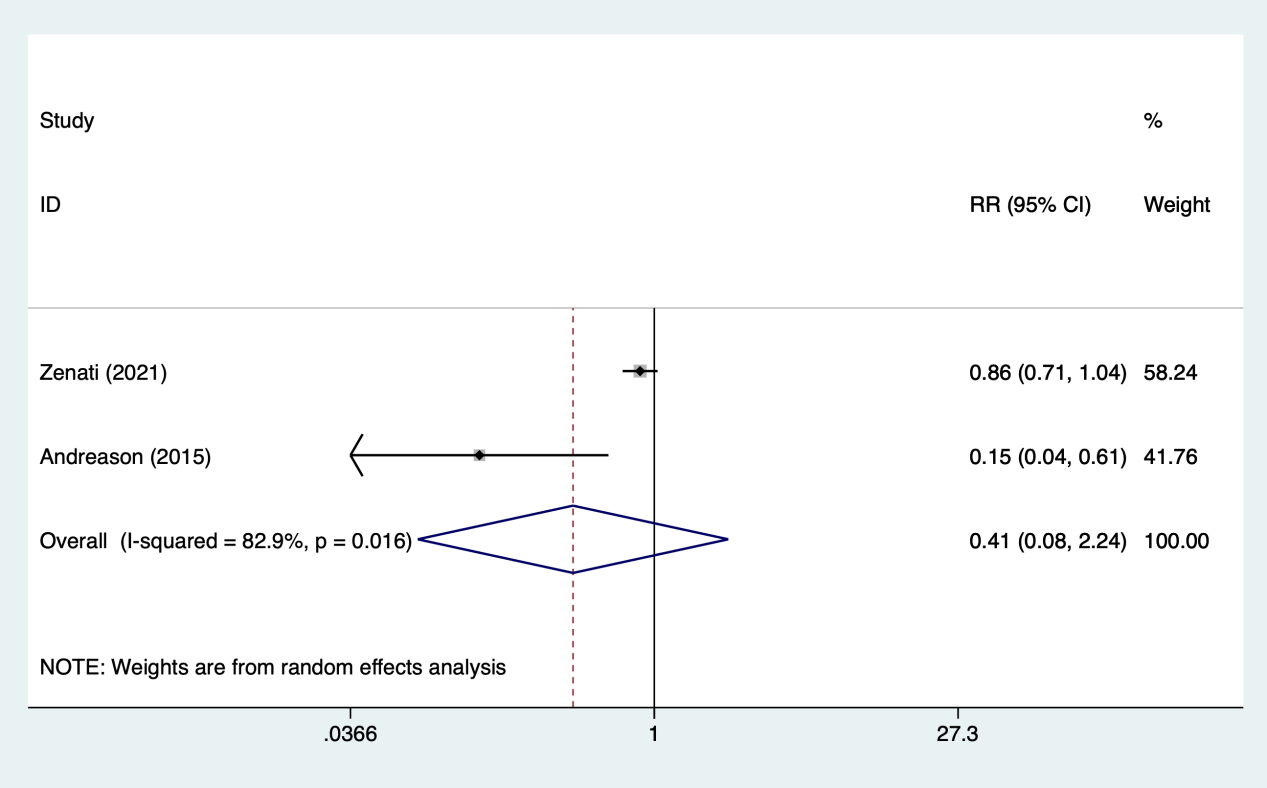


Figure S7-2.Graft failure among OVH vs EVH in subgroup analysis. A subgroup analysis was conducted using solely the studies that had a follow-up period of at least 1 year.


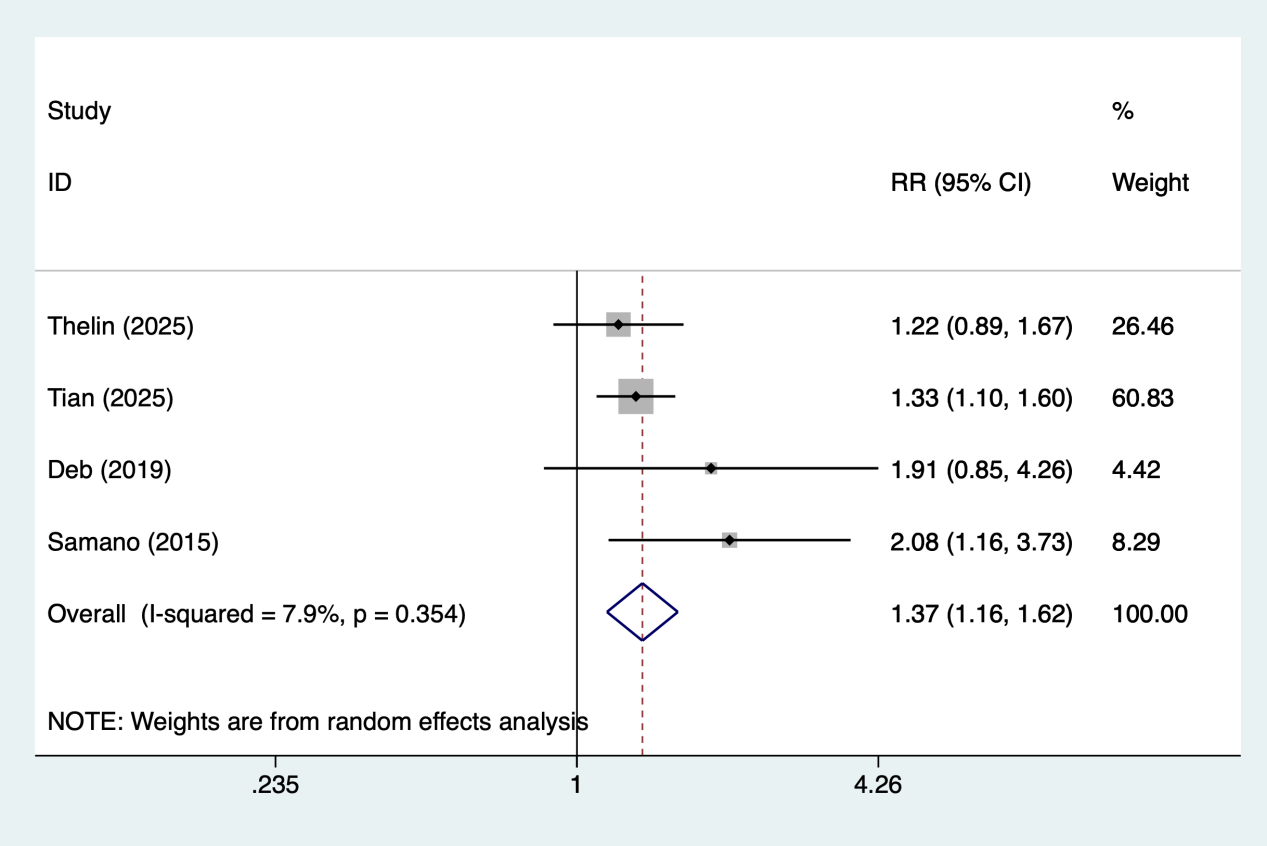


Figure S7-3.Graft failure among OVH vs NT in subgroup analysis. A subgroup analysis was conducted using solely the studies that had a follow-up period of at least 1 year.


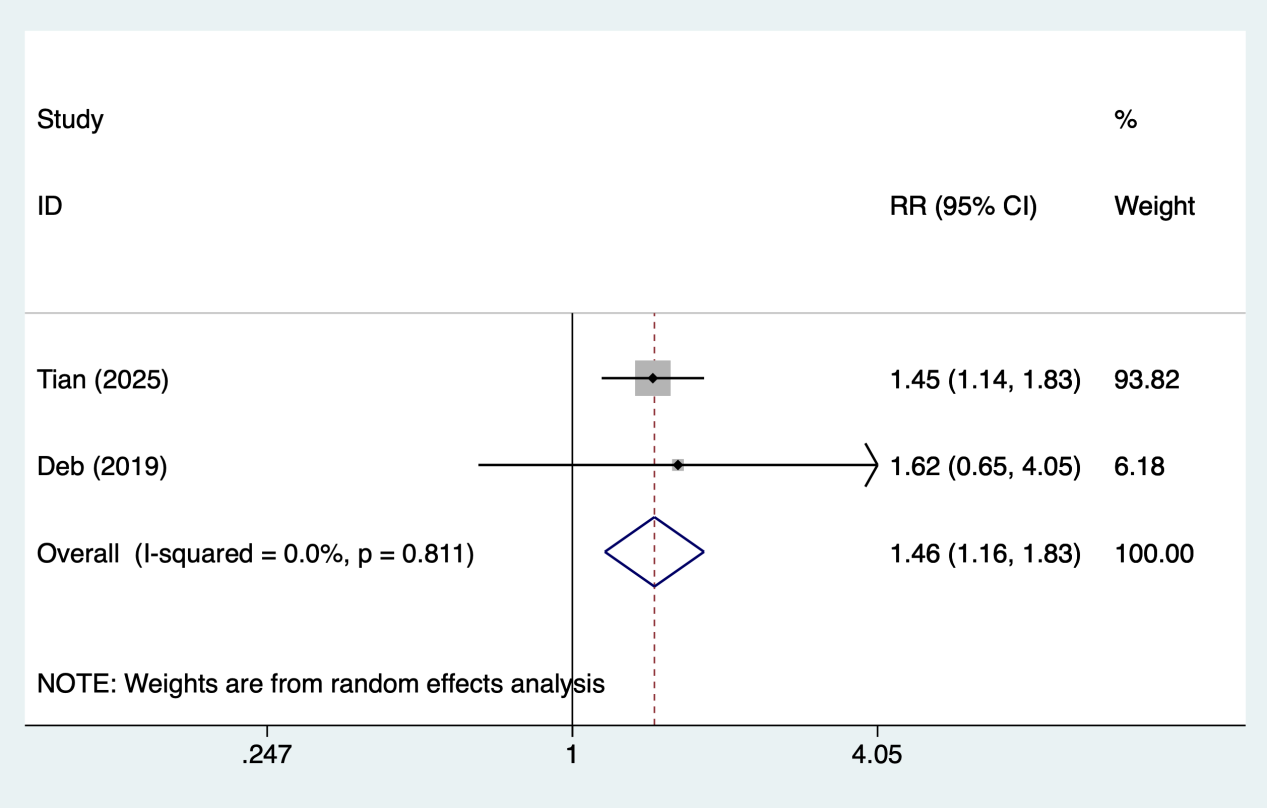


Figure S7-4.Graft occlusion among OVH vs NT in subgroup analysis. A subgroup analysis was conducted using solely the studies that had a follow-up period of at least 1 year.


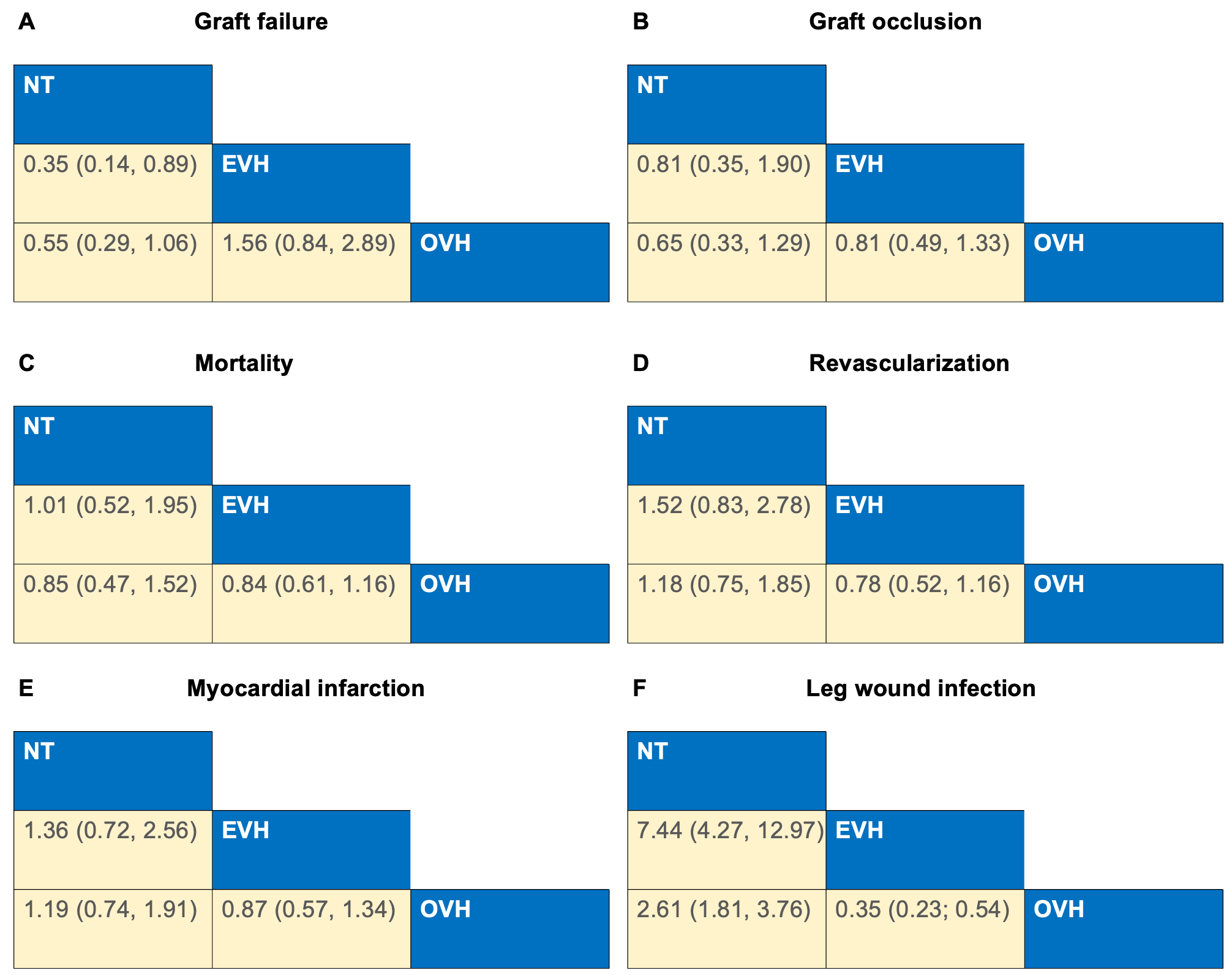


Figure S8. League tables for OVH, EVH, and NT (sensitivity analysis). Sensitivity analyses, conducted by excluding the study (Tian et al.) with the largest sample size. Outcomes shown for (A) graft failure, (B) graft occlusion, (C) mortality, (D) revascularization, (E) myocardial infarction, (D) leg wound infection following OVH, EVH, and NT in subgroup analysis (RR and 95% CI). RR <1 means the treatment in top left is better. OVH = open vein harvesting; EVH = endoscopic vein harvesting; NT = no-touch vein harvesting.
